# MutPred Splice: machine learning-based prediction of exonic variants that disrupt splicing

**DOI:** 10.1186/gb-2014-15-1-r19

**Published:** 2014-01-13

**Authors:** Matthew Mort, Timothy Sterne-Weiler, Biao Li, Edward V Ball, David N Cooper, Predrag Radivojac, Jeremy R Sanford, Sean D Mooney

**Affiliations:** 1 Institute of Medical Genetics, School of Medicine, Cardiff University, Cardiff CF14 4XN, UK; 2 Buck Institute for Research on Aging, Novato, CA 94945, USA; 3 Department of Computer Science and Informatics, Indiana University, Bloomington, IN 47405, USA; 4 Department of Molecular, Cellular and Developmental Biology, University of California Santa Cruz, Santa Cruz, CA 95064, USA; 5 Department of Biomolecular Engineering, University of California Santa Cruz, Santa Cruz, CA 95064, USA

## Abstract

We have developed a novel machine-learning approach, MutPred Splice, for the identification of coding region substitutions that disrupt pre-mRNA splicing. Applying MutPred Splice to human disease-causing exonic mutations suggests that 16% of mutations causing inherited disease and 10 to 14% of somatic mutations in cancer may disrupt pre-mRNA splicing. For inherited disease, the main mechanism responsible for the splicing defect is splice site loss, whereas for cancer the predominant mechanism of splicing disruption is predicted to be exon skipping via loss of exonic splicing enhancers or gain of exonic splicing silencer elements. MutPred Splice is available at http://mutdb.org/mutpredsplice.

## Introduction

In case-control studies, the search for disease-causing variants is typically focused on those single base substitutions that bring about a direct change in the primary sequence of a protein (that is, missense variants), the consequence of which may be structural or functional changes to the protein product. Indeed, missense mutations are currently the most frequently encountered type of human gene mutation causing genetic disease [1]. The underlying assumption has generally been that it is the nonsynonymous changes in the genetic code that are likely to represent the cause of pathogenicity in most cases. However, there is an increasing awareness of the role of aberrant posttranscriptional gene regulation in the etiology of inherited disease.

With the widespread adoption of next generation sequencing (NGS), resulting in a veritable avalanche of DNA sequence data, it is increasingly important to be able to prioritize those variants with a potential functional effect. In order to identify deleterious or disease-causing missense variants, numerous bioinformatic tools have been developed, including SIFT [2], PolyPhen2 [3], PMUT [4], LS-SNP [5], SNAP [6], SNPs3D [7], MutPred [8] and Condel [9] among others. However, the majority of these methods only consider the direct impact of the missense variant at the protein level and automatically disregard same-sense variants as being ‘neutral’ with respect to functional significance. Although this may well be the case in many instances, same-sense mutations can still alter the landscape of *cis*-acting elements involved in posttranscriptional gene regulation, such as those involved in pre-mRNA splicing [10-12]. It is clear from the global degeneracy of the 5′ and 3′ splice site consensus motifs that auxiliary *cis*-acting elements must play a crucial role in exon recognition [13]. To date, a considerable number of exonic splicing regulatory (ESR) and intronic splicing regulatory (ISR) elements have been identified [14-19]. Generally these are classified as either enhancers (exonic splicing enhancers (ESEs)/intronic splicing enhancers (ISEs)) or silencers (exonic splicing silencers (ESSs)/intronic splicing silencers (ISS)), which strengthen and repress, respectively, recognition of adjacent splice sites by the splicing machinery. This distinction may be to some extent artificial in so far as an ESE can act as an ESS and vice versa depending upon the sequence context and the *trans*-acting factor bound to it [16,20]. These *trans*-acting factors include members of the serine/arginine-rich family of proteins (SR proteins) typically known to bind to splicing enhancers and the heterogeneous nuclear ribonucleoprotein family of complexes (hnRNPs), which are thought to bind splicing silencers. However, it is clear that our knowledge of the cooperative and antagonistic elements that regulate pre-mRNA splicing in a context-dependent manner is still very limited [21].

The functional consequences of a splice-altering variant (SAV) may also vary quite dramatically; thus, splicing events that alter the reading frame can introduce premature termination codons that may then trigger transcript degradation through nonsense-mediated decay. Alternatively, an aberrant splicing event may maintain the open reading frame but lead instead to a dysfunctional protein lacking an important functional domain. Even a splice-altering variant that produces only a small proportion of aberrant transcripts could still serve to alter the gene expression level [21].

Up to approximately 14% of all reported disease-causing nucleotide substitutions (coding and non-coding) listed in the Human Gene Mutation Database [1] (11,953 mutations; HGMD Pro 2013.4) are thought to disrupt pre-mRNA splicing whereas 1 to 2% of missense mutations have been reported to disrupt pre-mRNA splicing (HGMD Pro 2013.4). Previous studies have, however, found that the actual proportion of disease-causing missense mutations that disrupt pre-mRNA splicing could be rather higher [22-25]. The difference between the observed and predicted frequencies of disease-causing splicing mutations may be due in part to the frequent failure to perform routine *in vitro* analysis (for example, a hybrid minigene splicing assay [26]), so the impact of a given missense mutation on the splicing phenotype is generally unknown. The likely high frequency of exonic variants that disrupt pre-mRNA splicing implies that the potential impact upon splicing should not be neglected when assessing the functional significance of newly detected coding sequence variants. Coding sequence variants that disrupt splicing may not only cause disease [22] but may in some cases also modulate disease severity [27,28] or play a role in complex disease [29]. The identification of disease-causing mutations that disrupt pre-mRNA splicing will also become increasingly important as new therapeutic treatment options become available that have the potential to rectify the underlying splicing defect [30,31].

Current bioinformatic tools designed to assess the impact of genetic variation on splicing employ different approaches but typically focus on specific aspects of splicing regulation (for example, the sequence-based prediction of splice sites as employed by NNSplice [32] and MaxEntScan [33]) or the sequence-based identification of splicing regulatory elements as exemplified by ESEFinder [14], RESCUE-ESE [15], Spliceman [34] and PESX [19]. Other tools have employed a combination of a sequence-based approach coupled with various genomic attributes - for example, Skippy [35] and Human Splice Finder [36]. In general, however, most tools have not been optimized to deal with single base substitutions, and require the wild-type and mutant sequences to be analyzed separately with the user having to compute any difference in predicted splicing regulatory elements. Tools that are designed specifically to handle single base substitutions include Spliceman, Skippy and Human Splice Finder (HSF). In most cases, as each tool focuses on specific aspects of the splicing code, there is often a need to recruit multiple programs [37] before any general conclusions can be drawn.

An exome screen will typically identify >20,000 exonic variants [38]. This volume of data ensures that high-throughput *in silico* methods are an essential part of the toolset required to prioritize candidate functional variants from the growing avalanche of sequencing data now being generated by NGS. NGS data analysis normally involves applying multiple filters to the data in order to prioritize candidate functional variants. When applying NGS filters, it is important to remember that same-sense variants may alter pre-mRNA splicing via a number of different mechanisms. Hence, a naïve NGS filter that only considers variants within the splice site consensus as candidate splicing-sensitive variants would not identify same-sense variants that caused exon skipping via a change in ESR elements.

Currently, several general areas need to be improved in relation to the identification of genetic variation responsible for aberrant pre-mRNA splicing. Firstly, although the consensus splice site sequences are well defined, the auxiliary splicing elements and their interactions with splice sites are not well understood. Secondly, there is an urgent need for larger unbiased datasets of experimentally characterized variants that alter splicing and have been quantitatively assessed with respect to the mRNA splicing phenotype. This would provide better training data for new models and provide new datasets to benchmark the performance of different tools (both new and existing). Thirdly, there is an urgent need for new bioinformatic tools suitable for use in a high-throughput NGS setting. These tools promise to be invaluable for the comprehensive evaluation of the impact of a given variant on mRNA processing (that is, not just in terms of splice site disruption). It would also be beneficial if the specific consequences for the splicing phenotype (that is, multiple exon skipping, cryptic splice site utilization, and so on) could be accurately predicted so as to reduce our reliance upon expensive and time-consuming *in vitro* analysis. Finally, these high-throughput *in silico* tools should be designed in such a way as to be able to handle different types of genetic variation (that is, coding, non-coding, single base substitutions, microdeletions, microinsertions, and so on) and allow assessment of the combined impact of multiple sequence changes in *cis* (for example, two substitutions within the same exon).

## Materials and methods

### Data sets

For the positive data set (disease-causing splice altering variants (DM-SAVs); Table 1) employed in this study, we identified 1,189 exonic disease-causing/disease-associated mutations from the HGMD (August 2012) [1,39] that were reported (either in the original or a subsequent report) to disrupt pre-mRNA splicing according to the HGMD (Table S1 in Additional file 1).

**Table 1:** Summary of original data sets used in this study

**Data set name**	**Type**	**Description**	**Variants**	**Genes**
Disease-causing splice altering variants (DM-SAVs)	Splice altering variants (SAVs)	Inherited disease-causing coding region mutations that disrupt pre-mRNA splicing, derived from HGMD	1,189	453
Disease-causing splice neutral variants (DM-SNVs)	Splice neutral variants (SNVs)	Inherited disease-causing missense mutations not reported to disrupt splicing derived from the same set of genes as the DM-SAVs. The majority are not expected to have any effect on exon splicing but approximately 25% may nevertheless disrupt splicing	7,729	364
Polymorphic splice neutral variants (SNP-SNVs)	Splice neutral variants (SNVs)	Putatively ‘neutral’ common coding region SNPs (minor allele frequency >0.3) from the 1000 Genomes Project. The majority are not expected to have any effect on pre-mRNA splicing	7,339	3,773

The first negative set of splice neutral variants (SNVs) comprised 7,729 human inherited disease-causing missense mutations from HGMD, not reported to disrupt exon splicing (August 2012) [1,39] and restricted so as to only include mutations from the same set of 453 genes from which the positive set of DM-SAVs were derived. This negative set is referred to as disease-causing splice neutral variants (DM-SNVs; Table 1). It should be noted that whilst the majority of disease-causing missense mutations in this set of DM-SNVs are likely to exert a pathogenic effect via direct disruption to protein structure/function, it would be reasonable to suppose that approximately 25% may disrupt or modulate splicing [23-25].

The second negative set of SNVs comprised 7,339 high frequency exonic SNPs (SNP-SNVs; Table 1), which were compiled from 1000 Genomes Project data [38]. In the SNP-SNV set, only SNPs found with ≥30% minor allele frequency (MAF) in at least one HapMap population from the 1000 Genomes Project data were included. Owing to their high MAF, it is considered unlikely that the majority of these common polymorphisms would have a significant effect on the pre-mRNA splicing phenotype (that is, they may be regarded as being putatively neutral with respect to splicing).

### Training sets

Using the three data sets described above (DM-SAVs, DM-SNVs and SNP-SNVs; Table 1), four different sets of training data were then compiled (Table 2). For the first three training sets, the DM-SAVs constituted the positive set; therefore, the four training sets differed in terms of the choice of negative set of SNVs. For the first training set (Table 2; disease negative set), the negative set comprised 7,729 DM-SNVs. The second training set (Table 2; SNP negative set) used a negative set of 7,339 SNP-SNVs whilst the third training set employed a mixed negative set containing all 7,729 DM-SNVs and all 7,339 SNP-SNVs. Finally, as a control training set (Table 2; Random SNP set), we randomly relabeled 50% of the negative SNP-SNVs as positive examples, generating a training set comprising positive and negative examples exclusively derived from the SNP-SNV data set.

**Table 2:** **Summary of training set sizes derived from the data sets outlined in Table **1

**Training set name**	**Positive set (Iter. 1, Iter. 2, Iter. 3)**	**Negative set (Iter. 1, Iter. 2, Iter 3.)**
Disease negative set	DM-SAVs (1,189, 1,189, 2,601)	DM-SNVs (7,729, 7,363, 31,967)
SNP negative set	DM-SAVs (1,189, 1,189, 2,090)	SNP-SNVs (7,339, 7,253, 70,847)
Mixed negative set (disease and SNP)	DM-SAVs (1,189, 1,189, 6,335)	DM-SNVs and SNP-SNVs (15,068, 14,616, 111,630)
Random SNP set (control)	SNP-SNVs (50%) (3,669, 3,669, 9,901)	SNP-SNVs (50%) (3,670, 3,613, 7,349)

Number of training examples for each different iteration (iter. 1, iter. 2 and iter. 3.) are shown in parentheses.

For the purposes of evaluating a semi-supervised learning approach, three different iterations (Iter. 1, Iter. 2 and Iter. 3) of the original training data were constructed. In the first iteration (Iter. 1), the Random Forest (RF) classification model (see Classification method section for more details) was built using the original four training sets outlined above. Performance was then evaluated with an unseen test set (see Performance evaluation section for more details); the respective model for each training set was then used to build the next iteration (Iter. 2) of the training sets. As the DM-SNV set may contain approximately 25% SAVs, the DM-SNV model built previously in Iter. 1 was then used to identify SAVs in the Disease negative set and SAVs in the SNP negative set identified using the SNP-SNV Iter. 1 model. SAVs predicted with high confidence in both negative sets (DM-SNVs and SNP-SNVs) were then removed and the model retrained to yield Iter. 2. A method for semi-supervised classification termed self-training [40] was then employed to build the next iteration (Iter. 3). Semi-supervised learning typically involves using a small amount of labeled data (for example, DM-SAV) and a large amount of unlabeled data. So, in this instance, the labeling is with respect to impact on splicing (rather than a disease-causing label). The unlabeled data sets comprised the entire HGMD inherited disease data set of 47,228 missense mutations plus the combined data set of missense and same-sense variants identified in the 1000 Genomes Project with no MAF filter applied, that is, includes common and rare variants (192,841 variants). To build the third iteration (Iter. 3), the semi-supervised labeling of variants was based on the second iteration (Iter. 2) model of the respective training sets; this classifier was then applied to the unlabeled data (47,228 disease-causing missense mutations and 192,841 missense and same-sense variants from the 1000 Genomes Project) from which confidently labeled examples were used to supplement the existing training sets used in Iter. 2. A RF classifier was then built with the expanded training sets to complete the third iteration (Iter. 3). It should be noted that the data sets employed here for both training and subsequent analysis only include variants for which all splicing-relevant features could be derived; therefore, variants with missing values were excluded from the data set.

To summarize: iteration 1 (Iter. 1), model built using original training data; iteration 2 (Iter. 2), negative sets (DM-SNVs and SNP-SNVs) had predicted SAVs removed; iteration 3 (Iter. 3), positive and negative sets were supplemented with data labeled from the respective model produced in iteration 2 (Iter. 2).

### Discriminative features investigated in this study

In order to evaluate discriminative features or attributes useful in the identification of exonic single base substitutions that modulate splicing, an array of features were derived based upon the genomic coordinate of the substitution in the human reference assembly (GRCh37/hg19). The majority of existing features employed here were chosen because of prior evidence identifying them as useful in a splicing context [35,41].

Features investigated in this study can be broadly split into three classes: (1) features directly pertaining to the variant under consideration (SNP-based); (2) features associated with the exon (and flanking intron) in which the variant is located (exon-based); (3) features pertaining to the gene in which the variant occurs (gene-based).

### SNP-based features

Ten different types of SNP-based features were selected (see Table 3 for a summary of SNP-based features and how they were constructed). SNP-based features included the distance of the substitution from the nearest splice site (5′ or 3′). To assess the loss and/or gain of ESR elements (ESR change) consequent to a substitution, we employed a previously described method [35] that models the effect of a nucleotide substitution on both the number of ESE and ESS sites created (gained) or abolished (lost) as a consequence of the substitution. Since a number of experimentally or computationally derived sets of ESR (ESE and ESS) motifs have been previously identified, including RESCUE-ESE [15], PESE and PESS [19], Fas-ESS [18], we selected the NI-ESR hexamers [17], comprising 979 ESE motifs and 496 ESS motifs, for use in this analysis. This was because this set had previously been found to provide the strongest signal for identifying exon-skipping variants [35]. The NI-ESR set uses the neighborhood inference (NI) algorithm to identify new ESR motifs based upon previously identified sets of ESR elements (RESCUE-ESE, PESE, PESS and FAS-ESS). A subset of the newly identified ESR motifs predicted by the NI algorithm was then validated using an *in vivo* splicing reporter assay. The ESR change feature was then calculated using a sliding window that covered all hexamers surrounding the variant. Hexamers not present in the NI-ESR set were considered to be neutral. The ESR change comprises nine features derived from the frequency of ESR changes resulting from the substitution: ESE to neutral (ESE loss), ESE to ESE, neutral to ESE (ESE gain), ESE to ESS (ESE loss and ESS gain), neutral to neutral, ESS to ESS, neutral to ESS (ESS gain), ESS to neutral (ESS loss), ESS to ESE (ESS loss and ESE gain).

**Table 3:** Summary of features investigated in this study

**Feature**	**Type**	**Description**
Distance to nearest splice site	SNP-based	Distance between a given variant and the nearest 5′ or 3′ splice site in the target exon.
ESR change	SNP-based	Change in the frequency of ESR elements subsequent to a single base substitution. This includes:
		ESE to neutral (ESE loss)
		ESE to ESE (no change)
		Neutral to ESE (ESE gain)
		ESE to ESS (ESE loss and ESS gain)
		Neutral to neutral (no change)
		ESS to ESS
		Neutral to ESS (ESS gain)
		ESS to neutral (ESS loss)
		ESS to ESE (ESS loss and ESE gain)
In ESE	SNP-based	Frequency of ESE binding sites (in the wild-type) that overlap with the location of the variant
In ESS	SNP-based	Frequency of ESS binding sites (in the wild-type) that overlap with the variant
ESR hexamer score (ESR-HS)	SNP-based	Hexamer scoring function to express the relationship between disease and neutral variants and their differential distributions with respect to loss or gain of an ESE or ESS
Spectrum kernel	SNP-based	Frequency of 3-mers and 4-mers over an 11 bp window (wild type and mutant)
Change in natural splice site strength	SNP-based	MaxEnt splice site score of natural splice site in mutant allele minus MaxEnt splice site score of wild-type allele
Maximum cryptic splice site	SNP-based	Maximum cryptic splice site (5′ and 3′) score (outside of the natural splice site) found overlapping the variant on the mutant allele
Evolutionarily conserved element	SNP-based	PhastCons conserved element probability for substitution site, based on multiple alignments of 46 placental mammals
Base-wise evolutionary conservation	SNP-based	PhyloP base-wise sequence conservation score at site of single base substitution based on multiple sequence alignment of 46 placental mammals
Natural wild-type splice site strength	Exon-based	MaxEntScan score of the natural 5′ and 3′ splice site of the wild-type target exon
Flanking intron size	Exon-based	Length in base-pairs of the upstream and downstream introns flanking the target exon
Intronic ESS density	Exon-based	Intronic ESS density was calculated for 100 bp upstream and 100 bp downstream of the target exon
Exonic ESS density	Exon-based	ESS density was calculated across the first 50 bp and the last 50 bp of the target exon. If the length of the exon was less than 100 bp, then the full length of the exon was used to calculate the ESS density
Exonic ESE density	Exon-based	Same as above but for ESEs
Internal coding exon	Exon-based	{true, false}, Is the target exon an internal coding exon (that is, the target exon is not the first or last coding exon)
Exonic GC content	Exon-based	Percentage of nucleotides that are either guanine or cytosine in the target exon
Exon size	Exon-based	Size of the target exon
Constitutive exon	Exon-based	Is the target exon constitutively spliced
Exon number	Gene-based	Number of exons in the transcript
Transcript number	Gene-based	Number of different reported isoforms that the target gene encodes

To express the relationship between disease and neutral variants and their differential distributions with respect to loss or gain of an ESE or ESS, we constructed a novel ESR hexamer score (ESR-HS) function. This scoring function is outlined in Figure S2 in Additional file 2. To calculate this score, let *t* ∈ {ESE*loss*, ESE*gain*, ESS*loss*, ESS*gain*}, and let S_*t,0,hgmd*_ … S_*t,n,hgmd*_ and S_*t,0,snp*_ … S_*t,n,snp*_ be normalized counts plus a pseudocount for each hexamer in set *t* where n is the number of hexamers such that:

$$\sum_{i}\left(S_{t,i,\text{hgmd}}\right)=1\;\text{and}\;\sum_{i}\left(S_{t,i,\text{snp}}\right)=1\;$$

For some hexamer *k* in set *t*, let H_*t,k,0,hgmd*_ … H_*t,k,5,hgmd*_ and H_*t,k,0,snp*_ … H_*t,k,5,snp*_ be normalized counts plus a pseudocount for position 0 through 5 such that:

$$\sum_{i}\left(H_{t,k,i,\text{hgmd}}\right)=1\;\text{and}\;\sum_{i}\left(H_{t,k,i,\text{snp}}\right)=1$$

Now we define the combined ESR-HS for a specific substitution affecting position *j* of hexamer *k* in set *t*, such that:

$$\text{ESR}‒\text{SH}=\log_{2}\left(S_{t,k,\text{hgmd}}/S_{t,k,\text{snp}}\right)+\log_{2}\left(\;H_{t,k,\text{hgmd}}/H_{t,k,j,\text{snp}}\right)$$

Thus, this ESR-HS is a robust independent combination of the differential strength of the hexamer plus the differential strength of the mutated base in the hexamer.

Another SNP-based feature utilized was the change in natural splice site strength (5′ and 3′) as a consequence of the substitution, as measured by the MaxEntScan algorithm [33]. To model cryptic splice site activation, the maximum splice site score overlapping the variant (not including the natural splice site) found in the mutant RNA sequence was also measured. As it is unlikely that all types of ESR (or other splicing element) have been fully characterized to date, we attempted to overcome this by applying a string-based sequence similarity kernel (the ‘spectrum kernel’), first proposed for classifying protein sequences [42]. By applying the spectrum kernel to both wild-type and mutant sequences, we could identify splicing sequence motifs and measure any changes (loss or gain) consequent to a single base substitution. The spectrum kernel was then applied over an 11 bp window (that is, 5 bp upstream and 5 bp downstream of the variant) using the wild-type genomic RNA sequence to count the frequencies of all k-mers of length = 4 (for example, AGAG, and so on) and length = 3 (for example, GAA); this process was then repeated for the mutant allele. Finally, for SNP-based features, evolutionary conservation based on PhyloP at the position of the substitution [43] and PhastCons [44] was computed, based on the multiple DNA sequence alignments of 46 placental mammal species. The PhyloP score represents a base-by-base (ignores neighboring bases) conservation score for each base of the reference genome. Therefore, PhyloP measures both conservation (slower than expected evolutionary change) and accelerated evolution (faster than would be expected under neutral drift). The PhastCons score represents the probability of the mutated base being located within an evolutionarily conserved element and therefore considers the conservation of the neighboring bases. PhastCons has been used to identify candidate functional elements (for example, splicing factors) in genomic sequences [43]. Both the PhyloP and PhastCons scores were downloaded from the UCSC Genome Browser [45].

### Exon-based features

With respect to the ‘target’ exon within which a given substitution occurred, nine exon-based features were computed. These features included natural wild-type splice site strength (5′ and 3′) using the MaxEntScan algorithm [33], flanking intron size, exon size, exonic GC content, exonic ESE density, exonic ESS density and intronic ESS density. ESE and ESS densities were calculated using a sliding window across the first 50 bp and the last 50 bp of each target exon. Where the length of the exon was <100 bp, then the full length of the exon was used to calculate the ESE and ESS density. Intronic ESS density values between 100 bp upstream and downstream of the relevant exon were calculated in the same manner as the exonic ESE and ESS density. Finally, for the exon-based features, two Boolean features were computed; internal coding exon (the target exon is neither the first nor the last coding exon) and constitutive exon (exon is present in every transcript).

### Gene-based features

Two gene-based features were calculated, the first being the number of exons in the target isoform and the second being the transcript number, which records the number of known protein isoforms that the target gene encodes.

### Feature ranking

**Figure 1:**
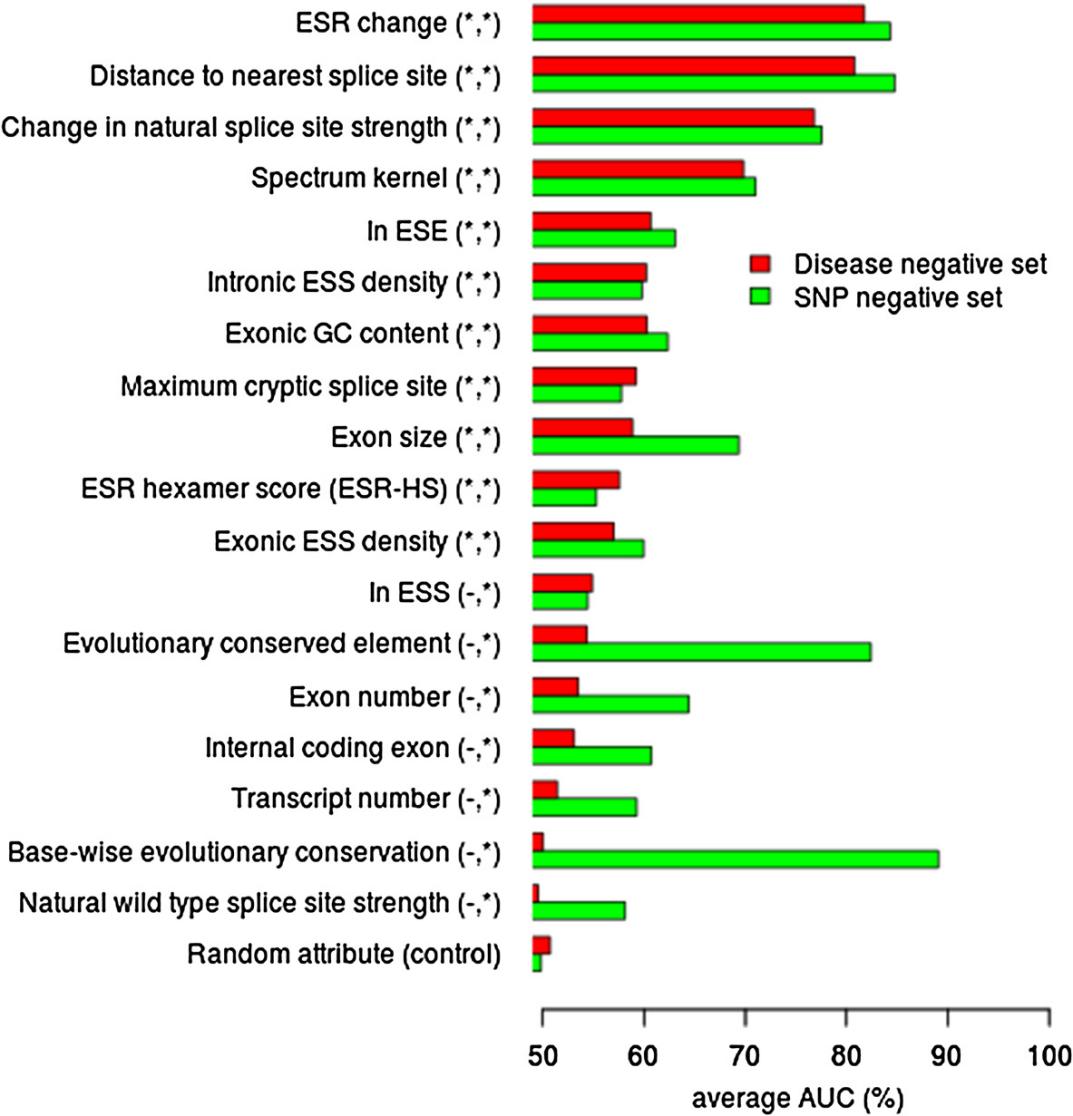
**Feature ranking for Disease negative set versus SNP negative set (Iter. 1), shown by means of the average AUC using 10-fold cross-validation.** The linear support vector machine (SVM) classifier was trained with only the specific feature (or feature subset) that was being tested. As a control, each training example had a randomly generated numerical value computed. AUC values for all features were then compared with the AUC produced by a classifier trained with only the randomly generated attribute by means of a Bonferroni corrected *t*-test (P < 0.05). Significantly different AUC values compared to the random attribute are indicated by asterisks in parentheses for the respective data sets (significant Disease negative set feature, significant SNP negative set feature). Features are ranked by reference to the Disease negative set.

The performance of each feature (or feature subsets) under investigation (Table 3) was evaluated to assess how informative specific features were in discriminating between the DM-SAVs (positive class) and the SNVs (negative class). Feature ranking was then performed on two different sets of training data (Table 2; Disease negative set and SNP negative set; Iter. 1). We evaluated the performance (10-fold cross-validation; linear support vector machine (SVM)) of each individual feature or feature subset by training the ensemble of classification models with only the specific feature being tested. Receiver operating characteristic (ROC) curves and the area under the ROC curve (AUC) were then calculated for each individual feature. A random feature was computed for each training example (numeric value between 0 and 1) and the AUC generated using the random feature alone was used as a control. The AUC from each feature was then compared to the random feature by means of a *t*-test with Bonferroni correction (significance level *P* < 0.05). Features that were significantly different from random in the Disease negative set or SNP negative set are shown in Figure 1.

### Classification method

The supervised classification method employed by MutPred Splice was RF [46], an ensemble method using hundreds of decision trees to perform classification. RF has been extensively used in bioinformatics applications, including the prediction of disease-causing mutations [8,47-49]. The popularity of RF is due in part to its simplicity with no fine-tuning of parameters required and in part to its speed of classification, which is often faster than an equivalent SVM model [50]. In this study, as we are combining multiple classification models and evaluating different training sets, this advantage of RF (limited tuning required) over SVM (tuning required) was considerable. We did nevertheless evaluate RF versus SVM and found that classification performance was broadly similar. SVM is a machine learning model that maximizes the margin of separation between examples of two classes projected into high-dimensional space [51,52]. In this study, we used an SVM with a linear kernel for feature ranking (Figure 1). For the machine learning algorithm implementations, we used LIBSVM and R randomForest package v4.5-36. The Weka toolkit was used for data pre-processing [53].

Generally, it is preferable to use a balanced training set (equal number of positive and negative training examples) to train a supervised classifier, because training on a highly imbalanced data set can be problematic - for example, the classifier can tend to classify most examples as the majority class [54]. In this study, the number of negative examples (DM-SNVs and SNP-SNVs) outnumbered the positive examples by a large margin. To address this inequality and to balance the training sets, we employed an ensemble of RF classification models. This technique was implemented in MutPred Splice by building (in the case of the first iteration of the Disease negative set, for example) different balanced training sets, each with the same positive training set of DM-SAVs, whereas the negative set was randomly sampled (without replacement) from all available negative examples (in that training set) until a balanced set was constructed; this process was then repeated for the next model with the remaining negative DM-SNVs. In MutPred Splice, an RF classifier was then applied to each of the balanced sets of training data, with the final predictive probability being an average of all probability scores produced by each RF classification model. This final predictive probability of a variant disrupting splicing will henceforth be referred to as the general score. This ensemble of RFs approach was then repeated on all four training sets (Table 2).

### Performance evaluation

**Figure 2:**
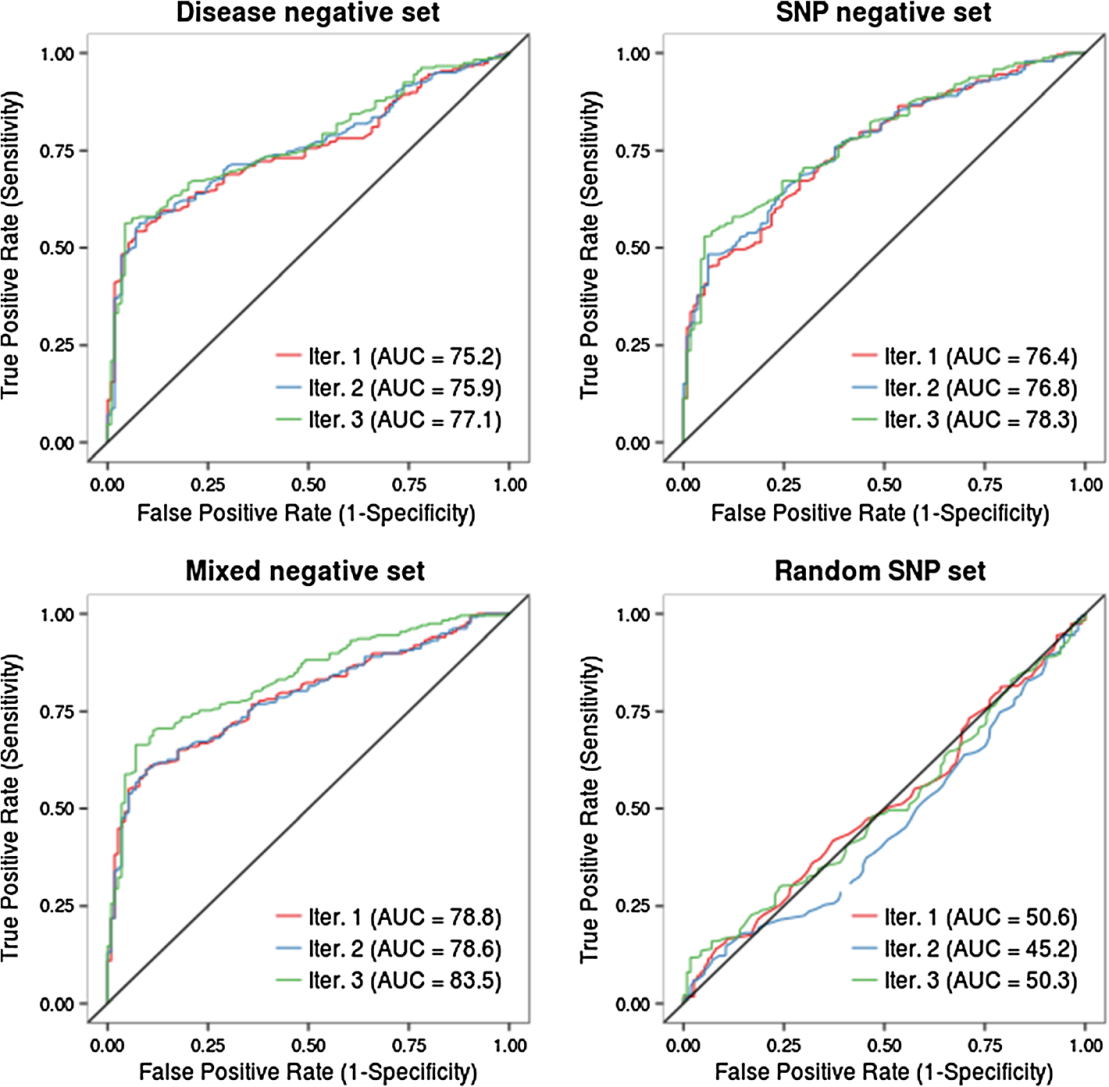
**Model performance evaluation using ROC curves when applied to the same unseen test of 352 variants (238 positive and 114 negative).** For each of the four training sets (Table 2), three different RF classification models were built (Iter. 1, Iter. 2 and Iter. 3). The percentage AUC for each training set and specific iteration are shown in parentheses.

 In order to evaluate the impact of different negative training sets on classification performance, each version of MutPred Splice (built using a different negative set and subsequent iteration; Table 2) was evaluated against the same independently derived experimentally characterized unseen test set (not present in any training data or subsequent iterations thereof). This unseen test set comprised 291 exonic variants (177 positive and 114 negative) experimentally demonstrated to cause either exon skipping, exon retention or cryptic splice site activation and previously compiled by others [35,55-59] and 61 disease-causing exonic splice site (donor -1, acceptor +1) mutations reported in the literature (derived from HGMD). The final unseen test set (Table S2 in Additional file 1) therefore contained 352 variants (238 positive and 114 negative). Using this unseen test set, we were able to establish whether the MutPred Splice predictions were true positives (TP; that is, predicted to disrupt splicing and demonstrated to disrupt splicing experimentally), false positives (FP; that is, predicted to disrupt splicing but shown not to disrupt splicing experimentally), true negatives (TN; that is, predicted not to disrupt splicing and shown not to disrupt splicing experimentally), or false negatives (FN; that is, predicted not to disrupt splicing but shown to disrupt splicing experimentally). This unseen test set approach to validation was favored over cross-validation, because using an unseen test set allows for like-with-like comparisons between the different models produced by the different training sets employed. A MutPred Splice general score probability threshold of ≥0.60 was employed to indicate a predicted SAV. This conservative probability threshold was selected so as to minimize the false discovery rate, albeit at the expense of sensitivity. The performance on this unseen test set was then assessed by plotting ROC curves (Figure 2) and calculating the AUC. A ROC curve displays the true positive rate (or sensitivity) as a function of the false positive rate. We also employed standard benchmarking statistics (Table 4) to evaluate performance such as sensitivity, specificity, accuracy (average of sensitivity and specificity) and the Matthew’s correlation coefficient (MCC) [60]. The MCC was employed since it represents one of the best available measures of prediction quality. It returns a value between -1 and +1; a coefficient of -1 represents the worst possible prediction, 0 a random prediction and +1 a perfect prediction.

**Table 4:** **Standard performance benchmarks for MutPred Splice based on an unseen test set of 352 variants (238 positive, 114 negative) using the three different iterations (Iter. 1, Iter 2. and Iter. 3) of the four different training sets identified in this study (Table **2**)**

**Data set**		**False positive rate (%)**	**Sensitivity (%)**	**Specificity (%)**	**Accuracy (%)**	**AUC (%)**	**MCC**
Disease negative set	Iter. 1	7.0	53.4	93.0	73.2	75.2	0.45
	Iter. 2	7.0	52.5	93.0	72.8	75.9	0.44
	Iter. 3	4.4	55.0	95.6	75.3	77.1	0.49
SNP negative set	Iter. 1	36.8	73.1	63.2	68.1	76.4	0.35
	Iter. 2	36.8	72.3	63.2	67.7	76.8	0.34
	Iter. 3	34.2	71.0	65.8	68.4	78.3	0.35
Mixed negative set	Iter. 1	7.9	56.3	92.1	74.2	78.8	0.46
	Iter. 2	7.9	56.7	92.1	74.4	78.6	0.46
	**Iter. 3**	**7.0**	**64.7**	**93.0**	**78.8**	**83.5**	**0.54**
Random SNP set	Iter. 1	0.0	1.3	100.0	50.6	50.6	0.06
	Iter. 2	0.9	1.7	99.1	50.4	45.2	0.03
	Iter. 3	29.8	31.1	70.2	50.6	50.3	0.01

Classification models were built using RF with 1,000 trees. The unseen test set was experimentally characterized with respect to the splicing phenotype. Performance benchmarks for the final classification model (Mixed negative set; Iter. 3) are highlighted in bold. Performance metrics where appropriate were calculated using a probability threshold (general score) ≥0.60. The Random SNP set is a control set. MCC, Matthews correlation coefficient.

### Experimental characterization of mRNA phenotype

The impact of the inherited disease-causing mutation NM_000051.3: ATM c.5932G > T; NP_000042.3: p.E1978X was assayed in a patient-derived cell line carrying the E1978X mutation or a control cell line (HEK293). Total RNA was extracted from cells using Trireagent LS (Sigma Aldrich, St. Louis, MO USA) and analyzed by RT-PCR using One-Step RT-PCR mix (Invitrogen, Carlsbad, CA USA). Amplicons corresponding to the exon 41 included or skipped product were resolved by agarose gel electrophoresis and visualized by SYBR Gold staining (Figure S1 in Additional file 2).

### Comparison with existing tools used to identify SAVs

MutPred Splice was designed to identify exonic variants that disrupt pre-mRNA splicing via multiple mechanisms: for example, splice site disruption, cryptic splice site activation and exon skipping, and so on. In order to evaluate the performance of MutPred Splice, we opted to compare MutPred Splice with ANNOVAR [61], HSF [36] and Skippy [35]. Although not all the methods evaluated here are directly comparable (since they have different applications and limitations), this selection of tools is nevertheless a fair reflection of the various types of software currently available to identify exonic SAVs. For the purposes of this evaluation, we followed, wherever appropriate and possible, the reported guidelines for performance evaluation of mutation prediction methods [62]. We employed 264 exonic variants (181 positive, 83 negative) derived from the unseen test where predictions could be obtained from all the tools evaluated here. For ANNOVAR and Skippy, the unseen test set included positive SAVs that actually lie outside of the scope of the respective method. Therefore, adjusted performance metrics are also shown using a subset of the overall test set relevant to the specific method. For methods that output multiple scores for a given variant (HSF and Skippy), performance metrics may differ depending upon both the features and the thresholds applied. For a detailed description of guidelines, applications and performance of the tools evaluated here, the reader is referred to the relevant website or original reporting publications.

### Role of pre-mRNA splicing disruption in inherited disease, cancer and polymorphism

To assess the proportion of exonic mutations that disrupt splicing in the context of human inherited disease and cancer, three data sets were compiled (Table 5). First, 61,305 inherited disease-causing exonic mutations from HGMD (August 2012) referred to as ‘Inherited disease’. It should be noted that owing to the inclusion criteria employed by HGMD, the majority of disease-causing same-sense mutations reported in HGMD are putatively splicing-sensitive and so it is expected that a majority of these inherited disease-causing same-sense mutations will be also predicted to disrupt pre-mRNA splicing. Second, 480,730 somatic exonic cancer variants derived from COSMIC [63,64], referred to as ‘Cancer’. A subset of these somatic cancer variants will be drivers (directly implicated in oncogenesis), the remainder being passengers (neutral with respect to cellular proliferation). A third data set comprised 194,241 exonic variants, identified by the 1000 Genomes Project [38] referred to as ‘1000 Genomes’, and was used to compare and contrast with the disease data sets. Unlike the data set employed in training (SNP-SNVs), no MAF filter was applied; therefore, this data set includes both rare and common variants identified in the 1000 Genomes Project. These data sets represent variants for which all required features could be computed; variants with missing values were excluded from the analysis. The MutPred Splice model, built using the Mixed negative set (Iter. 3), was then applied to all three data sets.

**Table 5:** Predicted proportion of exonic variants that disrupt pre-mRNA splicing in human genetic disease (Inherited disease, that is, germline; and Cancer, that is, somatic) and also identified in the general population (1000 Genomes Project participants)

**Data set**	**Proportion of SAVs in data set (predicted SAVs/total variants)**			
	**Missense**	**Same-sense**	**Nonsense**	**Total**
Inherited disease	11.0% (5,193/47,228)	90.3% (468/518)	30.5% (4,130/13,559)	16.0% (9,791/61,305)
Cancer	9.2% (32,056/347,380)	8.6% (9,010/105,094)	32.4% (9,141/28,256)	10.4% (50,207/480,730)
1000 Genomes	6.8% (7,016/103,445)	6.7% (5,968/89,396)	19.5% (273/1,400)	6.8% (13,257/194,241)

The somatic Cancer data set includes driver and passenger mutations recorded in COSMIC [63]. The 1000 Genomes Project data set was derived from the 1000 Genomes Project without any MAF filter having been applied, that is, all rare and common variants were included. The proportion of predicted SAVs for each data set is shown together with the frequencies of predicted SAVs; the sizes of the data sets are shown in parentheses.

### Predicting the splicing mechanism disrupted by a SAV

The prediction of the underlying splicing mechanism disrupted by a SAV (for example, cryptic splice site activation) is based on a previously described method [8], which compares the relevant splicing property with that of the respective distribution of scores obtained from predicted SNVs found in the 1000 Genomes Project. A Z score *P*-value < 0.05 is considered a confident hypothesis.

### Exonic variants in oncogenes and tumor suppressor genes

A list of 71 oncogenes and 54 tumor suppressor (TS) genes were compiled [65]. These two gene sets were then cross-checked against the genes recorded in the datasets used previously (Inherited disease, Cancer and 1000 Genomes with no MAF filter applied). Using these two subsets (oncogenes versus TS) for each of the three data sets, we applied MutPred Splice (Mixed negative set; Iter. 3) to identify the proportion of SAVs in these subsets (Table 6).

**Table 6:** Predicted proportion of exonic variants from two gene subsets (tumor suppressor versus oncogenes) that disrupt pre-mRNA splicing in human genetic disease (Inherited disease that is, germline and Cancer that is, somatic) and also identified in the general population (1000 Genomes project participants)

**Data set**	**Proportion of SAVs in data set (predicted SAVs/total variants)**	
	**Tumor suppressor**	**Oncogenes**
Inherited disease	25.3% (1,130/4,463)	10.9% (132/1,207)
Cancer	16.0% (1,612/10,082)	10.9% (525/4,831)
1000 Genomes	7.4% (84/1,133)	8.0% (49/612)

The somatic Cancer data set includes driver and passenger mutations recorded in COSMIC [63]. The 1000 Genomes Project data set was derived from the 1000 Genomes Project without any MAF filter having been applied, that is, all rare and common variants were included. The proportion of predicted SAVs for each data set is shown, together with the frequencies of predicted SAVs; the sizes of the data sets are shown in parentheses.

### MutPred splice availability

The latest MutPred Splice model is available online at [66] or to download for local installation from [67]. The source code is available from [68]. As new examples of SAVs are reported in the literature, MutPred Splice will be retrained so as to incorporate these additional positive examples of SAVs. This will help to ensure that the model is kept up to date with developments as they are reported in the literature. To facilitate the use of MutPred Splice in an NGS setting, VCF (Variant Call Format) files can be uploaded (or processed locally) for analysis.

## Results

### Identification of informative features for discriminating between SAVs and SNVs

Ranking the features individually using the AUC of the ROC (linear SVM; 10-fold cross-validation) using two different training sets (Disease negative set versus SNP negative set), allowed us to compare and contrast the discriminatory importance of the different features used depending upon the specific negative set being employed (Figure 1). Training the classifier using the Disease negative set identified 11 informative features (Figure 1) that had significantly different AUC values when compared to the AUC produced by a randomly generated attribute (random attribute AUC = 50.7%; *t*-test with Bonferroni correction; *P* < 0.05). For the Disease negative set, the highest ranking features (AUC >70%) were ESR change (AUC of 81.8%), distance to nearest splice site (AUC of 80.8%) and change in natural splice site strength (AUC of 76.8%).

Using a classifier trained with the SNP negative set, we identified 18 informative features (Figure 1) that had significantly different AUC values compared to the AUC produced by a randomly generated attribute (random attribute AUC = 49.8%; *t*-test with Bonferroni correction; *P* < 0.05). For the SNP negative set, the highest ranking features (AUC >70%) were base-wise evolutionary conservation (AUC of 89.1%), distance to nearest splice site (AUC of 84.8%), ESR change (AUC of 84.3%), evolutionarily conserved element (AUC of 82.4%), change in natural splice site strength (AUC of 77.6%) and the spectrum kernel (AUC of 71.0%). Generally, features that performed significantly better than random for the Disease negative set displayed broadly similar performance irrespective of the training set (Disease negative set or SNP negative set) employed. This feature ranking using different negative data sets highlights the importance of evaluating (and experimenting with) different negative data sets, because the choice of training data has a significant impact upon error rate estimation and the ability of the classifier to generalize to other data sets [69].

### Performance evaluation

We evaluated four different training sets (Table 1) and three different iterations of each set (Table 2). These different models were evaluated using a previously compiled unseen set (not present in any training set), for which the variants had been experimentally characterized with respect to their splicing phenotype (SAV or SNV). Figure 2 shows the ROC curves for the four different MutPred Splice classification models, generated using the same unseen test set. In all three iterations (Iter. 1, Iter. 2 and Iter. 3), the Mixed negative set (which combines the Disease negative and SNP negative training data) outperformed the other models within the same iteration with AUCs of 78.8% (Iter. 1), 78.6% (Iter. 2) and 83.5% (Iter. 3). The Mixed negative set also demonstrated the biggest improvement in performance by employing a semi-supervised approach (as judged by the AUC) from Iter. 1 to Iter 3, with a 4.7% AUC increase, compared with both the Disease negative set and the SNP negative set achieving an increase of 1.9%. Standard performance metrics (in addition to the AUC) for all training sets and subsequent iterations are displayed in Table 4. Interestingly, the SNP negative set initially (Iter. 1) had the highest false positive rate (FPR; 36.8%) compared with the Disease negative set (7.0% FPR) and Mixed negative set (7.9% FPR). For all training sets, the semi-supervised approach employed in Iter 3. reduced the initial FPR (Iter. 1) and in the case of both the Disease negative and Mixed negative sets, sensitivity also increased. Therefore, by the third iteration, the Mixed negative set was achieving the highest MCC score of all the training sets (0.54) and the FPR rate had diminished from 7.9% to 7.0%, whilst sensitivity had increased from 56.3% to 64.7%. Based on the results of the evaluation, the Mixed negative classification model (Iter. 3) with a 7.0% FPR, 64.7% sensitivity, 93.0% specificity, 83.5% AUC and 0.54 MCC was selected as the final MutPred Splice classification model. Therefore, all further analysis was performed using this final predictive model.

### Case studies

Two inherited disease-causing mutations (neither one of which was present in either the training data or unseen test sets) were selected as case studies. These case studies were used for further additional evaluation of both the semi-supervised approach and the final predicative model (Iter. 3). For these mutations, there was no prior evidence from *in vitro* analysis for or against an impact on splicing, when the mutation was originally reported but subsequent experimental characterization provided evidence of a splicing defect [25,70] (Figure S1 in Additional file 2).

**Figure 3:**
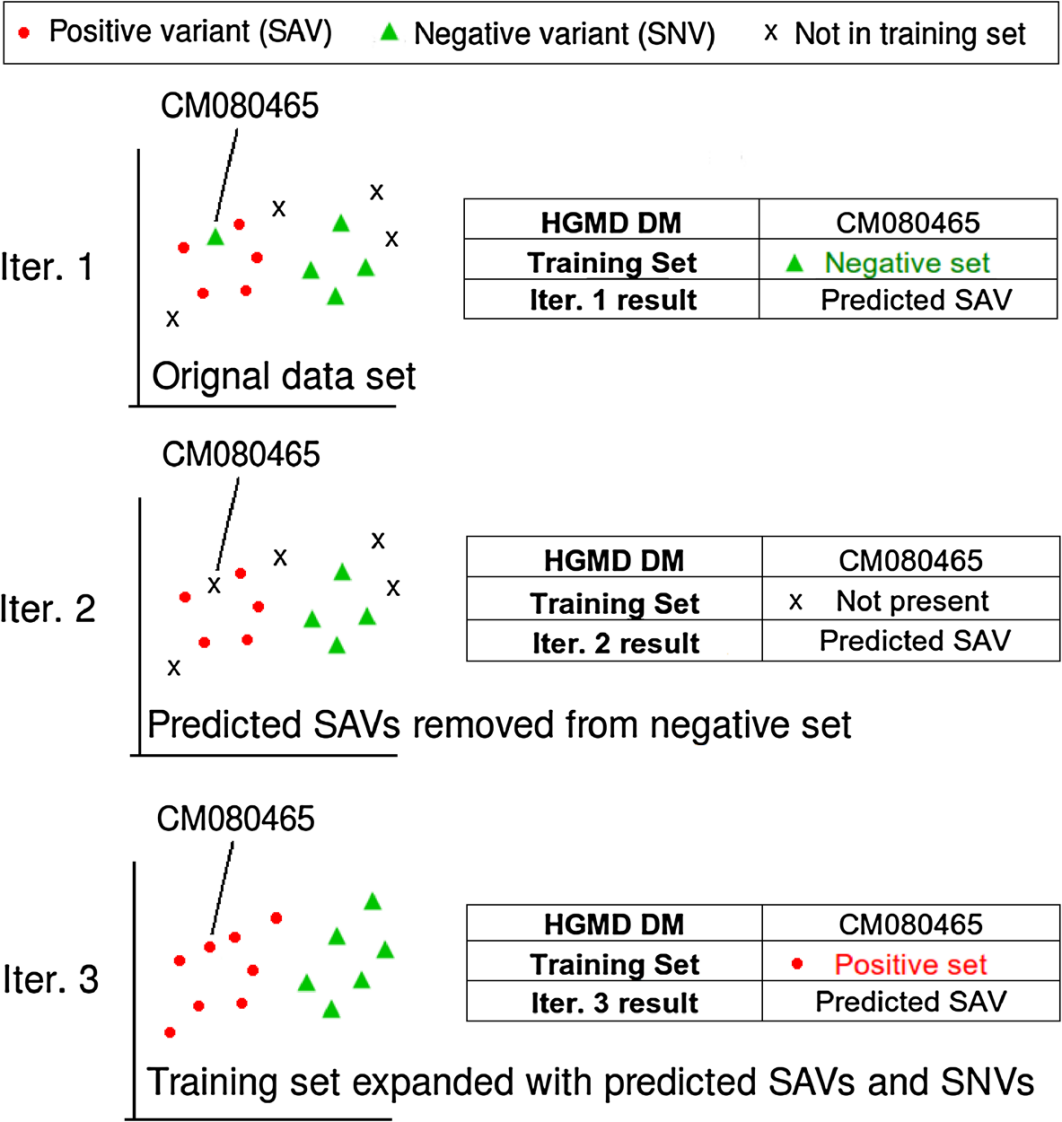
**Case study illustrating the semi-supervised approach employed in this study.** The disease-causing (DM) missense mutation CM080465 in the *OPA1* gene (NM_015560.2: c.1199C > T; NP_056375.2: p.P400L) was not originally reported to disrupt splicing but was later shown *in vitro* to disrupt pre-mRNA splicing [25]. CM080465 was included in the negative set in the first iteration (Iter. 1). The Iter. 1 model, however, predicted CM080465 to disrupt pre-mRNA splicing (SAV). In the next iteration (Iter. 2), CM080465 was excluded from the negative set. The Iter. 2 model still predicted CM080465 to be a SAV and so, in the final iteration (Iter. 3), this variant was included in the positive set. This demonstrated that a semi-supervised approach can, at least in some instances, correctly re-label an incorrectly labeled training example. SAV, splice-altering variant; SNV, splice neutral variant.

To evaluate the semi-supervised approach, a disease-causing missense mutation in *OPA1* (NM_015560.2:c.1199C > T, NP_056375.2:p.P400L), which we had shown previously by *in vivo* assay to result in a 47% decrease in target exon inclusion [25], was selected. This positive training example was then deliberately included as a negative example in the DM-SNV set and our iterative approach successfully removed this mutation from this negative training set in Iter. 2 and then correctly relabeled it as a positive training example in the third iteration of the model (Iter. 3; Figure 3).

### Comparison with existing tools used to identify SAVs

MutPred Splice performance using the full unseen test set is summarized in Table 4. Here we focus on the comparison of MutPred Splice with three other tools; ANNOVAR [61], HSF [36] and Skippy [35] (Table 7). All tools evaluated here are designed for (but not limited to) the analysis of exonic variants on pre-mRNA splicing. ANNOVAR is a popular tool designed for the functional annotation of genetic variants identified in NGS studies. ANNOVAR identifies potential splice site SAVs based on the presence of a particular variant within a splice site (binary label, presence or absence within a splice site). Employing this test set of 264 variants, ANNOVAR achieved an overall sensitivity of 22.7%, a specificity of 95.2% and an MCC of 0.22 (Table 7). For the adjusted ANNOVAR performance where the positive test set was limited to variants that abolish the natural splice site only, ANNOVAR identified all of the splice site SAVs (adjusted sensitivity of 100.0% and MCC of 0.93; Table 7), but as the ANNOVAR splicing prediction is based on location alone (that is, presence in splice site), any potential splice site SAV should then be assessed with another tool such as HSF or MaxEntScan [33] to provide further supporting evidence that the variant abolishes the natural splice site. HSF is an online tool used to identify the effect of genetic variation on a comprehensive range of known splicing signals, including splice sites and different sets of ESEs and ESSs. HSF represents a powerful tool for investigating the underlying mechanism responsible for a given splicing defect, but owing to the number and range of different splicing signals that can be investigated, interpretation of the data can be difficult. Skippy is a tool designed to detect exonic variants (outside the splice site) that modulate splicing. Skippy’s focus is on variants that cause exon skipping via changes to ESEs/ESSs or create cryptic splice sites. Overall, Skippy demonstrated an MCC of 0.19, which was comparable to the overall (unadjusted) ANNOVAR performance. For Skippy, restriction to a positive test set of exon skipping and cryptic splice site-activating variants demonstrated increased performance with an MCC of 0.34.

**Table 7:** Comparison of three existing tools used to identify exonic SAVs with MutPred Splice

**Method**	**ANNOVAR**	**Human splicing finder**	**Skippy**	**MutPred splice**
Splicing focus	Splice site disruption	All exonic and intronic	ESE/ESS disruption and cryptic splice site	All exonic
Prediction output	Binary label	Multiple output scores	Multiple output scores	Probabilistic, with additional hypothesis of splicing mechanism disrupted
TP	41	65	68 (61)	121
FP	4	33	15	7
TN	79	50	68	76
FN	140 (0)	116	113 (57)	60
FPR%	4.8	39.8	18.1	8.4
Sensitivity (%)	22.7 (100.0)	35.9	37.6 (51.7)	66.9
Specificity (%)	95.2	60.2	81.9	91.6
Accuracy (%)	58.9 (97.6)	48.1	59.7 (66.8)	79.2
MCC	0.22 (0.93)	-0.04	0.19 (0.34)	0.54

Evaluation was based on 264 exonic variants (181 positive, 83 negative). Performance metrics are given for guidance only as not all tools may be directly comparable (due to different applications or limitations). Performance scores in parentheses reflect adjusted performance based upon the evaluation of only specific categories of splicing mutation (for example, splice site disruption) relevant to the respective tool. For methods that output multiple scores for a variant (HSF and Skippy), performance metrics may differ depending upon the features and thresholds applied. TP, true positives; FP, false positives; TN, true negatives; FN, false negatives; FPR, false positive rate; MCC, Matthews correlation coefficient.

All tools evaluated here demonstrated utility when investigating and identifying SAVs. This notwithstanding, overall, MutPred Splice outperformed the other tools evaluated here with sensitivity of 66.9%, specificity of 91.6% and an MCC of 0.54 (Table 7). For both HSF and Skippy, multiple output scores are produced; however, since none are diagnostic on their own, manual interpretation is often required to assess the weight of evidence that a variant is a potential SAV. The strength of HSF lies in its detailed investigation into the underlying splicing signals that may be disrupted; it is therefore complementary to MutPred Splice. For example, MutPred Splice could be used to generate a hypothesis for an exonic SAV, followed by detailed investigation using HSF. In general, it is important that the user is aware of the limitations and applications of a specific tool, when using that method to interpret their data. Depending upon the application, we recommend using multiple methods, especially tools that are complementary to each other.

### Mis-splicing as a functional consequence of exonic variants

**Figure 4:**
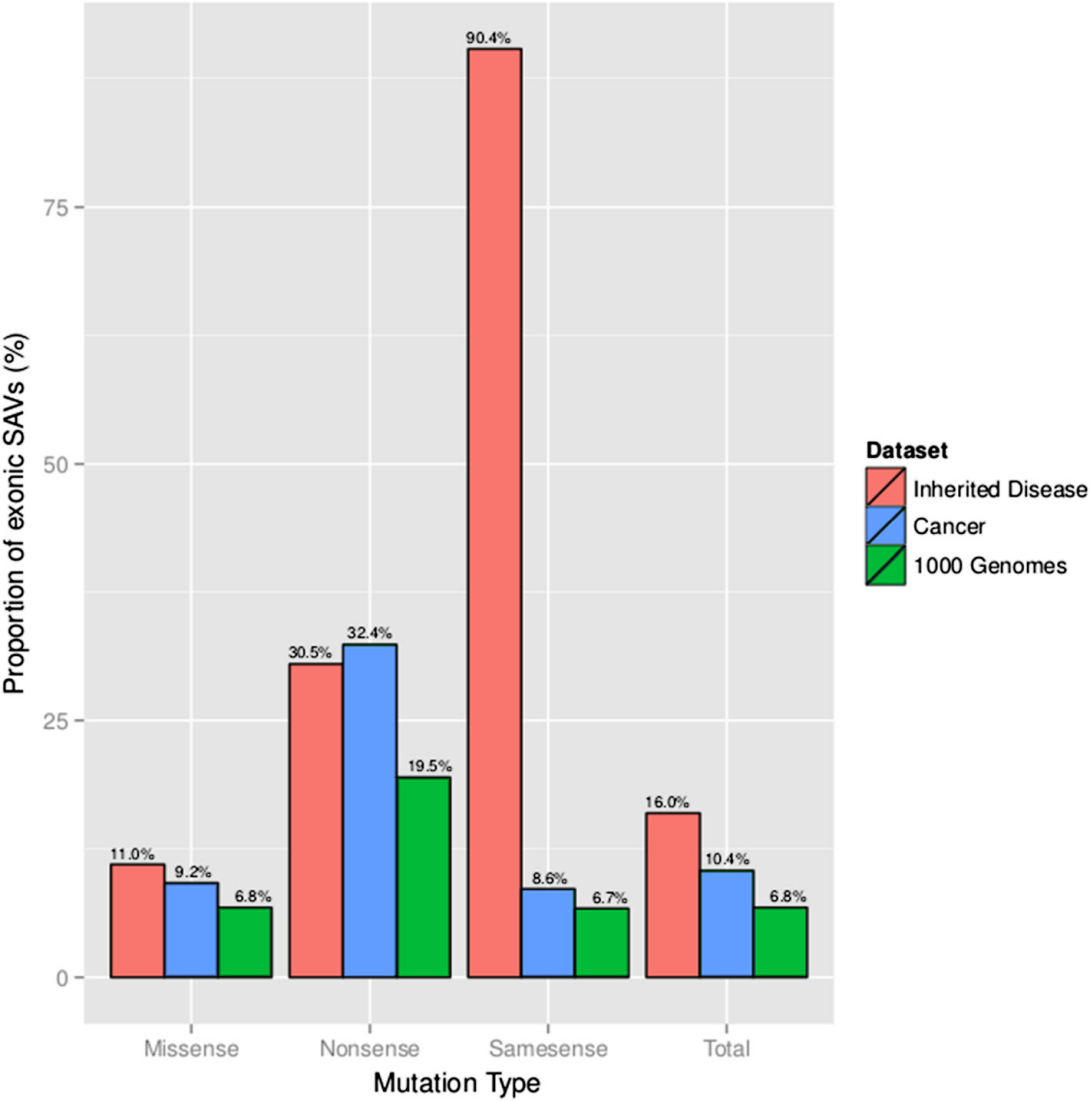
**Role of exonic variants in aberrant mRNA processing for Inherited disease and Cancer data sets.** The somatic Cancer variants were derived from COSMIC and include both driver and passenger mutations. For all mutation types and the combined total, the proportions of predicted SAVs in both Inherited disease and Cancer were significantly enriched (Fisher’s exact test with Bonferroni correction applied; *P* < 0.05) when compared to exonic variants identified in the 1000 Genomes Project (unlike the SNP negative training set, in this instance no MAF filter was applied, that is, all rare and common variants were included).

To assess the extent of mis-splicing as a functional consequence of exonic variants (missense, same-sense and nonsense), the final MutPred Splice model was applied to three data sets; inherited disease-causing mutations from HGMD, somatic cancer-associated mutations (including drivers and passengers) from COSMIC, and exonic variants identified in the 1000 Genomes Project (Figure 4). Overall, inherited disease (16.0% of the data set) and cancer (10.4% of the data set) were significantly enriched for predicted SAVs compared to variants found in the general population (1000 Genomes Project; no MAF filter applied; 6.8%; Fisher’s exact test with Bonferroni correction; *P* < 0.05). We see similar enrichment trends when we separate each data set into the different subtypes of coding-region variant (missense, same-sense and nonsense). With respect to missense variants, 11.0% of Inherited disease mutations and 9.2% of Cancer mutations were significantly enriched for SAVs compared to 6.8% from variants identified in the 1000 Genomes Project data (Fisher’s exact test with Bonferroni correction; *P* < 0.05). For same-sense mutations, 90.3% of inherited disease mutations are predicted to be SAVs, whereas the remaining 9.6% may have an impact upon other mechanisms of pathogenesis (for example, through codon usage). Predicted same-sense SAVs in the Cancer data set were significantly enriched when compared to the 1000 Genomes Project same-sense variants (8.6% versus 6.7%; Fisher’s exact test with Bonferroni correction; *P* < 0.05). Nonsense mutations in disease (both Cancer and Inherited) were more highly enriched for exonic variants responsible for splicing defects than nonsense variants identified in 1000 Genomes Project data (30.5% and 32.4% versus 19.5% respectively; Fisher’s exact test with Bonferroni correction; *P* < 0.05). When looking at the different types of mutation (missense, same-sense and nonsense), we find that a nonsense mutation is approximately three-fold more likely to elicit a splicing defect compared to a missense or same-sense mutation. This result is consistent with what has been shown previously [18,25] and has been attributed to the inherent sequence bias of ESE loss and ESS gain towards nonsense mutations. It is important to note that a nonsense mutation may affect pre-mRNA splicing before it can impact on mRNA export or translation. Although the resulting aberrant transcript may still be bound for degradation by nonsense-mediated decay, it may be due to a splicing induced frame-shift rather than the original nonsense mutation recognized as a premature termination codon. For exonic variants identified in the general population, a missense or same-sense variant is equally likely to elicit a splicing defect.

### Predicting the splicing mechanism disrupted by an SAV

**Figure 5:**
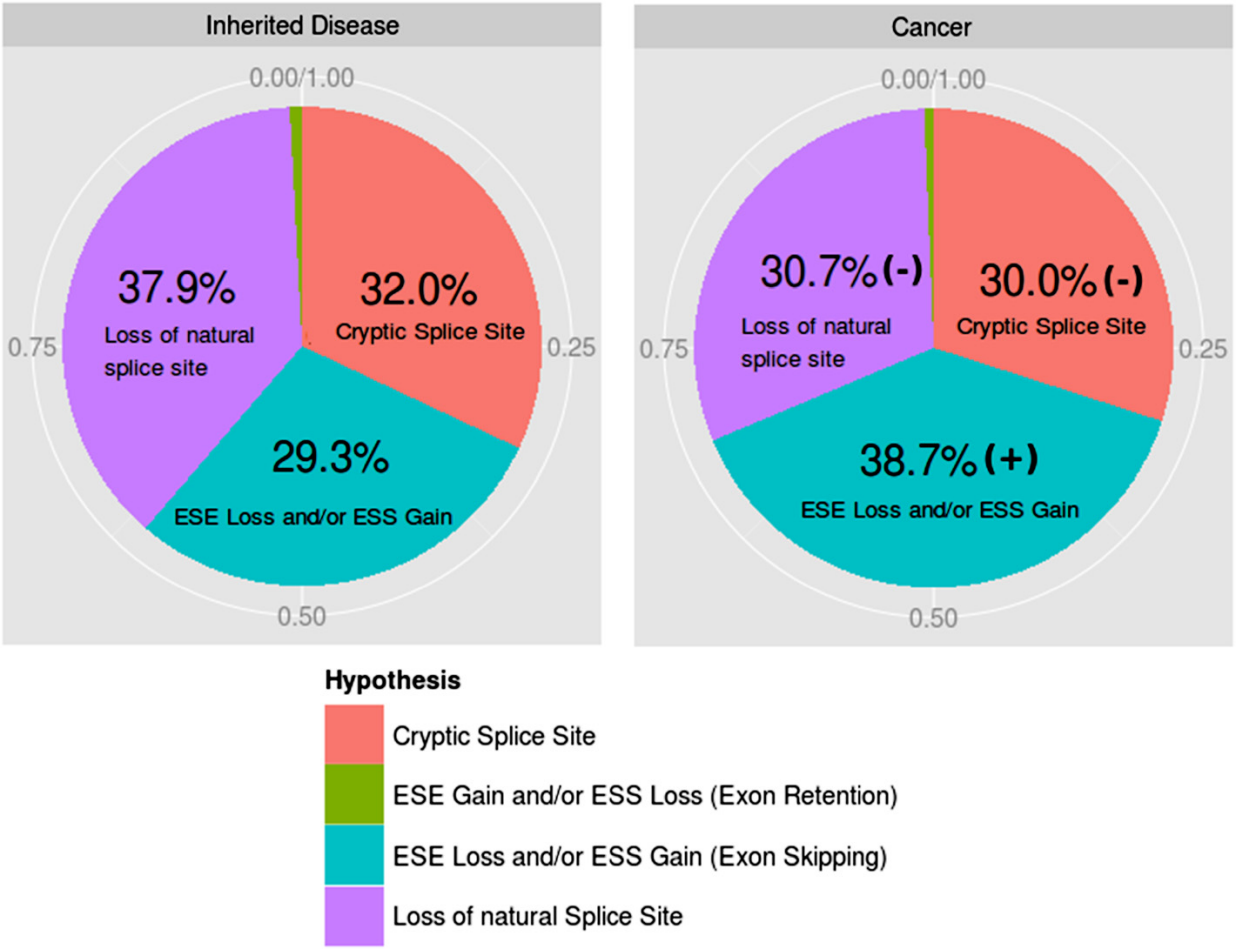
**Confident hypotheses of the underlying splicing mechanism disrupted for predicted exonic SAVs in Inherited disease and somatic variants in Cancer.** Significant enrichment (+) or depletion (-) for a specific hypothesis is shown for the Cancer versus Inherited disease datasets (Fisher’s exact test with a Bonferroni-corrected threshold of *P* < 0.05).

Using MutPred Splice, confident hypotheses for the underlying mechanism of splicing disruption were made for the majority of SAVs in Inherited disease (63.5%) and Cancer (66.3%) (Figure 5). In Inherited disease, the main underlying splicing mechanism disrupted was loss of the natural splice site accounting for 37.9% of SAVs, followed by cryptic splice site activation with 32.0%. ESE loss and/or ESS gain leading to exon skipping was predicted for 29.3% of SAVs. Exon retention of an alternative exon was predicted to be the splicing defect in only 0.8% of SAVs. By contrast, for SAVs in Cancer, the predominant mechanism was ESE loss and/or ESS gain (38.7%), with Cancer being significantly enriched for SAVs causing ESE loss and/or ESS gain compared to Inherited disease (Fisher’s exact test with Bonferroni correction applied; *P* < 0.05).

### Exonic SAVs in oncogenes and tumor suppressor genes

**Figure 6:**
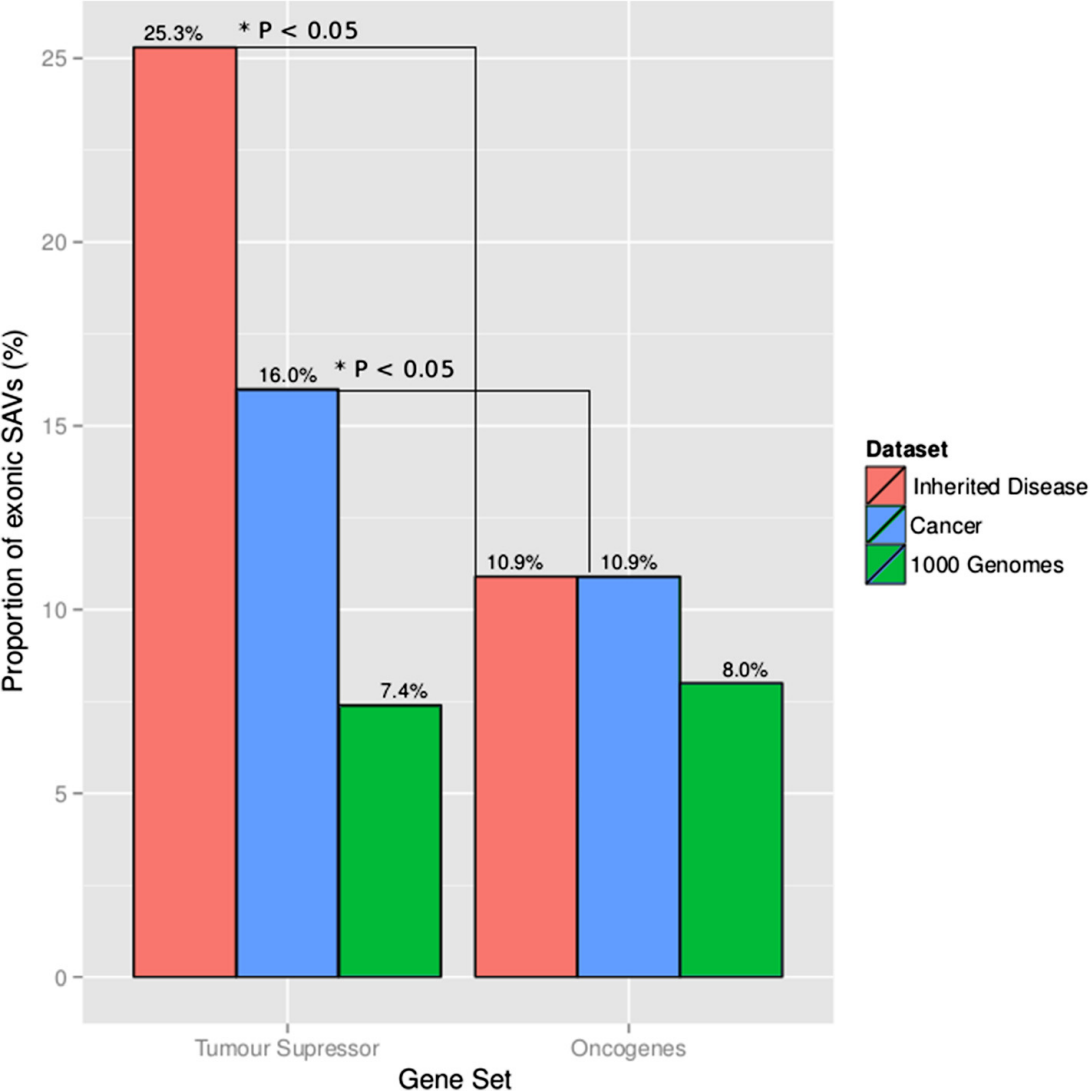
**Proportion of exonic variants involved in aberrant mRNA processing for a set of tumor suppressor genes (71 genes) and a set of oncogenes (54 genes), from three different data sets (Inherited disease, somatic mutations in Cancer, and variants identified in the 1000 Genomes Project with no MAF filter applied, that is, all rare and common variants included).** Disease-causing substitutions in tumor suppressor (TS) genes tend to be recessive loss-of-function mutations, in contrast to disease-causing substitutions in oncogenes, which are usually dominant gain-of-function mutations. Inherited disease and Cancer are significantly enriched in the TS gene set (denoted by an asterisk), when compared with the equivalent set of oncogenes, for mutations that are predicted to result in aberrant mRNA processing (SAVs). *P*-values were calculated using a Fisher’s exact test with a Bonferroni-corrected threshold of *P* < 0.05.

Sets of 71 oncogenes and 54 TS genes were selected as described in Materials and methods. Disease-causing mutations in TS genes tend to be recessive loss-of-function (inactivating), in contrast to mutations in oncogenes, which are usually dominant gains-of-function (activating). The numbers of reported variants in these two gene sets (oncogenes versus TS) are given in Table 6. When comparing each gene set within the same data set (Inherited disease, Cancer and 1000 Genomes), we see that exonic variants in Inherited disease (25.3%) and Cancer (16.0%) are significantly enriched for SAVs in TS genes compared to oncogenes (Figure 6). This enrichment for SAVs in TS genes is not found when looking at variants that are present in the general population (1000 Genomes). These data suggest that aberrant pre-mRNA splicing may be a common mechanism for inactivation of TS genes. Including the data presented in Figure 5, we propose the provocative hypothesis that TS gene architecture may be particularly ‘fragile’ in the sense that they have both inflated proportions of SAVs and higher rates of loss/gain of ESR elements than other genes. If this hypothesis is correct, then when we attempt to identify somatic drivers in cancer in an NGS setting, the potential impact of all types of exonic variant (missense, same-sense and nonsense) on pre-mRNA splicing should be highlighted rather than neglected. Future studies that investigate the aspects of gene architecture that are responsible for an increased susceptibility to aberrant pre-mRNA splicing may illuminate the validity of this hypothesis.

## Discussion

There is abundant evidence to suggest that, for both coding and non-coding variants, aberrant pre-mRNA splicing is a common mechanism of pathogenesis in both inherited disease and cancer. In order to predict potential disease severity from genotype data, it is necessary to comprehensively evaluate the potential functional impact of variants. Effective computational tools targeted towards the characterization of the impact of variants on posttranscriptional gene regulatory function are urgently required. Here we have developed and evaluated a novel computational model (MutPred Splice) that uses human disease alleles for training to predict exonic nucleotide substitutions that disrupt pre-mRNA splicing. This approach is complementary to other methods that utilize known splice sites or functional variants that have not been directly associated with disease. Since MutPred Splice predictions can be made for missense, same-sense and nonsense variants, this tool significantly expands the scope of existing tools, which tend to focus almost exclusively on missense variants. When applied in an NGS setting, MutPred Splice is designed to be run in parallel with other methods such as SIFT [2] or SNAP [6], which identify missense mutations that are likely to disrupt protein structure/function; however, it adds an additional degree of resolution because MutPred Splice is also able to assess same-sense variants, which are typically excluded by the majority of current NGS filtering strategies. Since we predict that approximately 7% of same-sense variants disrupt pre-mRNA splicing, it is clear that this class of variant should not be blithely dismissed from the outset as being neutral to function.

### Training data

In this study, we have highlighted the difficulty in selecting an appropriate negative training set. Since the underlying training data are fundamental to any derived model, it is clear that selecting the appropriate negative (control) set is of equal importance to selecting the appropriate positive set. In this study, we found that high frequency SNPs are a valuable source of training data but combining high frequency SNPs with an additional negative set of inherited disease-causing mutations serves to increase the diversity in the training set and reduces the FPR of the model, which results in improved performance over a model built using either negative set on its own.

### Classification performance

The lack of experimental splicing data for the majority of disease-causing missense mutations means that the vast majority of this data set is untested with respect to their impact on the mRNA splicing phenotype (positive or negative). To mitigate this unknown quantity, in the second iteration of our models we removed predicted SAVs from the negative set; however, model performance remained broadly constant. This demonstrates that the RF ensemble approach used throughout all iterations to balance the positive and negative sets also reduces the impact of noise in the negative set. Based on previous studies that found that approximately 25% of disease-causing missense mutations may disrupt splicing [23-25], we adopted a semi-supervised self-training approach in an attempt to label these unlabeled mutation data. This allowed us to utilize these unlabeled data in a novel way, increase the training set size and improve the identification of decision boundaries between positive and negative classes. Indeed, utilization of semi-supervised learning in this instance saw a performance increase for all models (Disease negative set, SNP negative set and Mixed negative set). Self-training does have its limitations and mistakes in the first iteration could be subsequently reinforced; to mitigate this, only confident labels were applied to expand the training sets. For the final MutPred Splice model, we selected the third iteration of the Mixed negative set, which when coupled with a conservative probability threshold (at the expense of sensitivity) becomes a useful model for prioritizing SAVs, especially in an NGS setting, with a FPR of 7.0%, sensitivity of 64.7% and specificity of 93.0%, AUC of 83.5% and an MCC of 0.54.

### Exonic SAVs in inherited disease and cancer

Based on previous work and also as demonstrated here, disruption to pre-mRNA splicing via exonic substitutions underlies a large proportion of inherited disease and cancer mutations. Here we estimate, based on the sensitivity and specificity of our model, that approximately 16% of inherited disease and approximately 10 to 14% of cancer exonic mutations impact upon pre-mRNA splicing, probably as a primary mechanism for pathogenicity. This is broadly in line with the results of previous studies. It should be noted, however, that the cancer set analyzed will contain a large proportion of passenger variants, which will almost certainly lead to a serious under-estimation of the actual number of splicing-sensitive cancer driver mutations.

In recent years, evidence for the link between cancer development and aberrant splicing has grown [71,72]. In this study, we have found that TS genes are significantly enriched (when compared to oncogenes) in predicted exonic splicing mutations in both inherited disease and cancer. This enrichment is not found in variants identified in the general population (Figure 6; 1000 Genomes Project data with no MAF filter applied). Interestingly, the disease-causing nonsense variant in the *ATM* gene (p.E1978X), which is experimentally demonstrated to cause exon skipping, was originally reported as causing ataxia telangiectasia [73] but has in addition been associated with breast cancer susceptibility [74].

Aberrant pre-mRNA splicing in TS genes caused by exonic variants may represent a common mechanism of TS gene inactivation, thereby contributing to oncogenesis. Whilst a role for aberrant splicing leading to TS gene loss-of-function is not altogether novel [75], the scale and potential involvement of splice-altering exonic variants in oncogenesis is not well studied. The Cancer dataset has an increased tendency towards loss of ESE and/or gain of ESS elements (compared to the Inherited disease dataset). This finding could be explicable in terms of an increased susceptibility of TS genes to aberrant splicing.

### Variants affecting pre-mRNA splicing in the general population

Here we have shown that around 7% of exonic variants found in the general population may alter splicing. Such variants may exert their effects in different ways, from a subtle change that serves to modify gene expression levels, to a lesion that results in the complete deficiency of the functional protein product. In some cases, therefore, the impact of common variants on splicing may not have an obvious phenotypic effect but could nevertheless serve to modulate disease risk, especially in the context of complex disease; alternatively, it may act as a disease modifier. Interestingly, not all nonsense variants can be considered equal with respect to their impact on splicing. A nonsense mutation identified in the context of inherited disease or cancer is predicted to be approximately twice as likely to elicit a splicing defect when compared to a nonsense variant found in the general population. Since this study was initiated and the training set compiled, six variants that were initially found in the general population (1000 Genomes Project), and which MutPred Splice predicted to disrupt pre-mRNA splicing, have been subsequently reported as disease-causing, disease-associated or of functional significance (according to HGMD). For example, a predicted SAV in the *NPR3* gene (NM_000908.3: c.1429G > A; NP_000899.1: p.G477S) is associated with reduced NPR3 protein expression [76]. Another example is a predicted SAV in the *MACF1* gene (NM_012090.4: c.6868A > G; NP_036222.3: p.M2290V), which has been reported in association with type 2 diabetes [77]. Interestingly, all six of these predicted SAVs were also predicted to be tolerated by SIFT, highlighting the importance of using MutPred Splice in conjunction with other tools specifically designed to identify missense mutations that disrupt protein structure and/or function (for example, SIFT and Polyphen2, and so on).

### Limitations

Whilst the positive training set of SAVs employed here constitutes the largest available dataset of its kind, it is likely that a larger positive training set would be of considerable benefit. The other limitation is the ‘noise’ from actual SAVs in the Disease negative set. The semi-supervised approach was therefore employed to counteract these limitations. The MutPred Splice model will be retrained as more training data become available in the literature. Additionally, our current knowledge about the splicing code is still incomplete; for example, approximately 9% of exon skipping SAVs displayed no obvious changes in ESE/ESS elements [35], indicating that novel *cis*-acting splicing regulatory elements probably remain to be discovered. As our knowledge in this field advances, more informative features can be derived and incorporated.

Another limitation of our tool is the assumption that the single exonic variant that is being assessed for aberrant splicing is the only deviation between the relevant reference sequence (RefSeq), with no other relevant sequence changes being present. To illustrate this point, only 4.8% of patients in a large cohort of Duchenne muscular dystrophy patients were found to exactly match the coding region of the *DMD* gene with respect to the reference sequence [78]. Therefore, when considering the impact on the splicing code, it may be in some cases too simplistic to consider just one variant in isolation, because other sequence changes (in *cis*), within both the coding and non-coding regions, may strengthen or weaken exon definition; the resulting combined impact is therefore difficult to predict.

We note that statistically rigorous estimation of the fraction of variants (in a particular set) that disrupt splicing is a very difficult problem, caused by potentially biased training data combined with a general inability to achieve 100% classification accuracy. As the correction of sample selection bias is generally hard, in this work we chose to report the fraction of positive predictions by MutPred Splice as our best estimate.

## Conclusion

We have used the most comprehensive splicing mutation data sets currently available to build a computational model to predict exonic substitutions that disrupt pre-mRNA splicing. To do this, we have adopted a machine learning approach using semi-supervised learning and have evaluated a combination of sequence-based and genomic attributes to build a new tool, MutPred Splice, to identify coding region splice-altering variants responsible for either somatic or inherited disease. This model is suitable for use in an NGS high-throughput setting to identify and prioritize potentially splice-altering variants that may be involved in both inherited disease and cancer.

## Abbreviations

AUC: area under the receiver operating characteristic curve; bp: base pair; DM-SAV: disease-causing splice altering variant; DM-SNV: disease-causing splice neutral variant; ESE: exonic splicing enhancer; ESR: exonic splicing regulatory; ESR-HS: exonic splicing regulatory hexamer score; ESS: exonic splicing silencer; FPR: false positive rate; HGMD: Human Gene Mutation Database; HSF: Human Splice Finder; ISE: intronic splicing enhancer; ISS: intronic splicing silencer; MAF: minor allele frequency; MCC: Matthew’s correlation coefficient; NGS: next generation sequencing; NI: neighborhood inference; RF: Random Forest; ROC: receiver operating characteristic; SAV: splice-altering variant; SNP: single nucleotide polymorphism; SNV: splice neutral variant; SNP-SNV: single nucleotide polymorphism splice neutral variant; SVM: support vector machine; TS: tumor suppressor.

## Competing interests

The authors declare that they have no competing interests.

## Authors’ contributions

MM and SDM conceived the idea. MM and EVB compiled the datasets. MM designed the method with assistance from PR, SDM, TS-W, BL, JRS and DNC. MM implemented the method and performed the analyses. TS-W designed and implemented the ESR-HS feature. MM and BL evaluated existing third party tools. MM developed the website and stand-alone software package. JRS and TS-W conducted the experimental work. MM drafted and critically revised the manuscript. DNC, TS-W, MM, SDM, JRS and PR reviewed and edited the manuscript. All authors read and approved the final manuscript.

## Supplementary Material

Additional file 1: Table S1 1,189 putative SAVs derived from HGMD employed in this study. **Table S2.** unseen test set of 352 variants (238 SAVs and 114 SNVs) employed in this study. Click here for file

Additional file 2: Figure S1 experimental validation of exon skipping for a true positive MutPred Splice prediction (Mixed negative set, Iter. 3). The disease-causing mutation CM980147 (NM_000051.3: ATM c.5932G > T; NP_000042.3: p.E1978X), which is not present in any training data or the unseen evaluation test set, was predicted by MutPred Splice to disrupt splicing. **(A)** Schematic diagram of the exons assayed by RT-PCR. The mutation in exon 41 is indicated. **(B)** RT-PCR analysis of spliced mRNA isoforms from mutant or wild-type *ATM* genes. This experiment compares splicing of *ATM* pre-mRNA in patient-derived lymphoblastoid cells (E1978X) and HEK293 cells (wild type). Amplicons derived from different *ATM* mRNA isoforms were by resolved by 1% agarose gel electrophoresis. **Figure S2.** novel ESR hexamer score function (ESR-HS) to express the relationship between disease-causing and common putatively neutral variants and their differential distributions with respect to loss or gain of an ESE or ESS. Frequencies corresponding to disease-causing mutations (red) and common SNPs (blue) are shown. See Materials and methods for more details. Click here for file
