# Supplementary material for: MutPred Splice: machine learning-based prediction of exonic variants that disrupt splicing
Source: Genome Biol. 2014 Jan 13;15(1):R19. doi: 10.1186/gb-2014-15-1-r19 (PMC4054890; doi:10.1186/gb-2014-15-1-r19)
Supplement: Additional file 1: Table S1 — 1,189 putative SAVs derived from HGMD employed in this study. Table S2. unseen test set of 352 variants (238 SAVs and 114 SNVs) employed in this study. [file gb-2014-15-1-r19-S1.pdf]

Supplementary Table S1. 1,189 putative SAVs derived from HGMD employed in this study.

HGVS
NM_000018.2:c.1269G>A,NP_000009.1:p.S423S
NM_000019.3:c.1124A>G,NP_000010.1:p.N375S
NM_000019.3:c.814C>T,NP_000010.1:p.Q272X
NM_000019.3:c.951C>T,NP_000010.1:p.D317D
NM_000020.2:c.1246G>A,NP_000011.2:p.G416S
NM_000022.2:c.424C>T,NP_000013.2:p.R142X
NM_000026.2:c.618C>A,NP_000017.1:p.A206A
NM_000027.3:c.677G>A,NP_000018.2:p.G226D
NM_000029.3:c.1124G>A,NP_000020.1:p.R375Q
NM_000033.3:c.1224G>A,NP_000024.2:p.E408E
NM_000033.3:c.900G>A,NP_000024.2:p.E300E
NM_000035.3:c.324G>A,NP_000026.2:p.K108K
NM_000037.3:c.5005G>T,NP_000028.3:p.E1669X
NM_000038.5:c.1240C>T,NP_000029.2:p.R414C
NM_000038.5:c.1242C>T,NP_000029.2:p.R414R
NM_000038.5:c.1548G>A,NP_000029.2:p.K516K
NM_000038.5:c.1548G>C,NP_000029.2:p.K516N
NM_000038.5:c.1548G>T,NP_000029.2:p.K516N
NM_000038.5:c.1742A>G,NP_000029.2:p.K581R
NM_000038.5:c.1744G>T,NP_000029.2:p.E582X
NM_000038.5:c.1918C>G,NP_000029.2:p.R640G
NM_000038.5:c.1956C>T,NP_000029.2:p.H652H
NM_000038.5:c.1957A>C,NP_000029.2:p.R653R
NM_000038.5:c.1957A>G,NP_000029.2:p.R653G

NM_000038.5:c.1957A>T,NP_000029.2:p.R653W
NM_000038.5:c.1958G>A,NP_000029.2:p.R653K
NM_000038.5:c.1958G>C,NP_000029.2:p.R653T
NM_000038.5:c.1958G>T,NP_000029.2:p.R653M
NM_000038.5:c.423G>T,NP_000029.2:p.R141S
NM_000038.5:c.4909G>A,NP_000029.2:p.D1637N
NM_000038.5:c.834G>A,NP_000029.2:p.Q278Q
NM_000038.5:c.834G>C,NP_000029.2:p.Q278H
NM_000038.5:c.933G>C,NP_000029.2:p.K311N
NM_000043.4:c.341A>T,NP_000034.1:p.E114V
NM_000043.4:c.676G>A,NP_000034.1:p.D226N
NM_000044.2:c.2667C>T,NP_000035.2:p.S889S
NM_000046.3:c.312G>C,NP_000037.2:p.Q104H
NM_000049.2:c.432G>A,NP_000040.1:p.K144K
NM_000051.3:c.1235G>C,NP_000042.3:p.W412S
NM_000051.3:c.1802G>T,NP_000042.3:p.S601I
NM_000051.3:c.2250G>A,NP_000042.3:p.K750K
NM_000051.3:c.2376G>A,NP_000042.3:p.K792K
NM_000051.3:c.3284G>C,NP_000042.3:p.R1095T
NM_000051.3:c.3576G>A,NP_000042.3:p.K1192K
NM_000051.3:c.4777G>T,NP_000042.3:p.E1593X
NM_000051.3:c.487C>T,NP_000042.3:p.Q163X
NM_000051.3:c.513C>T,NP_000042.3:p.Y171Y
NM_000051.3:c.5319G>A,NP_000042.3:p.K1773K
NM_000051.3:c.5971G>T,NP_000042.3:p.E1991X
NM_000051.3:c.6095G>A,NP_000042.3:p.R2032K
NM_000051.3:c.662G>T,NP_000042.3:p.R221I

NM_000051.3:c.7449G>A,NP_000042.3:p.W2483X
NM_000051.3:c.748C>T,NP_000042.3:p.R250X
NM_000051.3:c.7788G>A,NP_000042.3:p.E2596E
NM_000051.3:c.7789G>T,NP_000042.3:p.D2597Y
NM_000051.3:c.7926A>C,NP_000042.3:p.R2642S
NM_000051.3:c.802C>T,NP_000042.3:p.Q268X
NM_000051.3:c.8150A>G,NP_000042.3:p.K2717R
NM_000051.3:c.8850G>T,NP_000042.3:p.E2950D
NM_000052.4:c.1910C>T,NP_000043.3:p.S637L
NM_000052.4:c.2059A>G,NP_000043.3:p.M687V
NM_000052.4:c.2172G>T,NP_000043.3:p.Q724H
NM_000052.4:c.2406G>C,NP_000043.3:p.K802N
NM_000052.4:c.2497A>G,NP_000043.3:p.S833G
NM_000052.4:c.2609A>G,NP_000043.3:p.D870G
NM_000052.4:c.2626G>C,NP_000043.3:p.G876R
NM_000052.4:c.2627G>A,NP_000043.3:p.G876E
NM_000052.4:c.3043G>A,NP_000043.3:p.G1015S
NM_000052.4:c.3111G>T,NP_000043.3:p.K1037N
NM_000053.2:c.3556G>A,NP_000044.2:p.G1186S
NM_000053.2:c.4021G>A,NP_000044.2:p.G1341S
NM_000059.3:c.145G>T,NP_000050.2:p.E49X
NM_000059.3:c.1763A>G,NP_000050.2:p.N588S
NM_000059.3:c.231T>G,NP_000050.2:p.T77T
NM_000059.3:c.425G>T,NP_000050.2:p.S142I
NM_000059.3:c.439C>T,NP_000050.2:p.Q147X
NM_000059.3:c.475G>A,NP_000050.2:p.V159M
NM_000059.3:c.516G>A,NP_000050.2:p.K172K

NM_000059.3:c.520C>T,NP_000050.2:p.R174C
NM_000059.3:c.631G>A,NP_000050.2:p.V211I
NM_000059.3:c.7805G>C,NP_000050.2:p.R2602T
NM_000059.3:c.7976G>A,NP_000050.2:p.R2659K
NM_000059.3:c.7992T>A,NP_000050.2:p.I2664I
NM_000059.3:c.8023A>G,NP_000050.2:p.I2675V
NM_000059.3:c.8378G>A,NP_000050.2:p.G2793E
NM_000059.3:c.8486A>G,NP_000050.2:p.Q2829R
NM_000059.3:c.8486A>T,NP_000050.2:p.Q2829L
NM_000059.3:c.8487G>A,NP_000050.2:p.Q2829Q
NM_000059.3:c.9006A>T,NP_000050.2:p.E3002D
NM_000059.3:c.9116C>T,NP_000050.2:p.P3039L
NM_000059.3:c.9117G>A,NP_000050.2:p.P3039P
NM_000059.3:c.9256G>T,NP_000050.2:p.G3086X
NM_000060.2:c.459G>A,NP_000051.1:p.E153E
NM_000061.2:c.1173A>G,NP_000052.1:p.G391G
NM_000061.2:c.1750G>A,NP_000052.1:p.G584R
NM_000061.2:c.1750G>T,NP_000052.1:p.G584W
NM_000061.2:c.1763G>A,NP_000052.1:p.W588X
NM_000061.2:c.240G>A,NP_000052.1:p.P80P
NM_000061.2:c.588G>C,NP_000052.1:p.Q196H
NM_000061.2:c.840G>T,NP_000052.1:p.E280D
NM_000062.2:c.550G>A,NP_000053.2:p.G184R
NM_000062.2:c.550G>C,NP_000053.2:p.G184R
NM_000062.2:c.882C>G,NP_000053.2:p.Y294X
NM_000062.2:c.884T>G,NP_000053.2:p.L295R
NM_000068.3:c.4012A>G,NP_000059.3:p.K1338E

NM_000068.3:c.575G>A,NP_000059.3:p.R192Q
NM_000068.3:c.653C>T,NP_000059.3:p.S218L
NM_000070.2:c.1525G>T,NP_000061.1:p.V509F
NM_000070.2:c.1872C>T,NP_000061.1:p.G624G
NM_000070.2:c.2184G>A,NP_000061.1:p.Q728Q
NM_000070.2:c.309G>A,NP_000061.1:p.P103P
NM_000070.2:c.633G>C,NP_000061.1:p.K211N
NM_000070.2:c.633G>T,NP_000061.1:p.K211N
NM_000071.2:c.313C>G,NP_000062.1:p.L105V
NM_000074.2:c.156G>A,NP_000065.1:p.K52K
NM_000074.2:c.346G>A,NP_000065.1:p.G116S
NM_000074.2:c.346G>C,NP_000065.1:p.G116R
NM_000077.4:c.149A>C,NP_000068.1:p.Q50P
NM_000077.4:c.457G>A,NP_000068.1:p.D153N
NM_000077.4:c.457G>T,NP_000068.1:p.D153Y
NM_000078.2:c.658G>A,NP_000069.2:p.A220T
NM_000080.3:c.915C>T,NP_000071.1:p.G305G
NM_000080.3:c.917G>T,NP_000071.1:p.R306M
NM_000082.3:c.479C>T,NP_000073.1:p.A160V
NM_000083.2:c.979G>A,NP_000074.2:p.V327I
NM_000084.2:c.1802A>T,NP_000075.1:p.D601V
NM_000088.3:c.543G>A,NP_000079.2:p.M181I
NM_000089.3:c.2188G>T,NP_000080.2:p.G730C
NM_000089.3:c.279G>A,NP_000080.2:p.M93I
NM_000090.3:c.2022G>A,NP_000081.1:p.K674K
NM_000090.3:c.2337G>A,NP_000081.1:p.K779K
NM_000091.4:c.1315G>A,NP_000082.2:p.G439S

NM_000092.4:c.1986A>G,NP_000083.3:p.K662K
NM_000094.3:c.2440G>C,NP_000085.1:p.G814R
NM_000094.3:c.341G>T,NP_000085.1:p.G114V
NM_000094.3:c.4011G>A,NP_000085.1:p.P1337P
NM_000094.3:c.4118C>T,NP_000085.1:p.S1373L
NM_000094.3:c.425A>G,NP_000085.1:p.K142R
NM_000094.3:c.4669G>C,NP_000085.1:p.G1557R
NM_000094.3:c.5097G>A,NP_000085.1:p.P1699P
NM_000094.3:c.520G>A,NP_000085.1:p.G174R
NM_000094.3:c.5499C>T,NP_000085.1:p.G1833G
NM_000094.3:c.5771A>C,NP_000085.1:p.Q1924P
NM_000094.3:c.5820G>A,NP_000085.1:p.P1940P
NM_000094.3:c.6180G>C,NP_000085.1:p.R2060S
NM_000094.3:c.6501G>A,NP_000085.1:p.P2167P
NM_000094.3:c.6619G>C,NP_000085.1:p.G2207R
NM_000094.3:c.6654C>G,NP_000085.1:p.G2218G
NM_000094.3:c.6846G>C,NP_000085.1:p.L2282L
NM_000094.3:c.6899A>G,NP_000085.1:p.Q2300R
NM_000094.3:c.6900G>A,NP_000085.1:p.Q2300Q
NM_000094.3:c.7017C>T,NP_000085.1:p.G2339G
NM_000094.3:c.7023G>A,NP_000085.1:p.K2341K
NM_000094.3:c.7191C>A,NP_000085.1:p.P2397P
NM_000094.3:c.7245G>A,NP_000085.1:p.M2415I
NM_000094.3:c.7344G>A,NP_000085.1:p.V2448V
NM_000094.3:c.8045A>G,NP_000085.1:p.K2682R
NM_000094.3:c.8439A>G,NP_000085.1:p.S2813S
NM_000094.3:c.846G>A,NP_000085.1:p.E282E

NM_000097.5:c.1277G>A,NP_000088.3:p.R426Q
NM_000100.2:c.168G>A,NP_000091.1:p.K56K
NM_000100.2:c.66G>A,NP_000091.1:p.Q22Q
NM_000101.2:c.288G>T,NP_000092.2:p.L96L
NM_000102.3:c.1263G>A,NP_000093.1:p.A421A
NM_000106.4:c.974C>T,NP_000097.2:p.P325L
NM_004006.2: c.4250T>A; NP_003997.1: p.L1417X
NM_004006.2:c.4303G>T;NP_003997.1:p.E1435X
NM_000116.3:c.238G>A,NP_000107.1:p.G80R
NM_000117.2:c.399G>T,NP_000108.1:p.Q133H
NM_000118.2:c.1134G>A,NP_000109.1:p.A378A
NM_000118.2:c.1311G>A,NP_000109.1:p.R437R
NM_000118.2:c.1311G>C,NP_000109.1:p.R437R
NM_000118.2:c.1311G>T,NP_000109.1:p.R437R
NM_000118.2:c.1428G>A,NP_000109.1:p.Q476Q
NM_000118.2:c.1428G>C,NP_000109.1:p.Q476H
NM_000118.2:c.219G>A,NP_000109.1:p.T73T
NM_000118.2:c.523G>A,NP_000109.1:p.A175T
NM_000118.2:c.523G>C,NP_000109.1:p.A175P
NM_000124.2:c.1913A>G,NP_000115.1:p.Y638C
NM_000124.2:c.2254A>G,NP_000115.1:p.M752V
NM_000127.2:c.1723G>C,NP_000118.2:p.V575L
NM_000128.3:c.1026G>T,NP_000119.1:p.G342G
NM_000128.3:c.325G>A,NP_000119.1:p.A109T
NM_000128.3:c.865G>C,NP_000119.1:p.V289L
NM_000129.3:c.1111T>G,NP_000120.2:p.W371G
NM_000129.3:c.2045G>A,NP_000120.2:p.R682H

NM_000129.3:c.319G>T,NP_000120.2:p.G107C
NM_000130.4:c.1611G>T,NP_000121.2:p.Q537H
NM_000130.4:c.1976G>A,NP_000121.2:p.G659E
NM_000130.4:c.5419G>A,NP_000121.2:p.A1807T
NM_000131.3:c.291G>C,NP_000122.1:p.T97T
NM_000131.3:c.316G>A,NP_000122.1:p.D106N
NM_000132.3:c.1009G>T,NP_000123.1:p.D337Y
NM_000132.3:c.5217C>T,NP_000123.1:p.N1739N
NM_000132.3:c.5540A>G,NP_000123.1:p.D1847G
NM_000132.3:c.5586G>A,NP_000123.1:p.L1862L
NM_000132.3:c.5999G>A,NP_000123.1:p.G2000D
NM_000132.3:c.601G>C,NP_000123.1:p.G201R
NM_000132.3:c.669A>G,NP_000123.1:p.E223E
NM_000132.3:c.670G>T,NP_000123.1:p.G224W
NM_000132.3:c.6724G>A,NP_000123.1:p.V2242M
NM_000132.3:c.787G>C,NP_000123.1:p.G263R
NM_000133.3:c.153A>G,NP_000124.1:p.K51K
NM_000133.3:c.277G>A,NP_000124.1:p.D93N
NM_000133.3:c.459G>A,NP_000124.1:p.V153V
NM_000133.3:c.484C>A,NP_000124.1:p.R162R
NM_000133.3:c.519A>C,NP_000124.1:p.A173A
NM_000133.3:c.519A>G,NP_000124.1:p.A173A
NM_000133.3:c.519A>T,NP_000124.1:p.A173A
NM_000133.3:c.520G>T,NP_000124.1:p.V174L
NM_000133.3:c.711A>G,NP_000124.1:p.Q237Q
NM_000133.3:c.723G>A,NP_000124.1:p.Q241Q
NM_000133.3:c.87A>G,NP_000124.1:p.T29T

NM_000133.3:c.88G>C,NP_000124.1:p.V30L
NM_000135.2:c.1470G>A,NP_000126.2:p.Q490Q
NM_000135.2:c.2632G>C,NP_000126.2:p.E878Q
NM_000135.2:c.3624C>T,NP_000126.2:p.S1208S
NM_000136.2:c.520C>T,NP_000127.2:p.R174X
NM_000136.2:c.996G>A,NP_000127.2:p.Q332Q
NM_000137.2:c.1061C>A,NP_000128.1:p.P354Q
NM_000137.2:c.192G>T,NP_000128.1:p.Q64H
NM_000137.2:c.836A>G,NP_000128.1:p.Q279R
NM_000138.4:c.1147G>A,NP_000129.3:p.E383K
NM_000138.4:c.1468G>T,NP_000129.3:p.D490Y
NM_000138.4:c.1588G>A,NP_000129.3:p.D530N
NM_000138.4:c.164G>C,NP_000129.3:p.G55A
NM_000138.4:c.2113G>A,NP_000129.3:p.A705T
NM_000138.4:c.2167G>A,NP_000129.3:p.D723N
NM_000138.4:c.2677G>A,NP_000129.3:p.D893N
NM_000138.4:c.2728G>A,NP_000129.3:p.D910N
NM_000138.4:c.2728G>T,NP_000129.3:p.D910Y
NM_000138.4:c.2854G>C,NP_000129.3:p.D952H
NM_000138.4:c.3208G>C,NP_000129.3:p.D1070H
NM_000138.4:c.3294C>T,NP_000129.3:p.D1098D
NM_000138.4:c.3463G>A,NP_000129.3:p.D1155N
NM_000138.4:c.3463G>C,NP_000129.3:p.D1155H
NM_000138.4:c.3589G>C,NP_000129.3:p.D1197H
NM_000138.4:c.3963A>G,NP_000129.3:p.T1321T
NM_000138.4:c.3964G>A,NP_000129.3:p.D1322N
NM_000138.4:c.4459G>A,NP_000129.3:p.D1487N

NM_000138.4:c.5343G>A,NP_000129.3:p.V1781V
NM_000138.4:c.538G>C,NP_000129.3:p.D180H
NM_000138.4:c.5816G>A,NP_000129.3:p.G1939E
NM_000138.4:c.6496G>A,NP_000129.3:p.D2166N
NM_000138.4:c.6871G>C,NP_000129.3:p.D2291H
NM_000140.3:c.1077G>A,NP_000131.2:p.E359E
NM_000140.3:c.1085T>G,NP_000131.2:p.V362G
NM_000140.3:c.1135A>T,NP_000131.2:p.K379X
NM_000140.3:c.463G>C,NP_000131.2:p.A155P
NM_000140.3:c.913G>T,NP_000131.2:p.V305F
NM_000141.4:c.1032G>A,NP_000132.3:p.A344A
NM_000141.4:c.940G>T,NP_000132.3:p.A314S
NM_000151.2:c.563G>A,NP_000142.1:p.G188D
NM_000151.2:c.648G>T,NP_000142.1:p.L216L
NM_000152.3:c.1437G>A,NP_000143.2:p.K479K
NM_000152.3:c.1626C>G,NP_000143.2:p.P542P
NM_000152.3:c.2040G>A,NP_000143.2:p.L680L
NM_000152.3:c.546G>A,NP_000143.2:p.T182T
NM_000152.3:c.546G>C,NP_000143.2:p.T182T
NM_000152.3:c.546G>T,NP_000143.2:p.T182T
NM_000153.3:c.195G>C,NP_000144.2:p.G65G
NM_000155.2:c.290A>G,NP_000146.2:p.N97S
NM_000156.4:c.327G>A,NP_000147.1:p.K109K
NM_000161.2:c.557C>A,NP_000152.1:p.T186K
NM_000162.3:c.207A>G,NP_000153.1:p.S69S
NM_000162.3:c.459T>G,NP_000153.1:p.P153P
NM_000162.3:c.45G>A,NP_000153.1:p.K15K

NM_000162.3:c.483G>A,NP_000153.1:p.K161K
NM_000162.3:c.579G>T,NP_000153.1:p.G193G
NM_000163.4:c.875G>C,NP_000154.1:p.R292T
NM_000169.2:c.194G>C,NP_000160.1:p.S65T
NM_000169.2:c.639G>T,NP_000160.1:p.K213N
NM_000170.2:c.2607C>A,NP_000161.2:p.P869P
NM_000178.2:c.1034A>C,NP_000169.1:p.E345A
NM_000178.2:c.1111G>T,NP_000169.1:p.G371C
NM_000178.2:c.491G>A,NP_000169.1:p.R164Q
NM_000178.2:c.799C>G,NP_000169.1:p.R267G
NM_000179.2:c.3991C>T,NP_000170.1:p.R1331X
NM_000179.2:c.4001G>A,NP_000170.1:p.R1334Q
NM_000190.3:c.210G>A,NP_000181.2:p.K70K
NM_000190.3:c.33G>T,NP_000181.2:p.A11A
NM_000190.3:c.612G>T,NP_000181.2:p.Q204H
NM_000190.3:c.770T>C,NP_000181.2:p.L257P
NM_000190.3:c.771G>A,NP_000181.2:p.L257L
NM_000190.3:c.771G>C,NP_000181.2:p.L257L
NM_000190.3:c.86A>T,NP_000181.2:p.Q29L
NM_000190.3:c.88C>A,NP_000181.2:p.L30I
NM_000191.2:c.434A>T,NP_000182.2:p.E145V
NM_000194.2:c.130G>T,NP_000185.1:p.D44Y
NM_000194.2:c.325C>T,NP_000185.1:p.Q109X
NM_000194.2:c.430C>T,NP_000185.1:p.Q144X
NM_000195.3:c.507G>A,NP_000186.2:p.E169E
NM_000195.3:c.937G>A,NP_000186.2:p.G313S
NM_000202.5:c.1006G>A,NP_000193.1:p.G336R

NM_000202.5:c.1006G>C,NP_000193.1:p.G336R
NM_000202.5:c.245C>T,NP_000193.1:p.A82V
NM_000202.5:c.257C>T,NP_000193.1:p.P86L
NM_000202.5:c.418G>A,NP_000193.1:p.G140R
NM_000202.5:c.419G>T,NP_000193.1:p.G140V
NM_000202.5:c.542A>T,NP_000193.1:p.N181I
NM_000202.5:c.641C>G,NP_000193.1:p.T214R
NM_000202.5:c.708G>A,NP_000193.1:p.K236K
NM_000202.5:c.879G>A,NP_000193.1:p.Q293Q
NM_000202.5:c.879G>C,NP_000193.1:p.Q293H
NM_000202.5:c.998C>T,NP_000193.1:p.S333L
NM_000203.3:c.1113C>A,NP_000194.2:p.V371V
NM_000203.3:c.590G>A,NP_000194.2:p.G197D
NM_000204.3:c.772G>A,NP_000195.2:p.A258T
NM_000206.2:c.115G>A,NP_000197.1:p.D39N
NM_000206.2:c.854G>A,NP_000197.1:p.R285Q
NM_000206.2:c.924G>C,NP_000197.1:p.S308S
NM_000208.2:c.3258G>A,NP_000199.2:p.V1086V
NM_000212.2:c.1260G>A,NP_000203.2:p.T420T
NM_000214.2:c.1395G>T,NP_000205.1:p.R465R
NM_000214.2:c.755G>A,NP_000205.1:p.R252K
NM_000215.3:c.2961C>T,NP_000206.2:p.G987G
NM_000218.2:c.1032G>A,NP_000209.2:p.A344A
NM_000218.2:c.1032G>C,NP_000209.2:p.A344A
NM_000218.2:c.1032G>T,NP_000209.2:p.A344A
NM_000218.2:c.1794G>A,NP_000209.2:p.K598K
NM_000218.2:c.921G>A,NP_000209.2:p.V307V

NM_000228.2:c.1976G>A,NP_000219.2:p.R659K
NM_000228.2:c.3009C>T,NP_000219.2:p.G1003G
NM_000228.2:c.628G>A,NP_000219.2:p.E210K
NM_000230.2:c.144G>A,NP_000221.1:p.T48T
NM_000235.2:c.892C>T,NP_000226.2:p.Q298X
NM_000235.2:c.894G>A,NP_000226.2:p.Q298Q
NM_000238.3:c.1128G>A,NP_000229.1:p.Q376Q
NM_000238.3:c.2145G>A,NP_000229.1:p.A715A
NM_000238.3:c.2398G>A,NP_000229.1:p.G800R
NM_000248.3:c.909G>A,NP_000239.1:p.T303T
NM_000249.3:c.1037A>G,NP_000240.1:p.Q346R
NM_000249.3:c.1038G>A,NP_000240.1:p.Q346Q
NM_000249.3:c.1038G>C,NP_000240.1:p.Q346H
NM_000249.3:c.1038G>T,NP_000240.1:p.Q346H
NM_000249.3:c.116G>A,NP_000240.1:p.C39Y
NM_000249.3:c.116G>T,NP_000240.1:p.C39F
NM_000249.3:c.122A>G,NP_000240.1:p.D41G
NM_000249.3:c.1381A>T,NP_000240.1:p.K461X
NM_000249.3:c.1667G>T,NP_000240.1:p.S556I
NM_000249.3:c.1731G>A,NP_000240.1:p.S577S
NM_000249.3:c.1731G>C,NP_000240.1:p.S577S
NM_000249.3:c.1896G>A,NP_000240.1:p.E632E
NM_000249.3:c.1896G>C,NP_000240.1:p.E632D
NM_000249.3:c.1896G>T,NP_000240.1:p.E632D
NM_000249.3:c.1958T>G,NP_000240.1:p.L653R
NM_000249.3:c.1963A>G,NP_000240.1:p.I655V
NM_000249.3:c.1975C>T,NP_000240.1:p.R659X

NM_000249.3:c.1988A>G,NP_000240.1:p.E663G
NM_000249.3:c.1989G>A,NP_000240.1:p.E663E
NM_000249.3:c.1989G>T,NP_000240.1:p.E663D
NM_000249.3:c.2103G>C,NP_000240.1:p.Q701H
NM_000249.3:c.304G>A,NP_000240.1:p.E102K
NM_000249.3:c.380G>A,NP_000240.1:p.R127K
NM_000249.3:c.382G>A,NP_000240.1:p.A128T
NM_000249.3:c.544A>G,NP_000240.1:p.R182G
NM_000249.3:c.677G>A,NP_000240.1:p.R226Q
NM_000249.3:c.677G>T,NP_000240.1:p.R226L
NM_000249.3:c.67G>T,NP_000240.1:p.E23X
NM_000249.3:c.717C>A,NP_000240.1:p.A239A
NM_000249.3:c.731G>A,NP_000240.1:p.G244D
NM_000249.3:c.793C>T,NP_000240.1:p.R265C
NM_000249.3:c.842C>T,NP_000240.1:p.A281V
NM_000249.3:c.86C>G,NP_000240.1:p.A29G
NM_000249.3:c.882C>T,NP_000240.1:p.L294L
NM_000249.3:c.883A>C,NP_000240.1:p.S295R
NM_000249.3:c.883A>G,NP_000240.1:p.S295G
NM_000249.3:c.884G>A,NP_000240.1:p.S295N
NM_000251.1:c.1075A>G,NP_000242.1:p.R359G
NM_000251.1:c.1275A>G,NP_000242.1:p.E425E
NM_000251.1:c.1276G>C,NP_000242.1:p.G426R
NM_000251.1:c.1510G>C,NP_000242.1:p.G504R
NM_000251.1:c.1516G>T,NP_000242.1:p.D506Y
NM_000251.1:c.1600C>T,NP_000242.1:p.R534C
NM_000251.1:c.1660A>G,NP_000242.1:p.S554G

NM_000251.1:c.1660A>T,NP_000242.1:p.S554C
NM_000251.1:c.1661G>A,NP_000242.1:p.S554N
NM_000251.1:c.1661G>C,NP_000242.1:p.S554T
NM_000251.1:c.1759G>C,NP_000242.1:p.G587R
NM_000251.1:c.1915C>T,NP_000242.1:p.H639Y
NM_000251.1:c.1979A>G,NP_000242.1:p.D660G
NM_000251.1:c.2634G>A,NP_000242.1:p.E878E
NM_000251.1:c.2634G>C,NP_000242.1:p.E878D
NM_000251.1:c.644A>G,NP_000242.1:p.Q215R
NM_000251.1:c.806C>T,NP_000242.1:p.S269L
NM_000251.1:c.815C>T,NP_000242.1:p.A272V
NM_000251.1:c.942G>A,NP_000242.1:p.Q314Q
NM_000252.2:c.1234A>G,NP_000243.1:p.I412V
NM_000252.2:c.417A>G,NP_000243.1:p.R139R
NM_000252.2:c.444G>C,NP_000243.1:p.L148L
NM_000253.2:c.1989G>A,NP_000244.2:p.Q663Q
NM_000253.2:c.619G>T,NP_000244.2:p.V207F
NM_000255.3:c.1560G>C,NP_000246.2:p.K520N
NM_000255.3:c.1808G>A,NP_000246.2:p.R603K
NM_000256.3:c.772G>A,NP_000247.2:p.E258K
NM_000260.3:c.1200G>T,NP_000251.3:p.K400N
NM_000260.3:c.1690G>A,NP_000251.3:p.G564S
NM_000260.3:c.1935G>A,NP_000251.3:p.M645I
NM_000260.3:c.3503G>C,NP_000251.3:p.R1168P
NM_000260.3:c.5856G>A,NP_000251.3:p.K1952K
NM_000260.3:c.592G>A,NP_000251.3:p.A198T
NM_000260.3:c.5944G>A,NP_000251.3:p.G1982R

NM_000264.3:c.1503G>A,NP_000255.2:p.Q501Q
NM_000264.3:c.584G>A,NP_000255.2:p.R195K
NM_000265.4:c.574G>A,NP_000256.3:p.G192S
NM_000267.3:c.1007G>A,NP_000258.1:p.W336X
NM_000267.3:c.1039C>T,NP_000258.1:p.Q347X
NM_000267.3:c.1062G>A,NP_000258.1:p.K354K
NM_000267.3:c.1098A>T,NP_000258.1:p.R366S
NM_000267.3:c.1185G>C,NP_000258.1:p.K395N
NM_000267.3:c.1235A>G,NP_000258.1:p.N412S
NM_000267.3:c.1513A>G,NP_000258.1:p.K505E
NM_000267.3:c.1658A>G,NP_000258.1:p.H553R
NM_000267.3:c.1700T>G,NP_000258.1:p.V567G
NM_000267.3:c.1721G>A,NP_000258.1:p.S574N
NM_000267.3:c.1721G>C,NP_000258.1:p.S574T
NM_000267.3:c.1722C>A,NP_000258.1:p.S574R
NM_000267.3:c.1748A>G,NP_000258.1:p.K583R
NM_000267.3:c.1845G>T,NP_000258.1:p.K615N
NM_000267.3:c.2040C>T,NP_000258.1:p.C680C
NM_000267.3:c.2251G>A,NP_000258.1:p.G751R
NM_000267.3:c.2256A>G,NP_000258.1:p.R752R
NM_000267.3:c.2325G>T,NP_000258.1:p.E775D
NM_000267.3:c.288G>T,NP_000258.1:p.G96G
NM_000267.3:c.2989A>G,NP_000258.1:p.R997G
NM_000267.3:c.3113G>C,NP_000258.1:p.R1038T
NM_000267.3:c.3277G>A,NP_000258.1:p.V1093M
NM_000267.3:c.3427C>T,NP_000258.1:p.H1143Y
NM_000267.3:c.3974G>A,NP_000258.1:p.R1325K

NM_000267.3:c.4268A>G,NP_000258.1:p.K1423R
NM_000267.3:c.4269G>C,NP_000258.1:p.K1423N
NM_000267.3:c.4435A>G,NP_000258.1:p.S1479G
NM_000267.3:c.479G>C,NP_000258.1:p.R160T
NM_000267.3:c.47G>C,NP_000258.1:p.R16P
NM_000267.3:c.5172G>A,NP_000258.1:p.K1724K
NM_000267.3:c.5224C>T,NP_000258.1:p.Q1742X
NM_000267.3:c.5234C>G,NP_000258.1:p.S1745X
NM_000267.3:c.5242C>T,NP_000258.1:p.R1748X
NM_000267.3:c.5264C>G,NP_000258.1:p.S1755X
NM_000267.3:c.5290G>T,NP_000258.1:p.A1764S
NM_000267.3:c.5294C>A,NP_000258.1:p.S1765X
NM_000267.3:c.5426G>T,NP_000258.1:p.R1809L
NM_000267.3:c.5427C>T,NP_000258.1:p.R1809R
NM_000267.3:c.5546G>A,NP_000258.1:p.R1849Q
NM_000267.3:c.5719G>T,NP_000258.1:p.E1907X
NM_000267.3:c.586G>T,NP_000258.1:p.E196X
NM_000267.3:c.58C>T,NP_000258.1:p.Q20X
NM_000267.3:c.5941C>T,NP_000258.1:p.Q1981X
NM_000267.3:c.5943G>A,NP_000258.1:p.Q1981Q
NM_000267.3:c.6724C>T,NP_000258.1:p.Q2242X
NM_000267.3:c.6858G>C,NP_000258.1:p.K2286N
NM_000267.3:c.7258G>C,NP_000258.1:p.A2420P
NM_000267.3:c.730G>A,NP_000258.1:p.E244K
NM_000267.3:c.7606C>T,NP_000258.1:p.Q2536X
NM_000267.3:c.8428A>T,NP_000258.1:p.K2810X
NM_000267.3:c.846G>A,NP_000258.1:p.Q282Q

NM_000267.3:c.910C>T,NP_000258.1:p.R304X
NM_000267.3:c.943C>T,NP_000258.1:p.Q315X
NM_000268.3:c.113A>T,NP_000259.1:p.E38V
NM_000268.3:c.1340G>C,NP_000259.1:p.R447T
NM_000268.3:c.1736A>G,NP_000259.1:p.K579R
NM_000268.3:c.1737G>T,NP_000259.1:p.K579N
NM_000268.3:c.447G>A,NP_000259.1:p.K149K
NM_000268.3:c.809A>G,NP_000259.1:p.E270G
NM_000268.3:c.810G>A,NP_000259.1:p.E270E
NM_000270.3:c.181G>T,NP_000261.2:p.V61L
NM_000271.4:c.1553G>A,NP_000262.2:p.R518Q
NM_000271.4:c.2292G>A,NP_000262.2:p.A764A
NM_000271.4:c.2599C>T,NP_000262.2:p.P867S
NM_000271.4:c.2911G>C,NP_000262.2:p.V971L
NM_000271.4:c.3422T>G,NP_000262.2:p.V1141G
NM_000271.4:c.3754G>C,NP_000262.2:p.G1252R
NM_000272.3:c.1027G>A,NP_000263.2:p.G343R
NM_000273.2:c.360G>A,NP_000264.2:p.A120A
NM_000273.2:c.455G>C,NP_000264.2:p.S152T
NM_000276.3:c.1466G>A,NP_000267.2:p.S489N
NM_000276.3:c.2581G>A,NP_000267.2:p.A861T
NM_000276.3:c.824G>C,NP_000267.2:p.G275A
NM_000277.1:c.1144T>C,NP_000268.1:p.F382L
NM_000277.1:c.1194A>G,NP_000268.1:p.K398K
NM_000277.1:c.1197A>T,NP_000268.1:p.V399V
NM_000277.1:c.1198A>C,NP_000268.1:p.R400R
NM_000277.1:c.30C>G,NP_000268.1:p.G10G

NM_000277.1:c.611A>G,NP_000268.1:p.Y204C
NM_000277.1:c.943G>T,NP_000268.1:p.D315Y
NM_000280.3:c.1031A>G,NP_000271.1:p.Q344R
NM_000280.3:c.1183G>C,NP_000271.1:p.G395R
NM_000280.3:c.765G>C,NP_000271.1:p.Q255H
NM_000280.3:c.765G>T,NP_000271.1:p.Q255H
NM_000280.3:c.94C>G,NP_000271.1:p.L32V
NM_000282.3:c.1430G>T,NP_000273.2:p.G477V
NM_000282.3:c.1746G>A,NP_000273.2:p.S582S
NM_000284.3:c.729C>A,NP_000275.1:p.Y243X
NM_000287.3:c.1054C>T,NP_000278.3:p.Q352X
NM_000287.3:c.2362G>A,NP_000278.3:p.V788M
NM_000289.5:c.1127G>A,NP_000280.1:p.R376Q
NM_000291.3:c.755A>C,NP_000282.1:p.E252A
NM_000292.2:c.2675A>G,NP_000283.1:p.Q892R
NM_000297.3:c.1320G>T,NP_000288.1:p.R440S
NM_000297.3:c.2657A>G,NP_000288.1:p.D886G
NM_000298.5:c.1269G>A,NP_000289.1:p.A423A
NM_000298.5:c.1269G>C,NP_000289.1:p.A423A
NM_000298.5:c.1618G>C,NP_000289.1:p.G540R
NM_000298.5:c.507G>A,NP_000289.1:p.G169G
NM_000298.5:c.694G>T,NP_000289.1:p.G232C
NM_000302.3:c.1095C>T,NP_000293.2:p.G365G
NM_000303.2:c.255G>A,NP_000294.1:p.Q85Q
NM_000309.3:c.338G>C,NP_000300.1:p.R113T
NM_000309.3:c.470A>C,NP_000300.1:p.E157A
NM_000309.3:c.471G>A,NP_000300.1:p.E157E

NM_000309.3:c.807G>A,NP_000300.1:p.K269K
NM_000309.3:c.868G>C,NP_000300.1:p.V290L
NM_000310.3:c.533A>T,NP_000301.1:p.E178V
NM_000312.3:c.678G>A,NP_000303.1:p.Q226Q
NM_000312.3:c.678G>C,NP_000303.1:p.Q226H
NM_000313.3:c.1155G>A,NP_000304.2:p.M385I
NM_000313.3:c.259G>C,NP_000304.2:p.V87L
NM_000314.4:c.209T>C,NP_000305.3:p.L70P
NM_000314.4:c.511C>T,NP_000305.3:p.Q171X
NM_000316.2:c.1148G>A,NP_000307.1:p.R383Q
NM_000317.2:c.168G>A,NP_000308.1:p.V56V
NM_000317.2:c.243G>A,NP_000308.1:p.E81E
NM_000321.2:c.1206C>T,NP_000312.2:p.S402S
NM_000321.2:c.1331A>G,NP_000312.2:p.Q444R
NM_000321.2:c.1332G>A,NP_000312.2:p.Q444Q
NM_000321.2:c.1332G>C,NP_000312.2:p.Q444H
NM_000321.2:c.1421G>A,NP_000312.2:p.S474N
NM_000321.2:c.1498A>C,NP_000312.2:p.R500R
NM_000321.2:c.1499G>T,NP_000312.2:p.R500I
NM_000321.2:c.1960G>A,NP_000312.2:p.V654M
NM_000321.2:c.1960G>C,NP_000312.2:p.V654L
NM_000321.2:c.1960G>T,NP_000312.2:p.V654L
NM_000321.2:c.2211G>A,NP_000312.2:p.E737E
NM_000321.2:c.2211G>C,NP_000312.2:p.E737D
NM_000321.2:c.264G>A,NP_000312.2:p.L88L
NM_000321.2:c.658C>G,NP_000312.2:p.L220V
NM_000321.2:c.861G>C,NP_000312.2:p.E287D

NM_000321.2:c.938A>G,NP_000312.2:p.E313G
NM_000321.2:c.939G>A,NP_000312.2:p.E313E
NM_000321.2:c.939G>T,NP_000312.2:p.E313D
NM_000324.2:c.1139G>T,NP_000315.2:p.G380V
NM_000326.4:c.141G>A,NP_000317.1:p.K47K
NM_000328.2:c.154G>A,NP_000319.1:p.G52R
NM_000329.2:c.95G>T,NP_000320.1:p.G32V
NM_000330.3:c.52G>A,NP_000321.1:p.A18T
NM_000339.2:c.1925G>A,NP_000330.2:p.R642H
NM_000341.3:c.1011G>A,NP_000332.2:p.P337P
NM_000341.3:c.1035G>A,NP_000332.2:p.E345E
NM_000347.5:c.300G>C,NP_000338.3:p.L100L
NM_000347.5:c.566G>C,NP_000338.3:p.G189A
NM_000349.2:c.650G>C,NP_000340.2:p.R217T
NM_000350.2:c.2588G>C,NP_000341.2:p.G863A
NM_000350.2:c.4849G>A,NP_000341.2:p.V1617M
NM_000350.2:c.768G>T,NP_000341.2:p.V256V
NM_000352.3:c.1176G>A,NP_000343.2:p.Q392Q
NM_000352.3:c.4307G>A,NP_000343.2:p.R1436Q
NM_000353.2:c.1224G>T,NP_000344.1:p.T408T
NM_000370.3:c.306A>G,NP_000361.1:p.G102G
NM_000370.3:c.552G>A,NP_000361.1:p.T184T
NM_000374.4:c.756C>G,NP_000365.3:p.G252G
NM_000374.4:c.942G>A,NP_000365.3:p.E314E
NM_000375.2:c.243A>T,NP_000366.1:p.E81D
NM_000375.2:c.244G>T,NP_000366.1:p.V82F
NM_000377.2:c.1453G>A,NP_000368.1:p.D485N

NM_000377.2:c.505G>T,NP_000368.1:p.E169X
NM_000377.2:c.671A>G,NP_000368.1:p.D224G
NM_000377.2:c.687G>T,NP_000368.1:p.G229G
NM_000377.2:c.919A>G,NP_000368.1:p.M307V
NM_000380.3:c.389G>A,NP_000371.1:p.R130K
NM_000380.3:c.555G>C,NP_000371.1:p.Q185H
NM_000380.3:c.673G>C,NP_000371.1:p.E225Q
NM_000382.2:c.798G>C,NP_000373.1:p.K266N
NM_000383.2:c.462A>T,NP_000374.1:p.P154P
NM_000383.2:c.463G>A,NP_000374.1:p.G155S
NM_000384.2:c.1124G>A,NP_000375.2:p.S375N
NM_000390.2:c.1412A>T,NP_000381.1:p.Q471L
NM_000391.3:c.225A>G,NP_000382.3:p.Q75Q
NM_000396.3:c.890G>A,NP_000387.1:p.S297N
NM_000397.3:c.252G>A,NP_000388.2:p.A84A
NM_000397.3:c.252G>T,NP_000388.2:p.A84A
NM_000397.3:c.483G>T,NP_000388.2:p.K161N
NM_000397.3:c.621C>A,NP_000388.2:p.Y207X
NM_000397.3:c.897G>A,NP_000388.2:p.K299K
NM_000400.3:c.2150C>G,NP_000391.1:p.A717G
NM_000404.2:c.246G>T,NP_000395.2:p.T82T
NM_000404.2:c.902C>T,NP_000395.2:p.A301V
NM_000419.3:c.1750C>T,NP_000410.2:p.R584X
NM_000419.3:c.1878G>C,NP_000410.2:p.Q626H
NM_000419.3:c.188G>A,NP_000410.2:p.R63K
NM_000419.3:c.3060G>A,NP_000410.2:p.K1020K
NM_000419.3:c.3062T>C,NP_000410.2:p.V1021A

NM_000419.3:c.399C>G,NP_000410.2:p.D133E
NM_000419.3:c.480C>G,NP_000410.2:p.S160R
NM_000421.3:c.1370G>T,NP_000412.3:p.G457V
NM_000425.3:c.645C>T,NP_000416.1:p.G215G
NM_000425.3:c.924C>T,NP_000416.1:p.G308G
NM_000426.3:c.2230C>T,NP_000417.2:p.R744X
NM_000426.3:c.470C>T,NP_000417.2:p.S157F
NM_000426.3:c.5530C>A,NP_000417.2:p.R1844S
NM_000429.2:c.292G>A,NP_000420.1:p.G98S
NM_000433.3:c.1026G>A,NP_000424.2:p.K342K
NM_000434.3:c.1022G>A,NP_000425.1:p.R341Q
NM_000436.3:c.671G>A,NP_000427.1:p.R224K
NM_000441.1:c.1001G>T,NP_000432.1:p.G334V
NM_000443.3:c.79A>G,NP_000434.1:p.S27G
NM_000444.4:c.1645C>T,NP_000435.3:p.R549X
NM_000444.4:c.591A>G,NP_000435.3:p.Q197Q
NM_000445.3:c.1344G>A,NP_000436.2:p.S448S
NM_000455.4:c.1041G>A,NP_000446.1:p.A347A
NM_000465.2:c.1977A>G,NP_000456.2:p.R659R
NM_000466.2:c.2364G>A,NP_000457.1:p.V788V
NM_000477.5:c.1427A>G,NP_000468.1:p.Y476C
NM_000477.5:c.597T>A,NP_000468.1:p.A199A
NM_000478.4:c.791A>G,NP_000469.3:p.K264R
NM_000487.5:c.1150G>A,NP_000478.3:p.E384K
NM_000487.5:c.1232C>T,NP_000478.3:p.T411I
NM_000488.3:c.1152A>G,NP_000479.1:p.P384P
NM_000488.3:c.1153G>A,NP_000479.1:p.G385S

NM_000488.3:c.624G>A,NP_000479.1:p.K208K
NM_000488.3:c.624G>T,NP_000479.1:p.K208N
NM_000488.3:c.626A>G,NP_000479.1:p.E209G
NM_000489.3:c.370G>T,NP_000480.2:p.G124C
NM_000489.3:c.4317G>A,NP_000480.2:p.K1439K
NM_000489.3:c.536A>G,NP_000480.2:p.N179S
NM_000489.3:c.5721G>A,NP_000480.2:p.M1907I
NM_000489.3:c.6003G>A,NP_000480.2:p.W2001X
NM_000492.3:c.1209G>A,NP_000483.3:p.E403E
NM_000492.3:c.1408G>A,NP_000483.3:p.V470M
NM_000492.3:c.1584G>A,NP_000483.3:p.E528E
NM_000492.3:c.1657C>T,NP_000483.3:p.R553X
NM_000492.3:c.178G>T,NP_000483.3:p.E60X
NM_000492.3:c.223C>T,NP_000483.3:p.R75X
NM_000492.3:c.2491G>T,NP_000483.3:p.E831X
NM_000492.3:c.254G>A,NP_000483.3:p.G85E
NM_000492.3:c.2679G>T,NP_000483.3:p.G893G
NM_000492.3:c.2988G>A,NP_000483.3:p.Q996Q
NM_000492.3:c.3468G>A,NP_000483.3:p.L1156L
NM_000492.3:c.3594G>T,NP_000483.3:p.V1198V
NM_000492.3:c.3717G>A,NP_000483.3:p.R1239R
NM_000492.3:c.3846G>A,NP_000483.3:p.W1282X
NM_000492.3:c.3873G>C,NP_000483.3:p.Q1291H
NM_000492.3:c.4389G>A,NP_000483.3:p.Q1463Q
NM_000492.3:c.489G>A,NP_000483.3:p.K163K
NM_000492.3:c.915C>T,NP_000483.3:p.F305F
NM_000495.3:c.1856C>T,NP_000486.1:p.P619L

NM_000495.3:c.2146G>C,NP_000486.1:p.G716R
NM_000495.3:c.2394A>G,NP_000486.1:p.K798K
NM_000495.3:c.2678G>T,NP_000486.1:p.G893V
NM_000495.3:c.2746A>G,NP_000486.1:p.S916G
NM_000495.3:c.385G>A,NP_000486.1:p.G129R
NM_000495.3:c.4688G>A,NP_000486.1:p.R1563Q
NM_000495.3:c.4766G>T,NP_000486.1:p.G1589V
NM_000495.3:c.4803G>A,NP_000486.1:p.M1601I
NM_000495.3:c.4803G>C,NP_000486.1:p.M1601I
NM_000495.3:c.4976G>A,NP_000486.1:p.S1659N
NM_000496.2:c.54G>A,NP_000487.1:p.K18K
NM_000497.3:c.799G>A,NP_000488.3:p.G267S
NM_000497.3:c.799G>C,NP_000488.3:p.G267R
NM_000497.3:c.800G>A,NP_000488.3:p.G267D
NM_000497.3:c.954G>A,NP_000488.3:p.T318T
NM_000497.3:c.954G>C,NP_000488.3:p.T318T
NM_000503.4:c.1051G>T,NP_000494.2:p.D351Y
NM_000515.3:c.172G>A,NP_000506.2:p.E58K
NM_000515.3:c.172G>T,NP_000506.2:p.E58X
NM_000515.3:c.173A>C,NP_000506.2:p.E58A
NM_000515.3:c.456G>A,NP_000506.2:p.G152G
NM_000517.4:c.96G>A,NP_000508.1:p.R32R
NM_000518.4:c.33C>A,NP_000509.1:p.A11A
NM_000518.4:c.51C>T,NP_000509.1:p.G17G
NM_000518.4:c.82G>T,NP_000509.1:p.A28S
NM_000518.4:c.90C>T,NP_000509.1:p.G30G
NM_000518.4:c.91A>C,NP_000509.1:p.R31R

NM_000518.4:c.91A>G,NP_000509.1:p.R31G
NM_000518.4:c.92G>A,NP_000509.1:p.R31K
NM_000518.4:c.92G>C,NP_000509.1:p.R31T
NM_000518.4:c.93G>C,NP_000509.1:p.R31S
NM_000519.3:c.82G>T,NP_000510.1:p.A28S
NM_000520.4:c.1305C>T,NP_000511.2:p.Y435Y
NM_000520.4:c.412G>A,NP_000511.2:p.G138S
NM_000520.4:c.570G>A,NP_000511.2:p.L190L
NM_000520.4:c.814G>A,NP_000511.2:p.G272R
NM_000521.3:c.1242G>A,NP_000512.1:p.K414K
NM_000521.3:c.1250C>T,NP_000512.1:p.P417L
NM_000521.3:c.299G>T,NP_000512.1:p.R100L
NM_000527.4:c.1061A>C,NP_000518.1:p.D354A
NM_000527.4:c.1061A>G,NP_000518.1:p.D354G
NM_000527.4:c.1503G>A,NP_000518.1:p.A501A
NM_000527.4:c.1586G>A,NP_000518.1:p.G529D
NM_000527.4:c.1813C>T,NP_000518.1:p.L605L
NM_000527.4:c.1845G>A,NP_000518.1:p.E615E
NM_000527.4:c.1875C>T,NP_000518.1:p.N625N
NM_000527.4:c.2000G>A,NP_000518.1:p.C667Y
NM_000527.4:c.2140G>C,NP_000518.1:p.E714Q
NM_000527.4:c.2389G>A,NP_000518.1:p.V797M
NM_000527.4:c.2389G>T,NP_000518.1:p.V797L
NM_000527.4:c.621C>T,NP_000518.1:p.G207G
NM_000530.6:c.276G>A,NP_000521.2:p.V92V
NM_000530.6:c.411C>T,NP_000521.2:p.G137G
NM_000531.5:c.386G>T,NP_000522.3:p.R129L

NM_000531.5:c.540G>C,NP_000522.3:p.Q180H
NM_000531.5:c.663G>A,NP_000522.3:p.K221K
NM_000531.5:c.717G>A,NP_000522.3:p.E239E
NM_000531.5:c.912G>T,NP_000522.3:p.L304F
NM_000532.4:c.1288A>C,NP_000523.2:p.I430L
NM_000532.4:c.653A>G,NP_000523.2:p.K218R
NM_000533.3:c.173A>G,NP_000524.3:p.Y58C
NM_000533.3:c.436C>G,NP_000524.3:p.L146V
NM_000533.3:c.453G>A,NP_000524.3:p.K151K
NM_000533.3:c.453G>C,NP_000524.3:p.K151N
NM_000533.3:c.453G>T,NP_000524.3:p.K151N
NM_000533.3:c.762G>T,NP_000524.3:p.L254L
NM_000535.5:c.825A>G,NP_000526.1:p.Q275Q
NM_000535.5:c.903G>T,NP_000526.1:p.K301N
NM_000537.3:c.689G>A,NP_000528.1:p.R230K
NM_000539.3:c.936G>A,NP_000530.1:p.Q312Q
NM_000540.2:c.11778G>A,NP_000531.2:p.Q3926Q
NM_000540.2:c.14667C>G,NP_000531.2:p.Y4889X
NM_000540.2:c.4934G>A,NP_000531.2:p.R1645Q
NM_000540.2:c.8067G>T,NP_000531.2:p.K2689N
NM_000545.5:c.955G>A,NP_000536.5:p.G319S
NM_000546.4:c.375G>A,NP_000537.3:p.T125T
NM_000546.4:c.375G>C,NP_000537.3:p.T125T
NM_000546.4:c.375G>T,NP_000537.3:p.T125T
NM_000546.4:c.672G>T,NP_000537.3:p.E224D
NM_000547.5:c.1339A>T,NP_000538.3:p.I447F
NM_000547.5:c.1768G>A,NP_000538.3:p.G590S

NM_000547.5:c.2386G>T,NP_000538.3:p.D796Y
NM_000547.5:c.2748G>A,NP_000538.3:p.Q916Q
NM_000547.5:c.349G>C,NP_000538.3:p.D117H
NM_000548.3:c.1118A>C,NP_000539.2:p.Q373P
NM_000548.3:c.1235A>T,NP_000539.2:p.E412V
NM_000548.3:c.1255C>T,NP_000539.2:p.P419S
NM_000548.3:c.1443G>A,NP_000539.2:p.E481E
NM_000548.3:c.2715G>T,NP_000539.2:p.R905R
NM_000548.3:c.2742G>A,NP_000539.2:p.K914K
NM_000548.3:c.3610G>A,NP_000539.2:p.G1204R
NM_000548.3:c.4662G>A,NP_000539.2:p.Q1554Q
NM_000549.3:c.162G>A,NP_000540.2:p.R54R
NM_000551.3:c.462A>C,NP_000542.1:p.P154P
NM_000551.3:c.463G>T,NP_000542.1:p.V155L
NM_000552.3:c.3538G>A,NP_000543.2:p.G1180R
NM_000552.3:c.7056C>T,NP_000543.2:p.G2352G
NM_000552.3:c.7437G>A,NP_000543.2:p.S2479S
NM_000574.3:c.263C>A,NP_000565.1:p.S88X
NM_000574.3:c.596C>T,NP_000565.1:p.S199L
NM_000586.3:c.114G>T,NP_000577.2:p.L38L
NM_000601.4:c.495G>A,NP_000592.3:p.S165S
NM_000610.3:c.1122G>A,NP_000601.3:p.E374E
NM_000617.2:c.1197G>C,NP_000608.1:p.E399D
NM_000767.4:c.516G>T,NP_000758.1:p.Q172H
NM_000767.4:c.777C>A,NP_000758.1:p.S259R
NM_000769.1:c.681G>A,NP_000760.1:p.P227P
NM_000777.3:c.624G>A,NP_000768.1:p.K208K

NM_000781.2:c.566C>T,NP_000772.2:p.A189V
NM_000784.3:c.1016C>T,NP_000775.1:p.T339M
NM_000784.3:c.1017G>C,NP_000775.1:p.T339T
NM_000784.3:c.1183C>A,NP_000775.1:p.R395S
NM_000784.3:c.1184G>T,NP_000775.1:p.R395L
NM_000784.3:c.646G>C,NP_000775.1:p.A216P
NM_000823.3:c.1146G>A,NP_000814.2:p.E382E
NM_000901.4:c.2365G>T,NP_000892.2:p.G789X
NM_000903.2:c.415C>T,NP_000894.1:p.R139W
NM_000929.2:c.185G>A,NP_000920.1:p.W62X
NM_000996.2:c.97G>A,NP_000987.2:p.V33I
NM_001002755.1:c.545G>A,NP_001002755.1:p.R182Q
NM_001005731.1:c.264G>A,NP_001005731.1:p.E88E
NM_001005741.2:c.1505G>A,NP_001005741.1:p.R502H
NM_001007240.1:c.1275A>G,NP_001007241.2:p.Q425Q
NM_001008228.2:c.520A>G,NP_001008229.1:p.I174V
NM_001017980.3:c.272G>C,NP_001017980.1:p.G91A
NM_001018077.1:c.2298T>C,NP_001018087.1:p.N766N
NM_001024630.3:c.1085C>T,NP_001019801.3:p.A362V
NM_001042432.1:c.374G>A,NP_001035897.1:p.S125N
NM_001042599.1:c.1371C>T,NP_001036064.1:p.N457N
NM_001042599.1:c.2892A>G,NP_001036064.1:p.R964R
NM_001074.2:c.1062C>T,NP_001065.2:p.Y354Y
NM_001074.2:c.735A>G,NP_001065.2:p.T245T
NM_001080463.1:c.11747G>A,NP_001073932.1:p.G3916D
NM_001080522.2:c.1762C>T,NP_001073991.2:p.Q588X
NM_001083116.1:c.1620A>G,NP_001076585.1:p.Q540Q

NM_001126054.2:c.2060A>G,NP_001119526.1:p.D687G
NM_001126131.1:c.2617G>T,NP_001119603.1:p.E873X
NM_001128425.1:c.1038G>A,NP_001121897.1:p.S346S
NM_001128425.1:c.690G>A,NP_001121897.1:p.Q230Q
NM_001135243.1:c.2478G>A,NP_001128715.1:p.K826K
NM_001135243.1:c.3156C>T,NP_001128715.1:p.G1052G
NM_001135243.1:c.3612A>C,NP_001128715.1:p.S1204S
NM_001135243.1:c.3613G>A,NP_001128715.1:p.G1205S
NM_001139443.1:c.524T>C,NP_001132915.1:p.V175A
NM_001142459.1:c.810C>T,NP_001135931.2:p.T270T
NM_001142800.1:c.1765A>G,NP_001136272.1:p.R589G
NM_001142800.1:c.5604A>T,NP_001136272.1:p.S1868S
NM_001142800.1:c.5886T>C,NP_001136272.1:p.T1962T
NM_001165963.1:c.1662G>A,NP_001159435.1:p.Q554Q
NM_001165963.1:c.2415G>A,NP_001159435.1:p.L805L
NM_001165963.1:c.2586A>G,NP_001159435.1:p.R862R
NM_001165963.1:c.4476G>A,NP_001159435.1:p.K1492K
NM_001171.5:c.3735G>A,NP_001162.4:p.E1245E
NM_001171.5:c.3735G>T,NP_001162.4:p.E1245D
NM_001171087.1:c.2093G>A,NP_001164558.1:p.G698E
NM_001173466.1:c.251G>A,NP_001166937.1:p.W84X
NM_001173466.1:c.43C>A,NP_001166937.1:p.Q15K
NM_001173990.2:c.230G>C,NP_001167461.1:p.G77A
NM_001182.4:c.834G>A,NP_001173.2:p.V278V
NM_001232.3:c.381C>T,NP_001223.2:p.G127G
NM_001360.2:c.321G>C,NP_001351.2:p.Q107H
NM_001360.2:c.411A>G,NP_001351.2:p.A137A

NM_001369.2:c.1730G>C,NP_001360.1:p.R577T
NM_001369.2:c.6249G>A,NP_001360.1:p.M2083I
NM_001399.4:c.527G>T,NP_001390.1:p.G176V
NM_001399.4:c.741G>A,NP_001390.1:p.Q247Q
NM_001456.3:c.1923C>T,NP_001447.2:p.G641G
NM_001456.3:c.5130C>T,NP_001447.2:p.G1710G
NM_001456.3:c.5193G>A,NP_001447.2:p.T1731T
NM_001456.3:c.7291C>A,NP_001447.2:p.L2431M
NM_001456.3:c.987G>C,NP_001447.2:p.E329D
NM_001609.3:c.1228G>A,NP_001600.1:p.G410S
NM_001681.3:c.2097G>A,NP_001672.1:p.M699I
NM_001681.3:c.324G>C,NP_001672.1:p.Q108H
NM_001735.2:c.1115A>G,NP_001726.2:p.K372R
NM_001735.2:c.4017G>A,NP_001726.2:p.E1339E
NM_001814.4:c.757G>A,NP_001805.3:p.A253T
NM_001814.4:c.890G>T,NP_001805.3:p.G297V
NM_001844.4:c.1962C>T,NP_001835.3:p.G654G
NM_001844.4:c.2862C>T,NP_001835.3:p.G954G
NM_001844.4:c.905C>T,NP_001835.3:p.A302V
NM_001849.3:c.954G>T,NP_001840.3:p.K318N
NM_001852.3:c.186G>A,NP_001843.1:p.P62P
NM_001852.3:c.186G>C,NP_001843.1:p.P62P
NM_001875.4:c.840G>C,NP_001866.2:p.K280N
NM_001918.2:c.1017G>A,NP_001909.2:p.K339K
NM_001918.2:c.939G>A,NP_001909.2:p.K313K
NM_001931.4:c.412G>T,NP_001922.2:p.E138X
NM_001942.2:c.515C>T,NP_001933.2:p.A172V

NM_001953.3:c.1299T>A,NP_001944.1:p.R433R
NM_001985.2:c.375G>C,NP_001976.1:p.Q125H
NM_001999.3:c.3343G>C,NP_001990.2:p.D1115H
NM_002024.5:c.879A>C,NP_002015.1:p.V293V
NM_002049.3:c.220G>C,NP_002040.1:p.V74L
NM_002087.2:c.348A>C,NP_002078.1:p.S116S
NM_002099.6:c.232G>A,NP_002090.4:p.G78R
NM_002100.4:c.208G>T,NP_002091.3:p.V70L
NM_002100.4:c.230C>T,NP_002091.3:p.T77M
NM_002116.7:c.597G>T,NP_002107.3:p.G199G
NM_002116.7:c.619G>A,NP_002107.3:p.D207N
NM_002116.7:c.705G>A,NP_002107.3:p.A235A
NM_002230.2:c.468G>A,NP_002221.1:p.P156P
NM_002256.3:c.103G>A,NP_002247.3:p.G35S
NM_002294.2:c.928G>A,NP_002285.1:p.V310I
NM_002351.4:c.117C>T,NP_002342.1:p.G39G
NM_002351.4:c.201G>A,NP_002342.1:p.E67E
NM_002351.4:c.201G>T,NP_002342.1:p.E67D
NM_002420.4:c.1197G>A,NP_002411.3:p.P399P
NM_002456.5:c.66G>A,NP_002447.4:p.T22T
NM_002542.5:c.137G>A,NP_002533.1:p.R46Q
NM_002734.3:c.177G>A,NP_002725.1:p.K59K
NM_002734.3:c.349G>T,NP_002725.1:p.V117F
NM_002734.3:c.502G>A,NP_002725.1:p.G168S
NM_002734.3:c.550G>A,NP_002725.1:p.V184I
NM_002734.3:c.769G>A,NP_002725.1:p.E257K
NM_002838.3:c.153C>A,NP_002829.2:p.H51Q

NM_002838.3:c.571A>G,NP_002829.2:p.T191A
NM_002878.3:c.345G>C,NP_002869.3:p.Q115H
NM_002977.3:c.406A>G,NP_002968.1:p.I136V
NM_002977.3:c.4382T>C,NP_002968.1:p.I1461T
NM_003000.2:c.540G>A,NP_002991.2:p.L180L
NM_003073.3:c.364G>T,NP_003064.2:p.E122X
NM_003073.3:c.500G>A,NP_003064.2:p.C167Y
NM_003073.3:c.93G>C,NP_003064.2:p.E31D
NM_003124.4:c.304G>T,NP_003115.1:p.G102C
NM_003159.2:c.458A>G,NP_003150.1:p.D153G
NM_003235.4:c.4529G>T,NP_003226.4:p.C1510F
NM_003242.5:c.1524G>A,NP_003233.4:p.Q508Q
NM_003334.3:c.1731C>T,NP_003325.2:p.N577N
NM_003361.2:c.459C>T,NP_003352.2:p.G153G
NM_003494.3:c.1555G>A,NP_003485.1:p.G519R
NM_003494.3:c.5429G>A,NP_003485.1:p.R1810K
NM_003588.3:c.2493G>A,NP_003579.3:p.T831T
NM_003611.2:c.111G>A,NP_003602.1:p.K37K
NM_003611.2:c.111G>C,NP_003602.1:p.K37N
NM_003688.3:c.83G>T,NP_003679.2:p.R28L
NM_003688.3:c.915G>A,NP_003679.2:p.K305K
NM_003722.4:c.1747G>T,NP_003713.3:p.D583Y
NM_003730.4:c.567G>A,NP_003721.2:p.Q189Q
NM_003742.2:c.1388C>T,NP_003733.2:p.T463I
NM_003742.2:c.1445A>G,NP_003733.2:p.D482G
NM_003742.2:c.2494C>T,NP_003733.2:p.R832C
NM_003742.2:c.3003A>G,NP_003733.2:p.R1001R

NM_003742.2:c.3084A>G,NP_003733.2:p.A1028A
NM_003742.2:c.3346G>C,NP_003733.2:p.G1116R
NM_003742.2:c.3556G>A,NP_003733.2:p.E1186K
NM_003742.2:c.3691C>T,NP_003733.2:p.R1231W
NM_003742.2:c.390G>T,NP_003733.2:p.G130G
NM_003742.2:c.500C>T,NP_003733.2:p.A167V
NM_003742.2:c.957A>G,NP_003733.2:p.G319G
NM_003880.3:c.589G>C,NP_003871.1:p.A197P
NM_003900.4:c.1165G>C,NP_003891.1:p.E389Q
NM_003919.2:c.463G>A,NP_003910.1:p.D155N
NM_003919.2:c.662G>A,NP_003910.1:p.G221D
NM_003977.2:c.249G>T,NP_003968.2:p.G83G
NM_003977.2:c.807C>T,NP_003968.2:p.F269F
NM_004006.2:c.10108C>T,NP_003997.1:p.R3370X
NM_004006.2:c.10279C>T,NP_003997.1:p.Q3427X
NM_004006.2:c.1098A>T,NP_003997.1:p.G366G
NM_004006.2:c.1602G>T,NP_003997.1:p.K534N
NM_004006.2:c.1684C>T,NP_003997.1:p.Q562X
NM_004006.2:c.1793C>G,NP_003997.1:p.S598X
NM_004006.2:c.2047G>T,NP_003997.1:p.E683X
NM_004006.2:c.2381A>G,NP_003997.1:p.E794G
NM_004006.2:c.2622G>C,NP_003997.1:p.K874N
NM_004006.2:c.3328G>T,NP_003997.1:p.E1110X
NM_004006.2:c.3432G>A,NP_003997.1:p.Q1144Q
NM_004006.2:c.3603G>A,NP_003997.1:p.K1201K
NM_004006.2:c.3631G>T,NP_003997.1:p.E1211X
NM_004006.2:c.3940C>T,NP_003997.1:p.R1314X

NM_004006.2:c.4518G>A,NP_003997.1:p.V1506V
NM_004006.2:c.5287C>T,NP_003997.1:p.R1763X
NM_004006.2:c.5899C>T,NP_003997.1:p.R1967X
NM_004006.2:c.6117G>A,NP_003997.1:p.K2039K
NM_004006.2:c.6117G>C,NP_003997.1:p.K2039N
NM_004006.2:c.7105G>T,NP_003997.1:p.E2369X
NM_004006.2:c.8390G>C,NP_003997.1:p.R2797T
NM_004006.2:c.8668G>A,NP_003997.1:p.E2890K
NM_004006.2:c.9560A>G,NP_003997.1:p.D3187G
NM_004098.3:c.407G>T,NP_004089.1:p.G136V
NM_004183.3:c.102C>T,NP_004174.1:p.G34G
NM_004285.3:c.960G>A,NP_004276.2:p.V320V
NM_004360.3:c.1008G>T,NP_004351.1:p.E336D
NM_004360.3:c.1137G>A,NP_004351.1:p.T379T
NM_004360.3:c.2161C>G,NP_004351.1:p.L721V
NM_004360.3:c.2195G>A,NP_004351.1:p.R732Q
NM_004360.3:c.715G>A,NP_004351.1:p.G239R
NM_004360.3:c.832G>A,NP_004351.1:p.G278R
NM_004369.3:c.6816G>A,NP_004360.2:p.K2272K
NM_004380.2:c.4559A>G,NP_004371.2:p.K1520R
NM_004429.4:c.30C>T,NP_004420.1:p.G10G
NM_004482.3:c.839G>A,NP_004473.2:p.C280Y
NM_004543.4:c.17376G>C,NP_004534.2:p.Q5792H
NM_004572.3:c.2484C>T,NP_004563.2:p.G828G
NM_004580.4:c.239G>C,NP_004571.2:p.R80T
NM_004629.1:c.1636G>C,NP_004620.1:p.G546R
NM_004646.3:c.1905C>T,NP_004637.1:p.S635S

NM_004656.2:c.1708C>G,NP_004647.1:p.L570V
NM_004698.2:c.1477C>T,NP_004689.1:p.P493S
NM_004698.2:c.1481C>T,NP_004689.1:p.T494M
NM_004700.3:c.648C>T,NP_004691.2:p.R216R
NM_004937.2:c.329G>T,NP_004928.2:p.G110V
NM_004937.2:c.681G>A,NP_004928.2:p.E227E
NM_004984.2:c.217G>A,NP_004975.2:p.D73N
NM_005199.4:c.1249G>C,NP_005190.4:p.E417Q
NM_005359.5:c.1139G>A,NP_005350.1:p.R380K
NM_005422.2:c.5331G>A,NP_005413.2:p.L1777L
NM_005491.3:c.1804C>A,NP_005482.2:p.Q602K
NM_005529.5:c.10248C>T,NP_005520.4:p.S3416S
NM_005529.5:c.10982G>A,NP_005520.4:p.R3661Q
NM_005529.5:c.4740G>A,NP_005520.4:p.S1580S
NM_005529.5:c.8464G>A,NP_005520.4:p.A2822T
NM_005562.2:c.953G>T,NP_005553.2:p.R318M
NM_005566.3:c.244G>A,NP_005557.1:p.D82N
NM_005570.3:c.822G>A,NP_005561.1:p.P274P
NM_005572.3:c.1157G>A,NP_005563.1:p.R386K
NM_005572.3:c.357C>T,NP_005563.1:p.R119R
NM_005572.3:c.513G>A,NP_005563.1:p.K171K
NM_005572.3:c.810G>A,NP_005563.1:p.K270K
NM_005591.3:c.338A>G,NP_005582.1:p.D113G
NM_005591.3:c.658A>C,NP_005582.1:p.R220R
NM_005603.4:c.279G>A,NP_005594.1:p.A93A
NM_005609.2:c.1827G>A,NP_005600.1:p.K609K
NM_005609.2:c.2430C>T,NP_005600.1:p.G810G

NM_005629.3:c.1141G>C,NP_005620.1:p.G381R
NM_005629.3:c.912G>A,NP_005620.1:p.Q304Q
NM_005677.3:c.1281C>T,NP_005668.2:p.C427C
NM_005682.5:c.768G>C,NP_005673.3:p.E256D
NM_005709.3:c.216G>A,NP_005700.2:p.V72V
NM_005910.5:c.853A>C,NP_005901.2:p.S285R
NM_005910.5:c.914G>T,NP_005901.2:p.S305I
NM_005910.5:c.915T>C,NP_005901.2:p.S305S
NM_005910.5:c.945G>A,NP_005901.2:p.L315L
NM_005957.4:c.1166G>A,NP_005948.3:p.W389X
NM_006019.3:c.2236C>T,NP_006010.2:p.Q746X
NM_006019.3:c.713G>T,NP_006010.2:p.C238F
NM_006031.5:c.3840G>C,NP_006022.3:p.Q1280H
NM_006147.3:c.174G>A,NP_006138.1:p.K58K
NM_006147.3:c.175G>T,NP_006138.1:p.A59S
NM_006204.3:c.633G>C,NP_006195.3:p.E211D
NM_006208.2:c.1025G>T,NP_006199.2:p.G342V
NM_006445.3:c.6928A>G,NP_006436.3:p.R2310G
NM_006445.3:c.7000T>A,NP_006436.3:p.Y2334N
NM_006502.2:c.490G>T,NP_006493.1:p.E164X
NM_006846.3:c.2313G>A,NP_006837.2:p.K771K
NM_006846.3:c.55G>A,NP_006837.2:p.D19N
NM_006846.3:c.81G>A,NP_006837.2:p.Q27Q
NM_006846.3:c.891C>T,NP_006837.2:p.C297C
NM_006907.2:c.797G>A,NP_008838.2:p.R266Q
NM_007294.3:c.211A>G,NP_009225.1:p.R71G
NM_007294.3:c.212G>A,NP_009225.1:p.R71K

NM_007294.3:c.3600G>T,NP_009225.1:p.Q1200H
NM_007294.3:c.4185G>A,NP_009225.1:p.Q1395Q
NM_007294.3:c.4484G>T,NP_009225.1:p.R1495M
NM_007294.3:c.4868C>G,NP_009225.1:p.A1623G
NM_007294.3:c.5332G>T,NP_009225.1:p.D1778Y
NM_007294.3:c.5434C>G,NP_009225.1:p.P1812A
NM_007294.3:c.5467G>A,NP_009225.1:p.A1823T
NM_007294.3:c.557C>A,NP_009225.1:p.S186Y
NM_007294.3:c.591C>T,NP_009225.1:p.C197C
NM_007294.3:c.736T>G,NP_009225.1:p.L246V
NM_007294.3:c.786G>A,NP_009225.1:p.Q262Q
NM_007294.3:c.787G>T,NP_009225.1:p.G263C
NM_007294.3:c.788G>C,NP_009225.1:p.G263A
NM_007294.3:c.788G>T,NP_009225.1:p.G263V
NM_007294.3:c.789T>C,NP_009225.1:p.G263G
NM_007294.3:c.790A>T,NP_009225.1:p.S264C
NM_007294.3:c.791G>A,NP_009225.1:p.S264N
NM_007294.3:c.792T>G,NP_009225.1:p.S264R
NM_007315.3:c.2086C>T,NP_009330.1:p.P696S
NM_007315.3:c.372G>C,NP_009330.1:p.Q124H
NM_007315.3:c.603G>T,NP_009330.1:p.K201N
NM_007315.3:c.632A>G,NP_009330.1:p.K211R
NM_012144.2:c.1490G>A,NP_036276.1:p.G497D
NM_012280.2:c.655G>A,NP_036412.1:p.D219N
NM_012414.3:c.3154G>T,NP_036546.2:p.G1052C
NM_012434.4:c.291G>A,NP_036566.1:p.T97T
NM_013382.5:c.1890A>G,NP_037514.2:p.A630A

NM_014080.4:c.2654G>T,NP_054799.4:p.R885L
NM_014141.5:c.1083G>A,NP_054860.1:p.V361V
NM_014208.3:c.52G>T,NP_055023.2:p.V18F
NM_014270.4:c.171C>T,NP_055085.1:p.S57S
NM_014270.4:c.586C>T,NP_055085.1:p.L196L
NM_014585.5:c.1402G>A,NP_055400.1:p.G468S
NM_014639.3:c.751G>A,NP_055454.1:p.G251R
NM_014714.3:c.489C>T,NP_055529.2:p.G163G
NM_014946.3:c.1242A>G,NP_055761.2:p.K414K
NM_014946.3:c.1321G>A,NP_055761.2:p.D441N
NM_014946.3:c.1687G>A,NP_055761.2:p.E563K
NM_014946.3:c.870G>A,NP_055761.2:p.K290K
NM_014946.3:c.870G>T,NP_055761.2:p.K290N
NM_015046.5:c.6106G>A,NP_055861.3:p.G2036R
NM_015294.3:c.860G>A,NP_056109.1:p.S287N
NM_015331.2:c.1768A>G,NP_056146.1:p.S590G
NM_015346.3:c.6011G>C,NP_056161.2:p.S2004T
NM_015506.2:c.276G>T,NP_056321.2:p.E92D
NM_015537.4:c.1432A>G,NP_056352.3:p.T478A
NM_015560.2:c.1074A>G,NP_056375.2:p.A358A
NM_015560.2:c.1515A>G,NP_056375.2:p.K505K
NM_015560.2:c.1770G>C,NP_056375.2:p.R590R
NM_015560.2:c.1770G>T,NP_056375.2:p.R590R
NM_015560.2:c.2707G>C,NP_056375.2:p.V903L
NM_015560.2:c.983A>G,NP_056375.2:p.K328R
NM_015560.2:c.984G>A,NP_056375.2:p.K328K
NM_015629.3:c.319C>G,NP_056444.3:p.L107V

NM_015922.2:c.666C>T,NP_057006.1:p.G222G
NM_016013.2:c.758A>G,NP_057097.2:p.K253R
NM_016124.3:c.1152A>C,NP_057208.2:p.T384T
NM_016124.3:c.1227G>A,NP_057208.2:p.K409K
NM_016239.3:c.7207G>T,NP_057323.3:p.D2403Y
NM_016417.2:c.294A>G,NP_057501.2:p.Q98Q
NM_016489.11:c.339G>C,NP_057573.2:p.W113C
NM_017411.3:c.859G>C,NP_059107.1:p.G287R
NM_017662.4:c.5775A>G,NP_060132.3:p.Q1925Q
NM_017739.3:c.1649G>A,NP_060209.3:p.S550N
NM_017739.3:c.636C>T,NP_060209.3:p.F212F
NM_017777.3:c.1490G>A,NP_060247.2:p.R497K
NM_017777.3:c.417G>A,NP_060247.2:p.E139E
NM_017777.3:c.958G>A,NP_060247.2:p.V320I
NM_017780.3:c.3340A>T,NP_060250.2:p.N1114Y
NM_017780.3:c.5534G>A,NP_060250.2:p.G1845E
NM_017890.3:c.1563G>A,NP_060360.3:p.K521K
NM_018105.2:c.267G>A,NP_060575.1:p.K89K
NM_018136.4:c.3067T>G,NP_060606.3:p.L1023V
NM_018136.4:c.3082G>A,NP_060606.3:p.G1028R
NM_018668.3:c.700G>C,NP_061138.3:p.D234H
NM_019109.4:c.1263G>A,NP_061982.3:p.Q421Q
NM_020166.3:c.1594G>C,NP_064551.3:p.D532H
NM_020247.4:c.993C>T,NP_064632.2:p.F331F
NM_020320.3:c.35A>G,NP_064716.2:p.Q12R
NM_020322.2:c.1608G>A,NP_064718.1:p.P536P
NM_020533.2:c.1406A>G,NP_065394.1:p.N469S

NM_020533.2:c.1704A>T,NP_065394.1:p.G568G
NM_020859.3:c.179G>T,NP_065910.3:p.G60V
NM_020975.4:c.1947G>A,NP_066124.1:p.S649S
NM_021628.2:c.434G>A,NP_067641.2:p.R145H
NM_022098.3:c.1357G>T,NP_071381.1:p.G453C
NM_022124.5:c.1450G>C,NP_071407.4:p.A484P
NM_022124.5:c.3105A>C,NP_071407.4:p.T1035T
NM_022124.5:c.4488G>C,NP_071407.4:p.Q1496H
NM_022124.5:c.5712G>A,NP_071407.4:p.T1904T
NM_022124.5:c.6049G>A,NP_071407.4:p.G2017S
NM_022124.5:c.7872G>A,NP_071407.4:p.E2624E
NM_022132.4:c.1054G>A,NP_071415.1:p.G352R
NM_022132.4:c.803G>C,NP_071415.1:p.R268T
NM_022370.3:c.3319A>C,NP_071765.2:p.S1107R
NM_022455.4:c.4497G>C,NP_071900.2:p.E1499D
NM_022455.4:c.5509G>C,NP_071900.2:p.A1837P
NM_022912.2:c.303G>A,NP_075063.1:p.K101K
NM_022912.2:c.366G>T,NP_075063.1:p.G122G
NM_022912.2:c.595G>C,NP_075063.1:p.G199R
NM_023110.2:c.1081G>C,NP_075598.2:p.A361P
NM_023110.2:c.336C>T,NP_075598.2:p.T112T
NM_023110.2:c.936G>A,NP_075598.2:p.K312K
NM_024296.3:c.330C>T,NP_077272.2:p.F110F
NM_024312.4:c.771G>A,NP_077288.2:p.L257L
NM_024577.3:c.279G>A,NP_078853.2:p.K93K
NM_024649.4:c.479G>A,NP_078925.3:p.R160Q
NM_024675.3:c.2559C>T,NP_078951.2:p.G853G

NM_024753.3:c.711G>T,NP_079029.3:p.R237S
NM_025114.3:c.1824G>A,NP_079390.3:p.K608K
NM_025114.3:c.451C>T,NP_079390.3:p.R151X
NM_025137.3:c.2444G>T,NP_079413.3:p.R815M
NM_025137.3:c.2608A>G,NP_079413.3:p.I870V
NM_025137.3:c.2833A>G,NP_079413.3:p.R945G
NM_030916.2:c.851G>A,NP_112178.2:p.R284Q
NM_030928.3:c.351G>C,NP_112190.2:p.Q117H
NM_031226.2:c.628G>A,NP_112503.1:p.E210K
NM_031443.3:c.288G>A,NP_113631.1:p.K96K
NM_031443.3:c.30G>A,NP_113631.1:p.K10K
NM_031443.3:c.609G>A,NP_113631.1:p.K203K
NM_031885.3:c.117G>A,NP_114091.3:p.K39K
NM_031885.3:c.471G>T,NP_114091.3:p.T157T
NM_033084.3:c.3706C>A,NP_149075.2:p.R1236S
NM_033084.3:c.3707G>A,NP_149075.2:p.R1236H
NM_033084.3:c.376A>G,NP_149075.2:p.S126G
NM_033084.3:c.782A>T,NP_149075.2:p.K261M
NM_033163.3:c.216G>A,NP_149353.1:p.T72T
NM_033305.2:c.7806G>A,NP_150648.2:p.P2602P
NM_033305.2:c.8035G>A,NP_150648.2:p.A2679T
NM_033305.2:c.9474G>A,NP_150648.2:p.R3158R
NM_033453.2:c.94C>A,NP_258412.1:p.P32T
NM_033496.2:c.278G>A,NP_277031.1:p.R93Q
NM_052845.3:c.290G>A,NP_443077.1:p.G97E
NM_052845.3:c.584G>A,NP_443077.1:p.R195H
NM_058172.5:c.1179G>A,NP_477520.2:p.E393E

NM_080877.2:c.756G>A,NP_543153.1:p.Q252Q
NM_080877.2:c.846G>A,NP_543153.1:p.P282P
NM_080916.1:c.591G>A,NP_550438.1:p.Q197Q
NM_130799.2:c.654G>T,NP_570711.1:p.R218R
NM_133433.3:c.3574G>A,NP_597677.2:p.E1192K
NM_133433.3:c.4321G>T,NP_597677.2:p.V1441L
NM_133433.3:c.5427G>T,NP_597677.2:p.R1809S
NM_138694.3:c.2593G>T,NP_619639.3:p.V865F
NM_138694.3:c.657C>T,NP_619639.3:p.G219G
NM_139241.2:c.893T>G,NP_640334.2:p.M298R
NM_139242.3:c.626C>T,NP_640335.2:p.S209L
NM_144605.3:c.474G>A,NP_653206.2:p.V158V
NM_144997.5:c.1300G>A,NP_659434.2:p.E434K
NM_144997.5:c.1300G>C,NP_659434.2:p.E434Q
NM_144997.5:c.1300G>T,NP_659434.2:p.E434X
NM_152701.3:c.11112T>G,NP_689914.2:p.S3704R
NM_153609.2:c.1868G>C,NP_705837.1:p.S623T
NM_153638.2:c.1355A>G,NP_705902.2:p.D452G
NM_153694.4:c.657T>C,NP_710161.1:p.T219T
NM_153704.5:c.2241G>A,NP_714915.3:p.Q747Q
NM_153704.5:c.2439G>A,NP_714915.3:p.A813A
NM_170707.2:c.1821G>A,NP_733821.1:p.V607V
NM_170707.2:c.1868C>G,NP_733821.1:p.T623S
NM_172250.2:c.733G>A,NP_758454.1:p.G245S
NM_173076.2:c.5690G>C,NP_775099.2:p.R1897T
NM_173076.2:c.7436G>A,NP_775099.2:p.R2479K
NM_173660.4:c.414C>T,NP_775931.3:p.L138L

NM_173660.4:c.48C>T,NP_775931.3:p.G16G
NM_176824.2:c.340A>G,NP_789794.1:p.M114V
NM_177438.2:c.2457C>G,NP_803187.1:p.Y819X
NM_177924.3:c.412G>T,NP_808592.2:p.E138X
NM_181714.3:c.955G>A,NP_859065.2:p.A319T
NM_194277.2:c.252G>A,NP_919253.1:p.V84V
NM_194456.1:c.1579G>A,NP_919438.1:p.A527T
NM_194456.1:c.1943C>T,NP_919438.1:p.A648V
NM_194456.1:c.413T>C,NP_919438.1:p.I138T
NM_194456.1:c.712C>T,NP_919438.1:p.L238F
NM_194456.1:c.729G>A,NP_919438.1:p.R243R
NM_198056.2:c.1890G>A,NP_932173.1:p.T630T
NM_198056.2:c.4299G>A,NP_932173.1:p.G1433G
NM_198056.2:c.4719C>T,NP_932173.1:p.G1573G
NM_198309.2:c.459G>A,NP_938051.1:p.T153T
NM_198578.3:c.3342A>G,NP_940980.3:p.L1114L
NM_198843.2:c.501G>T,NP_942140.2:p.G167G
NM_198843.2:c.718G>A,NP_942140.2:p.A240T
NM_199242.2:c.1847A>G,NP_954712.1:p.E616G
NM_203447.3:c.1418A>G,NP_982272.2:p.K473R
NM_206933.2:c.2052A>G,NP_996816.2:p.Q684Q
NM_206933.2:c.2993G>A,NP_996816.2:p.R998K
NM_206933.2:c.4251G>T,NP_996816.2:p.Q1417H
NM_206933.2:c.5776G>A,NP_996816.2:p.E1926K
NM_206933.2:c.949C>A,NP_996816.2:p.R317R
NM_207122.1:c.1174G>A,NP_997005.1:p.A392T
NM_207346.2:c.1251A>G,NP_997229.2:p.P417P

NM_207346.2:c.285G>C,NP_997229.2:p.A95A
NM_207352.3:c.327G>A,NP_997235.3:p.E109E
NM_207517.2:c.4979C>T,NP_997400.2:p.T1660I
NM_213599.2:c.1295C>G,NP_998764.1:p.A432G

Supplementary Table S2. Unseen test set of 352 variants (238 SAVs and 114 SNVs) employed in this study.

HGVS	Class
NM_000132.3:c.6046C>T,NP_000123.1:p.R2016W	SAV
NM_000194.2:c.602A>T,NP_000185.1:p.D201V	SAV
NM_000194.2:c.597C>T,NP_000185.1:p.F199F	SAV
NM_000194.2:c.590A>T,NP_000185.1:p.E197V	SAV
NM_000194.2:c.580G>T,NP_000185.1:p.D194Y	SAV
NM_000194.2:c.551C>T,NP_000185.1:p.P184L	SAV
NM_000194.2:c.544G>A,NP_000185.1:p.E182K	SAV
NM_000194.2:c.539G>T,NP_000185.1:p.G180V	SAV
NM_000194.2:c.538G>A,NP_000185.1:p.G180R	SAV
NM_000194.2:c.482C>A,NP_000185.1:p.A161E	SAV
NM_000194.2:c.143G>A,NP_000185.1:p.R48H	SAV
NM_000194.2:c.119G>T,NP_000185.1:p.G40V	SAV
NM_000533.3:c.409C>T,NP_000524.3:p.R137W	SAV
NM_000533.3:c.409C>G,NP_000524.3:p.R137G	SAV
NM_000052.5:c.3904G>A,NP_000043.3:p.G1302R	SAV
NM_004493.2:c.574C>A,NP_004484.1:p.R192R	SAV
NM_005765.2:c.321C>T,NP_005756.2:p.D107D	SAV
NM_004006.2:c.5434T>A,NP_003997.1:p.F1812I	SAV
NM_004006.2:c.5435T>A,NP_003997.1:p.F1812Y	SAV
NM_004006.2:c.5436C>T,NP_003997.1:p.F1812F	SAV
NM_004006.2:c.5436C>G,NP_003997.1:p.F1812L	SAV
NM_004006.2:c.5436C>A,NP_003997.1:p.F1812L	SAV
NM_004006.2:c.5437A>T,NP_003997.1:p.N1813Y	SAV
NM_000284.3:c.592G>A,NP_000275.1:p.A198T	SAV
NM_000284.3:c.555A>G,NP_000275.1:p.G185G	SAV
NM_000284.3:c.523G>A,NP_000275.1:p.A175T	SAV
NM_000284.3:c.498C>T,NP_000275.1:p.I166I	SAV
NM_000284.3:c.483C>T,NP_000275.1:p.Y161Y	SAV
NM_001250.4:c.408A>T,NP_001241.1:p.T136T	SAV
NM_000022.2:c.643G>A,NP_000013.2:p.A215T	SAV
NM_000527.4:c.1773C>T,NP_000518.1:p.N591N	SAV
NM_000515.3:c.175G>C,NP_000506.2:p.E59Q	SAV
NM_000515.3:c.176A>G,NP_000506.2:p.E59G	SAV
NM_000515.3:c.177A>C,NP_000506.2:p.E59D	SAV
NM_000515.3:c.177A>G,NP_000506.2:p.E59E	SAV
NM_000515.3:c.194A>T,NP_000506.2:p.E65V	SAV
NM_000515.3:c.195A>T,NP_000506.2:p.E65D	SAV
NM_000515.3:c.198G>T,NP_000506.2:p.Q66H	SAV

NM_000515.3:c.200A>G,NP_000506.2:p.K67R	SAV
NM_007294.3:c.5080G>A,NP_009225.1:p.E1694K	SAV
NM_000267.3:c.6792C>T,NP_000258.1:p.Y2264Y	SAV
NM_000267.3:c.946C>A,NP_000258.1:p.L316M	SAV
NM_000267.3:c.945G>A,NP_000258.1:p.Q315Q	SAV
NM_000267.3:c.557A>T,NP_000258.1:p.D186V	SAV
NM_000080.3:c.532T>G,NP_000071.1:p.F178V	SAV
NM_000303.2:c.415G>A,NP_000294.1:p.E139K	SAV
NM_000137.2:c.696C>T,NP_000128.1:p.N232N	SAV
NM_000138.4:c.6354C>T,NP_000129.3:p.I2118I	SAV
NM_002225.3:c.214G>A,NP_002216.2:p.D72N	SAV
NM_002225.3:c.158G>T,NP_002216.2:p.R53L	SAV
NM_002225.3:c.157C>T,NP_002216.2:p.R53C	SAV
NM_001077351.1:c.408G>A,NP_001070819.1:p.R136R	SAV
NM_000321.2:c.1345G>A,NP_000312.2:p.G449R	SAV
NM_000059.3:c.8165C>G,NP_000050.2:p.T2722R	SAV
NM_000059.3:c.620C>T,NP_000050.2:p.T207I	SAV
NM_000059.3:c.617C>G,NP_000050.2:p.S206C	SAV
NM_000059.3:c.599C>A,NP_000050.2:p.T200K	SAV
NM_000059.3:c.599C>T,NP_000050.2:p.T200I	SAV
NM_000059.3:c.587G>T,NP_000050.2:p.S196I	SAV
NM_000059.3:c.587G>A,NP_000050.2:p.S196N	SAV
NM_000059.3:c.581G>A,NP_000050.2:p.W194X	SAV
NM_000059.3:c.572A>T,NP_000050.2:p.D191V	SAV
NM_000059.3:c.559G>A,NP_000050.2:p.E187K	SAV
NM_000059.3:c.521G>A,NP_000050.2:p.R174H	SAV
NM_000190.3:c.66C>G,NP_000181.2:p.R22R	SAV
NM_000051.3:c.6154G>A,NP_000042.3:p.E2052K	SAV
NM_001609.3:c.1165A>G,NP_001600.1:p.M389V	SAV
NM_022494.1:c.618C>T,NP_071939.1:p.T206T	SAV
NM_020975.4:c.1941C>T,NP_066124.1:p.I647I	SAV
NM_000492.3:c.1731C>T,NP_000483.3:p.Y577Y	SAV
NM_000492.3:c.1728A>T,NP_000483.3:p.G576G	SAV
NM_000492.3:c.1728A>G,NP_000483.3:p.G576G	SAV
NM_000492.3:c.1719T>C,NP_000483.3:p.S573S	SAV
NM_000492.3:c.1704G>A,NP_000483.3:p.L568L	SAV
NM_000492.3:c.1698T>A,NP_000483.3:p.A566A	SAV
NM_000492.3:c.1694A>G,NP_000483.3:p.D565G	SAV
NM_000038.5:c.1869G>T,NP_000029.2:p.R623R	SAV
NM_000521.3:c.1481A>G,NP_000512.1:p.D494G	SAV
NM_000344.3:c.840C>T,NP_000335.1:p.F280F	SAV
NM_017411.3:c.885A>C,NP_059107.1:p.X295Y	SAV
NM_017411.3:c.885A>T,NP_059107.1:p.X295Y	SAV

NM_017411.3:c.883T>G,NP_059107.1:p.X295E	SAV
NM_017411.3:c.880A>C,NP_059107.1:p.N294H	SAV
NM_017411.3:c.879A>C,NP_059107.1:p.L293F	SAV
NM_017411.3:c.879A>G,NP_059107.1:p.L293L	SAV
NM_001008388.4:c.109G>C,NP_001008389.1:p.E37Q	SAV
NM_001184.3:c.1995A>G,NP_001175.2:p.E665E	SAV
NM_000249.3:c.1976G>T,NP_000240.1:p.R659L	SAV
NM_000249.3:c.1976G>C,NP_000240.1:p.R659P	SAV
NM_000249.3:c.216T>C,NP_000240.1:p.D72D	SAV
NM_000249.3:c.214G>T,NP_000240.1:p.D72Y	SAV
NM_000249.3:c.214G>C,NP_000240.1:p.D72H	SAV
NM_001875.4:c.2265C>A,NP_001866.2:p.S755S	SAV
NM_000233.3:c.892G>A,NP_000224.2:p.E298K	SAV
NM_002838.4:c.177C>G,NP_002829.3:p.P59P	SAV
NM_000016.4:c.362C>T,NP_000007.1:p.T121I	SAV
NM_005910.5:c.888T>C,NP_005901.2:p.N296N	SAV
NM_005910.5:c.852T>C,NP_005901.2:p.L284L	SAV
NM_005910.5:c.837T>G,NP_005901.2:p.N279K	SAV
NM_000492.3:c.1722T>A,NP_000483.3:p.P574P	SAV
NM_000492.3:c.1719T>G,NP_000483.3:p.S573S	SAV
NM_000492.3:c.1716C>T,NP_000483.3:p.D572D	SAV
NM_000492.3:c.1713A>G,NP_000483.3:p.L571L	SAV
NM_000492.3:c.1357T>A,NP_000483.3:p.L453M	SAV
NM_000492.3:c.1357T>C,NP_000483.3:p.L453L	SAV
NM_000492.3:c.1356G>T,NP_000483.3:p.Q452H	SAV
NM_000492.3:c.1356G>C,NP_000483.3:p.Q452H	SAV
NM_000492.3:c.1355A>C,NP_000483.3:p.Q452P	SAV
NM_000492.3:c.1355A>T,NP_000483.3:p.Q452L	SAV
NM_000492.3:c.1355A>G,NP_000483.3:p.Q452R	SAV
NM_000492.3:c.1354C>A,NP_000483.3:p.Q452K	SAV
NM_000492.3:c.1354C>G,NP_000483.3:p.Q452E	SAV
NM_000492.3:c.1227T>G,NP_000483.3:p.F409L	SAV
NM_000344.3:c.881A>C,NP_000335.1:p.N294T	SAV
NM_000344.3:c.871C>T,NP_000335.1:p.H291Y	SAV
NM_000344.3:c.854A>T,NP_000335.1:p.K285I	SAV
NM_000132.3:c.1569G>T,NP_000123.1:p.L523L	SAV
NM_000116.3:c.525G>A,NP_000107.1:p.V175V	SAV
NM_001456.3:c.5423C>T,NP_001447.2:p.A1808V	SAV
NM_000202.5:c.395C>G,NP_000193.1:p.S132W	SAV
NM_000202.5:c.1122C>T,NP_000193.1:p.G374G	SAV
NM_000074.2:c.256G>T,NP_000065.1:p.E86X	SAV
NM_000194.2:c.209G>T,NP_000185.1:p.G70V	SAV
NM_000061.2:c.119A>G,NP_000052.1:p.Y40C	SAV

NM_000061.2:c.942A>G,NP_000052.1:p.K314K	SAV
NM_004006.2:c.5985T>G,NP_003997.1:p.Y1995X	SAV
NM_000453.2:c.1593C>G,NP_000444.1:p.Y531X	SAV
NM_000215.3:c.1767C>T,NP_000206.2:p.G589G	SAV
NM_006397.2:c.75C>T,NP_006388.2:p.R25R	SAV
NM_006397.2:c.69G>A,NP_006388.2:p.V23V	SAV
NM_000527.4:c.1216C>A,NP_000518.1:p.R406R	SAV
NM_001972.2:c.558C>A,NP_001963.1:p.V186V	SAV
NM_000088.3:c.3790A>G,NP_000079.2:p.M1264V	SAV
NM_000212.2:c.1815C>T,NP_000203.2:p.G605G	SAV
NM_007294.3:c.190T>G,NP_009225.1:p.C64G	SAV
NM_000267.3:c.3831C>T,NP_000258.1:p.G1277G	SAV
NM_000267.3:c.3278T>A,NP_000258.1:p.V1093E	SAV
NM_000267.3:c.2764G>A,NP_000258.1:p.G922S	SAV
NM_000267.3:c.2709G>A,NP_000258.1:p.V903V	SAV
NM_000267.3:c.1466A>G,NP_000258.1:p.Y489C	SAV
NM_000267.3:c.989C>T,NP_000258.1:p.A330V	SAV
NM_004360.3:c.1901C>T,NP_004351.1:p.A634V	SAV
NM_000196.3:c.771C>G,NP_000187.3:p.V257V	SAV
NM_000517.4:c.69C>T,NP_000508.1:p.G23G	SAV
NM_000520.4:c.972T>A,NP_000511.2:p.V324V	SAV
NM_000119.2:c.1747G>T,NP_000110.2:p.E583X	SAV
NM_000431.2:c.75C>T,NP_000422.1:p.G25G	SAV
NM_001017535.1:c.702C>G,NP_001017535.1:p.V234V	SAV
NM_000051.3:c.7865C>T,NP_000042.3:p.A2622V	SAV
NM_000019.3:c.380C>T,NP_000010.1:p.A127V	SAV
NM_006019.3:c.671G>A,NP_006010.2:p.W224X	SAV
NM_000518.4:c.75T>A,NP_000509.1:p.G25G	SAV
NM_000518.4:c.79G>A,NP_000509.1:p.E27K	SAV
NM_000314.4:c.334C>G,NP_000305.3:p.L112V	SAV
NM_194456.1:c.410A>G,NP_919438.1:p.D137G	SAV
NM_194456.1:c.601C>G,NP_919438.1:p.Q201E	SAV
NM_000181.3:c.1617C>T,NP_000172.2:p.S539S	SAV
NM_005514.6:c.360G>C,NP_005505.2:p.Q120H	SAV
NM_022132.4:c.1309A>G,NP_071415.1:p.I437V	SAV
NM_000163.4:c.723C>T,NP_000154.1:p.G241G	SAV
NM_000163.4:c.594A>G,NP_000154.1:p.E198E	SAV
NM_005141.4:c.605T>A,NP_005132.2:p.L202Q	SAV
NM_000297.3:c.2614C>T,NP_000288.1:p.R872X	SAV
NM_005787.5:c.165C>T,NP_005778.1:p.G55G	SAV
NM_001184.3:c.2022A>G,NP_001175.2:p.G674G	SAV
NM_000094.3:c.4965C>T,NP_000085.1:p.G1655G	SAV
NM_000060.2:c.100G>A,NP_000051.1:p.G34S	SAV

NM_000784.3:c.435G>T,NP_000775.1:p.G145G	SAV
NM_003126.2:c.1086A>G,NP_003117.2:p.R362R	SAV
NM_001257374.1:c.1488C>T,NP_001244303.1:p.G496G	SAV
NM_001257374.1:c.1486G>A,NP_001244303.1:p.G496S	SAV
NM_001005741.2:c.750A>G,NP_001005741.1:p.R250R	SAV
NM_033380.2:c.2677G>A,NP_203699.1:p.G893S	SAV
NM_033380.2:c.384G>A,NP_203699.1:p.K128K	SAV
NM_014008.3:c.49A>G,NP_054727.1:p.T17A	SAV
NM_000397.3:c.1152G>C,NP_000388.2:p.K384N	SAV
NM_003560.2:c.1077G>A,NP_003551.2:p.S359S	SAV
NM_006031.5:c.5993A>C,NP_006022.3:p.Q1998P	SAV
NM_022095.3:c.3332G>A,NP_071378.1:p.R1111H	SAV
NM_000214.2:c.886G>A,NP_000205.1:p.D296N	SAV
NM_144577.3:c.742G>A,NP_653178.3:p.A248T	SAV
NM_000528.3:c.2355G>A,NP_000519.2:p.T785T	SAV
NM_001083962.1:c.655G>A,NP_001077431.1:p.D219N	SAV
NM_020964.2:c.1007A>G,NP_066015.2:p.Q336R	SAV
NM_015295.2:c.4566G>A,NP_056110.2:p.T1522T	SAV
NM_001257970.1:c.349G>A,NP_001244899.1:p.G117S	SAV
NM_000342.3:c.1626G>A,NP_000333.1:p.K542K	SAV
NM_007294.3:c.4675G>A,NP_009225.1:p.E1559K	SAV
NM_007294.3:c.5074G>C,NP_009225.1:p.D1692H	SAV
NM_007294.3:c.5408G>C,NP_009225.1:p.G1803A	SAV
NM_001126113.2:c.993G>A,NP_001119585.1:p.Q331Q	SAV
NM_003119.2:c.376G>C,NP_003110.1:p.E126Q	SAV
NM_001077416.1:c.751G>A,NP_001070884.1:p.V251I	SAV
NM_145239.2:c.1011C>T,NP_660282.2:p.G337G	SAV
NM_000517.4:c.94A>C,NP_000508.1:p.R32R	SAV
NM_207036.1:c.825G>C,NP_996919.1:p.L275F	SAV
NM_207036.1:c.822C>G,NP_996919.1:p.R274R	SAV
NM_000138.4:c.2293G>A,NP_000129.3:p.D765N	SAV
NM_005050.3:c.1456G>T,NP_005041.1:p.G486C	SAV
NM_001160147.1:c.1787G>A,NP_001153619.1:p.R596Q	SAV
NM_000131.4:c.66C>T,NP_000122.1:p.G22G	SAV
NM_000059.3:c.7007G>T,NP_000050.2:p.R2336L	SAV
NM_000059.3:c.631G>C,NP_000050.2:p.V211L	SAV
NM_000059.3:c.517G>T,NP_000050.2:p.G173C	SAV
NM_024769.2:c.821G>A,NP_079045.1:p.R274Q	SAV
NM_024649.4:c.1110G>A,NP_078925.3:p.P370P	SAV
NM_130799.2:c.824G>T,NP_570711.1:p.R275M	SAV
NM_000043.4:c.568G>A,NP_000034.1:p.V190M	SAV
NM_001081.3:c.489G>A,NP_001072.2:p.K163K	SAV
NM_001081.3:c.1530G>A,NP_001072.2:p.K510K	SAV

NM_000093.3:c.4068G>T,NP_000084.3:p.A1356A	SAV
NM_139025.3:c.1308G>C,NP_620594.1:p.Q436H	SAV
NM_012127.2:c.790A>G,NP_036259.2:p.S264G	SAV
NM_203447.3:c.2605G>C,NP_982272.2:p.G869R	SAV
NM_015713.4:c.48G>A,NP_056528.2:p.E16E	SAV
NM_017890.4:c.5295G>T,NP_060360.3:p.E1765D	SAV
NM_000441.1:c.1803G>A,NP_000432.1:p.K601K	SAV
NM_006348.3:c.1780G>T,NP_006339.3:p.V594F	SAV
NM_014251.2:c.754G>A,NP_055066.1:p.E252K	SAV
NM_020732.3:c.4110G>A,NP_065783.3:p.P1370P	SAV
NM_001135243.1:c.3183G>A,NP_001128715.1:p.Q1061Q	SAV
NM_058244.2:c.103G>T,NP_490645.1:p.A35S	SAV
NM_032119.3:c.9042G>C,NP_115495.3:p.M3014I	SAV
NM_032380.3:c.1728T>A,NP_115756.2:p.D576E	SAV
NM_000163.4:c.784G>C,NP_000154.1:p.D262H	SAV
NM_006527.2:c.697G>A,NP_006518.1:p.D233N	SAV
NM_015560.2:c.2496G>C,NP_056375.2:p.L832F	SAV
NM_000532.4:c.763G>A,NP_000523.2:p.G255S	SAV
NM_014382.3:c.832G>A,NP_055197.2:p.G278R	SAV
NM_177976.1:c.535G>A,NP_816931.1:p.D179N	SAV
NM_001130964.1:c.1055G>A,NP_001124436.1:p.R352Q	SAV
NM_000249.3:c.986A>C,NP_000240.1:p.H329P	SAV
NM_000249.3:c.299G>C,NP_000240.1:p.R100P	SAV
NM_000393.3:c.1401G>A,NP_000384.2:p.P467P	SAV
NM_001165963.1:c.693A>T,NP_001159435.1:p.P231P	SAV
NM_000537.3:c.98G>A,NP_000528.1:p.R33Q	SAV
NM_000350.2:c.1554G>A,NP_000341.2:p.E518E	SAV
NM_005957.4:c.474A>T,NP_005948.3:p.G158G	SAV
NM_174975.4:c.930T>C,NP_777635.1:p.D310D	SNV
NM_001849.3:c.901G>T,NP_001840.3:p.G301C	SNV
NM_004540.3:c.1049T>C,NP_004531.2:p.L350P	SNV
NM_004715.4:c.1019C>T,NP_004706.3:p.T340M	SNV
NM_181449.2:c.472G>A,NP_852114.2:p.G158R	SNV
NM_001144952.1:c.2376G>A,NP_001138424.1:p.T792T	SNV
NM_000267.3:c.6797G>C,NP_000258.1:p.S2266T	SNV
NM_000267.3:c.6797G>T,NP_000258.1:p.S2266I	SNV
NM_000267.3:c.6797G>A,NP_000258.1:p.S2266N	SNV
NM_000267.3:c.6796A>T,NP_000258.1:p.S2266C	SNV
NM_000267.3:c.6796A>G,NP_000258.1:p.S2266G	SNV
NM_000267.3:c.6796A>C,NP_000258.1:p.S2266R	SNV
NM_000267.3:c.6794A>C,NP_000258.1:p.N2265T	SNV
NM_000267.3:c.6794A>G,NP_000258.1:p.N2265S	SNV
NM_000267.3:c.6794A>T,NP_000258.1:p.N2265I	SNV

NM_000267.3:c.6793A>C,NP_000258.1:p.N2265H	SNV
NM_000267.3:c.6793A>G,NP_000258.1:p.N2265D	SNV
NM_000267.3:c.6793A>T,NP_000258.1:p.N2265Y	SNV
NM_000267.3:c.6791A>C,NP_000258.1:p.Y2264S	SNV
NM_000267.3:c.6791A>G,NP_000258.1:p.Y2264C	SNV
NM_000267.3:c.6791A>T,NP_000258.1:p.Y2264F	SNV
NM_000267.3:c.6790T>A,NP_000258.1:p.Y2264N	SNV
NM_000267.3:c.6790T>C,NP_000258.1:p.Y2264H	SNV
NM_000267.3:c.6790T>G,NP_000258.1:p.Y2264D	SNV
NM_031476.3:c.1329C>T,NP_113664.1:p.A443A	SNV
NM_152336.2:c.852C>T,NP_689549.2:p.D284D	SNV
NM_016642.2:c.4574G>A,NP_057726.3:p.R1525H	SNV
NM_033510.1:c.139C>G,NP_277045.1:p.P47A	SNV
NM_000059.3:c.9213G>T,NP_000050.2:p.E3071D	SNV
NM_000059.3:c.9172A>G,NP_000050.2:p.S3058G	SNV
NM_000059.3:c.8972G>A,NP_000050.2:p.R2991H	SNV
NM_000059.3:c.8962A>G,NP_000050.2:p.S2988G	SNV
NM_000059.3:c.8308G>A,NP_000050.2:p.A2770T	SNV
NM_000059.3:c.627C>A,NP_000050.2:p.L209L	SNV
NM_000059.3:c.627C>T,NP_000050.2:p.L209L	SNV
NM_000059.3:c.623T>G,NP_000050.2:p.V208G	SNV
NM_000059.3:c.619A>G,NP_000050.2:p.T207A	SNV
NM_000059.3:c.602C>G,NP_000050.2:p.P201R	SNV
NM_000059.3:c.582G>A,NP_000050.2:p.W194X	SNV
NM_000059.3:c.575T>C,NP_000050.2:p.M192T	SNV
NM_000059.3:c.573T>C,NP_000050.2:p.D191D	SNV
NM_000059.3:c.551T>C,NP_000050.2:p.L184P	SNV
NM_000059.3:c.549T>C,NP_000050.2:p.S183S	SNV
NM_000059.3:c.539T>C,NP_000050.2:p.I180T	SNV
NM_000059.3:c.534A>G,NP_000050.2:p.K178K	SNV
NM_000059.3:c.532A>C,NP_000050.2:p.K178Q	SNV
NM_000620.4:c.3258C>T,NP_000611.1:p.D1086D	SNV
NM_000192.3:c.505G>A,NP_000183.2:p.G169R	SNV
NM_181486.2:c.709C>T,NP_852259.1:p.R237W	SNV
NM_000192.3:c.710G>A,NP_000183.2:p.R237Q	SNV
NM_000192.3:c.755G>T,NP_000183.2:p.S252I	SNV
NM_001093.3:c.1791T>C,NP_001084.3:p.D597D	SNV
NM_001093771.2:c.615C>T,NP_001087240.1:p.L205L	SNV
NM_017822.3:c.564A>G,NP_060292.3:p.T188T	SNV
NM_021821.3:c.127G>A,NP_068593.2:p.G43R	SNV
NM_003797.3:c.619C>T,NP_003788.2:p.L207L	SNV
NM_002180.2:c.2636C>A,NP_002171.2:p.T879K	SNV
NM_014502.4:c.1047C>T,NP_055317.1:p.S349S	SNV

NM_001244949.1:c.392A>G,NP_001231878.1:p.E131G	SNV
NM_015490.3:c.1026T>C,NP_056305.1:p.H342H	SNV
NM_005077.3:c.354A>G,NP_005068.2:p.E118E	SNV
NM_003382.4:c.825C>T,NP_003373.2:p.N275N	SNV
NM_000492.3:c.1366G>T,NP_000483.3:p.V456F	SNV
NM_000492.3:c.1362G>C,NP_000483.3:p.L454F	SNV
NM_000492.3:c.1360T>A,NP_000483.3:p.L454M	SNV
NM_000492.3:c.1360T>C,NP_000483.3:p.L454L	SNV
NM_000492.3:c.1327G>A,NP_000483.3:p.D443N	SNV
NM_000492.3:c.1281C>A,NP_000483.3:p.S427R	SNV
NM_000492.3:c.1252A>G,NP_000483.3:p.N418D	SNV
NM_000492.3:c.1224A>C,NP_000483.3:p.L408F	SNV
NM_173561.2:c.1320C>T,NP_775832.2:p.C440C	SNV
NM_003122.3:c.194G>A,NP_003113.2:p.R65Q	SNV
NM_173489.4:c.4461C>T,NP_775760.3:p.Y1487Y	SNV
NM_152403.3:c.685T>C,NP_689616.2:p.W229R	SNV
NM_001369.2:c.6264C>T,NP_001360.1:p.A2088A	SNV
NM_015196.3:c.1905C>T,NP_056011.3:p.H635H	SNV
NM_003728.3:c.2162T>C,NP_003719.3:p.M721T	SNV
NM_014208.3:c.53T>A,NP_055023.2:p.V18D	SNV
NM_080685.2:c.6256T>G,NP_542416.1:p.Y2086D	SNV
NM_020041.2:c.844G>A,NP_064425.2:p.V282I	SNV
NM_020865.2:c.1247G>C,NP_065916.2:p.C416S	SNV
NM_015268.3:c.4387G>T,NP_056083.3:p.A1463S	SNV
NM_183061.1:c.2303G>T,NP_898884.1:p.S768I	SNV
NM_000249.3:c.2041G>A,NP_000240.1:p.A681T	SNV
NM_000249.3:c.1961C>T,NP_000240.1:p.P654L	SNV
NM_000249.3:c.1383G>T,NP_000240.1:p.K461N	SNV
NM_000249.3:c.1361G>C,NP_000240.1:p.G454A	SNV
NM_000249.3:c.1339T>C,NP_000240.1:p.L447L	SNV
NM_000249.3:c.1313C>T,NP_000240.1:p.A438V	SNV
NM_000249.3:c.1283A>T,NP_000240.1:p.D428V	SNV
NM_000249.3:c.1270G>C,NP_000240.1:p.A424P	SNV
NM_000249.3:c.1242G>C,NP_000240.1:p.E414D	SNV
NM_000249.3:c.1204A>C,NP_000240.1:p.K402Q	SNV
NM_000249.3:c.1147A>T,NP_000240.1:p.M383L	SNV
NM_000249.3:c.1146G>C,NP_000240.1:p.Q382H	SNV
NM_000249.3:c.1139C>T,NP_000240.1:p.A380V	SNV
NM_000249.3:c.1098G>T,NP_000240.1:p.L366L	SNV
NM_000249.3:c.230G>A,NP_000240.1:p.C77Y	SNV
NM_021198.2:c.402C>T,NP_067021.1:p.H134H	SNV
NM_001204.6:c.818T>G,NP_001195.2:p.M273R	SNV
NM_007366.4:c.3316G>A,NP_031392.3:p.G1106S	SNV

NM_001134224.1:c.258G>A,NP_001127696.1:p.T86T	SNV
NM_001258281.1:c.1589A>G,NP_001245210.1:p.N530S	SNV
NM_000251.2:c.998G>A,NP_000242.1:p.C333Y	SNV
NM_000251.2:c.274C>G,NP_000242.1:p.L92V	SNV
NM_147152.2:c.871G>A,NP_671494.2:p.V291I	SNV
NM_024529.4:c.700C>T,NP_078805.3:p.R234X	SNV
NM_024529.4:c.664C>T,NP_078805.3:p.R222X	SNV
NM_024529.4:c.406A>T,NP_078805.3:p.K136X	SNV
NM_024529.4:c.191T>C,NP_078805.3:p.L64P	SNV
NM_024529.4:c.165C>G,NP_078805.3:p.Y55X	SNV
NM_005562.2:c.297C>T,NP_005553.2:p.S99S	SNV
NM_032872.2:c.1331A>G,NP_116261.1:p.Q444R	SNV
NM_002403.3:c.432T>C,NP_002394.1:p.H144H	SNV
