# Supplementary material for: MutPred Splice: machine learning-based prediction of exonic variants that disrupt splicing
Source: Genome Biol. 2014 Jan 13;15(1):R19. doi: 10.1186/gb-2014-15-1-r19 (PMC4054890; doi:10.1186/gb-2014-15-1-r19)

Supplementary Figure S1. Experimental validation of exon skipping for a true positive MutPred Splice prediction (Mixed negative set, Iter. 3). The disease-causing mutation CM980147 (NM_000051.3: *ATM* c.5932G>T; NP_000042.3: p.E1978X), which is not present in any training data or the unseen evaluation test set, was predicted by MutPred Splice to disrupt splicing. A) Schematic diagram of the exons assayed by RT-PCR. The mutation in exon 41 is indicated. *B*) RT-PCR analysis of spliced mRNA isoforms from mutant or wild type *ATM* genes. This experiment compares splicing of *ATM* pre-mRNA in patient-derived lymphoblastoid cells (E1978X) and HEK293 Cells (wild type). Amplicons derived from different *ATM* mRNA isoforms were by resolved by 1% agarose gel electrophoresis.

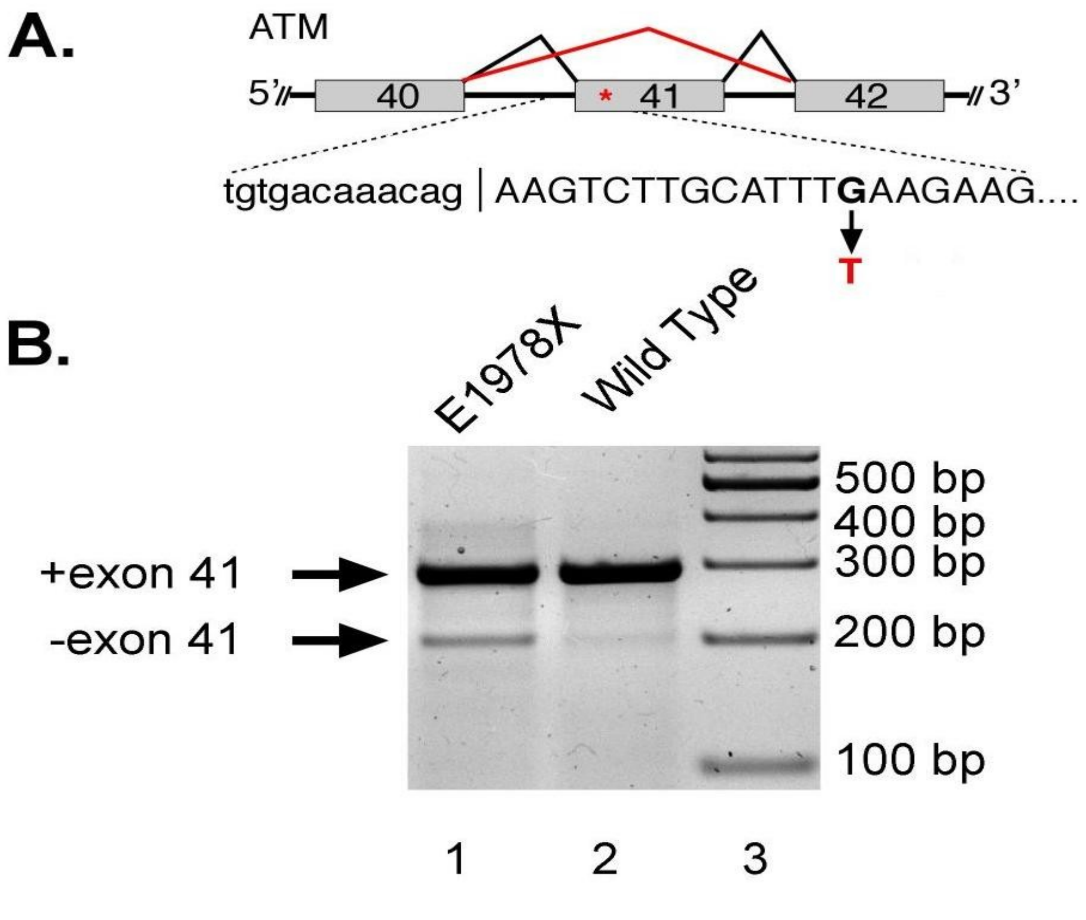

Supplementary Figure S2. Novel ESR hexamer score function (ESR-HS) to express the relationship between disease-causing and common putatively neutral variants and their differential distributions with respect to loss or gain of an ESE or ESS. Frequencies corresponding to disease-causing mutations (red) and common SNPs (blue) are shown. See materials and methods for more details.

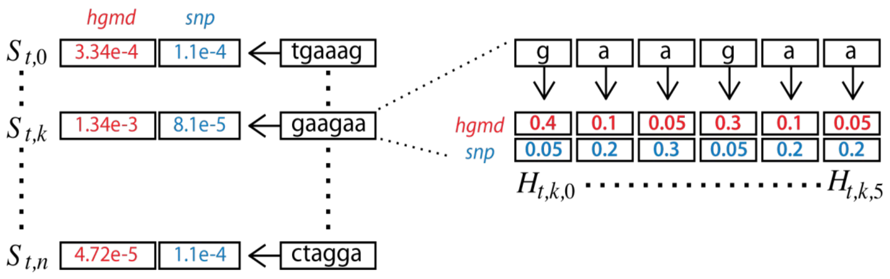

Supplement: Additional file 2: Figure S1 — experimental validation of exon skipping for a true positive MutPred Splice prediction (Mixed negative set, Iter. 3). The disease-causing mutation CM980147 (NM_000051.3: ATM c.5932G > T; NP_000042.3: p.E1978X), which is not present in any training data or the unseen evaluation test set, was predicted by MutPred Splice to disrupt splicing. (A) Schematic diagram of the exons assayed by RT-PCR. The mutation in exon 41 is indicated. (B) RT-PCR analysis of spliced mRNA isoforms from mutant or wild-type ATM genes. This experiment compares splicing of ATM pre-mRNA in patient-derived lymphoblastoid cells (E1978X) and HEK293 cells (wild type). Amplicons derived from different ATM mRNA isoforms were by resolved by 1% agarose gel electrophoresis. Figure S2. novel ESR hexamer score function (ESR-HS) to express the relationship between disease-causing and common putatively neutral variants and their differential distributions with respect to loss or gain of an ESE or ESS. Frequencies corresponding to disease-causing mutations (red) and common SNPs (blue) are shown. See Materials and methods for more details. [file gb-2014-15-1-r19-S2.pdf]
